# A procedure for the estimation over time of metabolic fluxes in scenarios where measurements are uncertain and/or insufficient

**DOI:** 10.1186/1471-2105-8-421

**Published:** 2007-10-30

**Authors:** Francisco Llaneras, Jesús Picó

**Affiliations:** 1 Dept. of Systems Engineering and Control, Technical University of Valencia, Camino de Vera s/n, 46022 Valencia, Spain

## Abstract

**Background:**

An indirect approach is usually used to estimate the metabolic fluxes of an organism: couple the available measurements with known biological constraints (e.g. stoichiometry). Typically this estimation is done under a static point of view. Therefore, the fluxes so obtained are only valid while the environmental conditions and the cell state remain stable. However, estimating the evolution over time of the metabolic fluxes is valuable to investigate the dynamic behaviour of an organism and also to monitor industrial processes. Although Metabolic Flux Analysis can be successively applied with this aim, this approach has two drawbacks: i) sometimes it cannot be used because there is a lack of measurable fluxes, and ii) the uncertainty of experimental measurements cannot be considered. The Flux Balance Analysis could be used instead, but the assumption of optimal behaviour of the organism brings other difficulties.

**Results:**

We propose a procedure to estimate the evolution of the metabolic fluxes that is structured as follows: 1) measure the concentrations of extracellular species and biomass, 2) convert this data to measured fluxes and 3) estimate the non-measured fluxes using the Flux Spectrum Approach, a variant of Metabolic Flux Analysis that overcomes the difficulties mentioned above without assuming optimal behaviour. We apply the procedure to a real problem taken from the literature: estimate the metabolic fluxes during a cultivation of CHO cells in batch mode. We show that it provides a reliable and rich estimation of the non-measured fluxes, thanks to considering measurements uncertainty and reversibility constraints. We also demonstrate that this procedure can estimate the non-measured fluxes even when there is a lack of measurable species. In addition, it offers a new method to deal with inconsistency.

**Conclusion:**

This work introduces a procedure to estimate time-varying metabolic fluxes that copes with the insufficiency of measured species and with its intrinsic uncertainty. The procedure can be used as an off-line analysis of previously collected data, providing an insight into the dynamic behaviour of the organism. It can be also profitable to the on-line monitoring of a running process, mitigating the traditional lack of reliable on-line sensors in industrial environments.

## Background

Fostered by the importance of studying the cell metabolism under a system-level approach [1,2], the set of metabolic pathways of organisms of interest are assembled in metabolic networks [3,4]. If it is assumed that the intracellular metabolites of a network are at pseudo steady-state, mass balances around each metabolite can be described by means of a homogeneous system of linear equations [5]. These equations can be considered as stoichiometric constraints. Then, the constraints imposed by enzyme or transport capacities and thermodynamics (e.g. irreversibility of reactions) can be incorporated to the system [6]. Thereby the imposed constraints define a space where every feasible flux distribution lives [7]. Since the metabolic phenotype can be defined in terms of flux distributions through a metabolic network, this space represents (or at least contains) the set of feasible phenotypes [8]. The environmental conditions given at a certain time instant will determine which of these flux distributions corresponds to the actual one [9].

### Coupling constraints with experimental measurements

Experimental measurements of fluxes can be incorporated as constraints, in order to determine the actual flux distribution or at least to reduce the space of possible flux distributions. However, it must be taken into account that measurements are not invariant constraints, but specific condition constraints [8]. There are several methodologies that use this approach with different purposes: estimate the non-measured fluxes, predict flux distributions, investigate the cell behaviour or monitor bioprocesses.

Metabolic Flux Analysis (MFA) provides a methodology to uniquely determine the actual flux distribution by using a metabolic network and a set of measured fluxes [5]. It has been intensively used in recent years with successful results [10-13]. As it can only consider stoichiometric constraints, a considerable number of fluxes need to be measured to determine the rest of the fluxes. Unfortunately, the available measurements are often insufficient [14].

The Flux Balance Analysis (FBA) can be used to predict metabolic flux distributions [15,16]. Firstly, a constraint-based model is defined as a set of invariant constraints: stoichiometrics, thermodynamics, etc. Then, only a few specific condition constraints (usually substrates uptakes) are imposed. Subject to these constraints, which define a region of possible flux distributions, an optimal flux distribution is calculated using linear programming. Yet, the optimal solution may not correspond to the actual flux distribution. It must be hypothesized that i) the cell has identified the optimal solution, ii) the objective sought by the cell is known, and iii) it can be mathematically expressed. However, FBA predictions based on different objective functions (e.g. maximize growth) are consistent with experimental data [17-19].

### Estimating the evolution over time of flux distributions

Typically, calculation of a flux distribution (e.g. with MFA or FBA) is done under a static point of view: the measured fluxes are assumed to be constant. That means that the obtained flux distribution will only be valid during a certain period of time, while the environmental conditions and the cell state remain steady (e.g. during the growth phase). However, if these conditions change along time, as it happens in an actual culture, the flux distribution will change. The estimation of the flux distribution over time can be useful to investigate the dynamic behaviour of the microorganism or to monitor the progress of industrial fermentations [20]. In [21], the classical FBA is extended to predict the dynamic evolution of flux distributions. In [22], an approach based on elementary modes and the assumption of optimal behaviour is used to estimate the flux distributions of *Corynebacterium glutamicum *at different temporal phases of fermentation. Elementary modes are also employed in [23], where the cell life is decomposed in a succession of phases, and then the time-varying intracellular fluxes are obtained by switching the flux distributions calculated at each phase. In [24], on-line MFA is successfully applied to quantify coupled intracellular fluxes. Takiguchi et al. [25] use a similar approach to recognize the physiological state of the cells culture. They also show how this information can be used to improve Lysine production yield. Very recently [26] has presented an on-line estimation of intracellular fluxes applying MFA to an over-determined metabolic network.

To calculate the succession of flux distributions, it is usually assumed that intracellular fluxes are in quasi-steady state within each measurement step. However, that does not mean that the intrinsic dynamic nature of the cultivation is being disregarded. Instead, the intracellular fluxes will follow the change of environmental conditions as mediated by the measured fluxes (e.g. substrate uptakes). Hence, steady states may undergo shifting from one state to another depending on the evolution of the measured fluxes [27]. Such assumption has been successfully applied in the works cited in above and in the development of several dynamic models [23,28-32]. This approach makes it possible to study the dynamic behaviour of the organism, without considering the still not well-known intracellular kinetics.

### Using the flux spectrum approach to estimate the fluxes

MFA can be successively applied to estimate the evolution of a flux distribution over time. However, this approach has three main difficulties: *i) It cannot be used when measurements are scant *(i.e. when the system is underdetermined). This happens very often due to the lack of measurable fluxes. *ii) The uncertainty of the measured fluxes cannot be considered*. Not only gross errors may appear -which could be managed only in case there are redundant measured fluxes- but also most sources of measurements are intrinsically uncertain and the propagation of this uncertainty to the estimated fluxes is not controlled, and *iii) only equalities can be used as constraints*. For instance, reversibility constraints or maximum flux values cannot be taken into account. FBA solves the first difficulty and provides a framework to deal with the other ones. But the use of FBA in this context could be problematic due to the appearance of a time-variant metabolic objective [22]. For these reasons, the procedure introduced in this work uses the Flux Spectrum Approach (FSA) [33]. It is a variant of MFA that includes some characteristics of FBA (e.g. it is not restricted to stoichiometric constraints) and provides some additional benefits (e.g. it allows to consider measurements uncertainty). The use of FSA will make it possible to face the difficulties described above without assuming an optimal behaviour of the organism.

Although FSA is capable of considering a wide range of constraints, in this work we will only use stoichiometric relationships and simple thermodynamic constraints (reactions directions), and we assume them to be known a priori. However, it must be noticed that the incorporation of thermodynamic constraints -based on measurements or estimations of the standard Gibbs free energy change of reactions- is capturing attention in recent times. A genome-scale thermodynamic analysis of *Escherichia coli *has been recently carried out [34]. Kümmel et al. have introduced an algorithm that -based on thermodynamics, network topology and heuristic rules- automatically assigns reaction directions in metabolic models such that the reaction network is thermodynamically feasible [35]. Interestingly, the reaction directions obtained can be incorporated as constraints before using FSA. Standard Gibbs free energy changes have been also used to incorporate thermodynamic realizability as constraint for FBA [36] -or in an analogous manner to FSA-, and to develop a new form of MFA with the capability of generating thermodynamically feasible fluxes [37].

The objectives of this article are twofold: first, introduce a procedure for the estimation of the metabolic fluxes over time by using a metabolic network as a constraint-based model and a reduced set of measurable species. This procedure is capable of coping with lack of measured species and with its intrinsic uncertainty, thanks to the use of the Flux Spectrum Approach (FSA). Second, illustrate this procedure with a real example: the estimation of non-measured fluxes during a cultivation of CHO cells in batch mode in stirred flasks.

## Results and discussion

### Procedure overview

**Figure 1:**
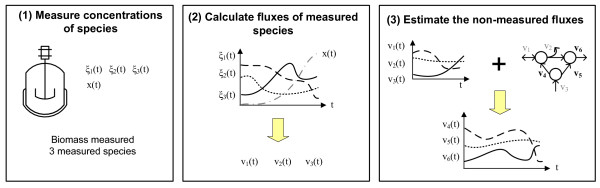
**Procedure overview**. Step 1: get experimental measurements of concentration of some extracellular species and biomass. Step 2: convert this concentrations to measured fluxes. Step 3: estimate the non-measured fluxes by using the Flux Spectrum Approach (FSA). *ξ(t) *is the concentration of an extracellular specie and *v(t) *its flux.*x(t) *is the biomass concentration. Subindexes 1, 2 and 3 denote the measured fluxes and 4, 5 and 6 the non-measured ones.

In most cases, only a few extracellular species are measurable during fermentation processes. This is the reason for use an indirect approach to estimate the fluxes that cannot be measured: couple the available measurements with known biological constraints. Under this philosophy, the proposed procedure is structured as follows (Figure 1): 1) obtain experimental measurements of the concentration of some extracellular species and biomass, 2) convert these concentrations to *measured fluxes *and 3) estimate the non-measured fluxes using the Flux Spectrum Approach (FSA).

It is sometimes overlooked that extracellular fluxes are not directly measured. Instead, the concentrations of a set of species are measured (step 1), and those data are converted to flux units or measured fluxes (step 2). The importance of a good conversion should not be disregarded: error in the measurements of concentrations may be amplified through the conversion, incorporated into the measured fluxes, and then propagated to the estimation of the non-measured fluxes. To minimize this hitch, the conversion should be done carefully. Afterwards, the non-measured fluxes can be estimated by coupling the metabolic network and the measured fluxes (step 3). This has been done before by means of the MFA methodology [24-26]. Yet, this approach has certain limitations. We will overcome some of them using FSA.

It must be remarked that the procedure can be used in two main scenarios: as an off-line analysis of previously collected data or as an on-line monitoring of an industrial process. The structure of the procedure and its fundamental step (step 3) are exactly the same in both cases. Nevertheless, there are several differences concerning step 2. These differences will be briefly described along the article and illustrated in an additional file [additional file 3].

### Preliminaries: choice and analysis of the metabolic network

A metabolic network can be represented with a stoichiometric matrix *S*, where rows correspond to the *m *metabolites and columns to the *n *fluxes. Assuming that the intracellular metabolites are at pseudo-steady state, material balances around them can be formulated as follows [38,39]:

*S*·**v **= 0

where **v **is a flux distribution. Assuming that *S *has full row rank, the number of independent equations is *m*. As typically *n *becomes larger than *m*, the system (1) is underdetermined (*n-m *degrees of freedom). That means that there is not a unique flux distribution fulfilling (1), but an infinite number of feasible flux distributions. In order to determine which of these feasible flux distributions is the current one, the constraints imposed by the measured fluxes will be incorporated -latter on it will be shown that other constraints, for example the reversibility constraints, can be added.

Thereby, when choosing the metabolic network to be used through the procedure, it must be taken into account that its degree of detail needs to be compatible with the number of available measurements -i.e. the available measurements must be sufficient to offset the underdeterminacy of the network. In order to study this, we can analyze the system formed by the stoichiometric constraints given by (1) and the constraints imposed by the measured fluxes. This system -which constitutes the fundamental equation of MFA- can be obtained making a partition between measured (subindex *m*) and non-measured or unknown fluxes (subindex *u*):

*S*_*u*_·**v**_**u **_= -*S*_*m*_·***v***_**m**_

#### System Determinacy and Calculability of Fluxes

System (2) is determined when there are enough linearly independent constraints for uniquely calculate all non-measured fluxes **v**_**u**_; i.e., when *rank*(*S*_*u*_) = *u *(*u *is the number of non-measured fluxes). On the contrary, when *rank*(*S*_*u*_)>*u*, the system is classified as underdetermined because at least one non-measured flux, and probably most of them, is non calculable [14]. If the system is underdetermined, the traditional MFA methodology cannot be used to calculate the non-measured fluxes. Fortunately, the use of FSA may provide an estimation of the non-measured fluxes even in this situation. However, it must be taken into account that the likelihood of obtaining a precise estimation increases as the underdeterminancy reduces, as the set of flux distributions compatible with the measured values will be smaller.

#### System Redundancy and Consistency of Measurements

System (2) is redundant when some rows in *S*_*u *_can be expressed as linear combinations of other rows; i.e., when *rank*(*S*_*u*_)<*m*. This can lead to an inconsistent system if the vector **v**_**m **_contains such values that no **v**_**u **_exists that exactly solves (2). Therefore, when the system is redundant, the inconsistency of the measurements can be checked and its importance can be estimated (see methods). Unfortunately some measured fluxes have no impact on the consistency of the system, so they cannot be considered in the analysis of consistency. These fluxes are called non-balanceable. The balanceable fluxes can be detected as explained in [14], and they should be adjusted (or balanced) in case the system is inconsistent (see methods). All these methods are commonly applied when MFA is used [12,24,26]. They can also be used within our procedure, but in addition the use of FSA provides new methods to deal with inconsistency as it will be shown in a subsequent section.

### Step 1: Getting experimental measurements of species

There are several alternatives to measure the concentration of species -e.g. on-line sensors, isotopic tracer experiments or laboratory procedures- but providing a detailed description of each one is out of the scope of this work. In any case, it must be remembered that the more measurements are available, the more non-measured fluxes may be accurately estimated. However, it is necessary to be prepared to overcome a lack of measurements, especially when the procedure is done on-line (due to the lack of reliable on-line sensors).

### Step 2: conversion of measured concentrations in measured fluxes

A mass balance around each extracellular species whose concentration is measurable can be stated as:

dξdt=vξ⋅X−D⋅ξ+Fξ
 MathType@MTEF@5@5@+=feaafiart1ev1aaatCvAUfKttLearuWrP9MDH5MBPbIqV92AaeXatLxBI9gBaebbnrfifHhDYfgasaacH8akY=wiFfYdH8Gipec8Eeeu0xXdbba9frFj0=OqFfea0dXdd9vqai=hGuQ8kuc9pgc9s8qqaq=dirpe0xb9q8qiLsFr0=vr0=vr0dc8meaabaqaciaacaGaaeqabaqabeGadaaakeaadaWcaaqaaiabdsgaKHGaciab=57a4bqaaiabdsgaKjabdsha0baacqGH9aqpcqWG2bGDlmaaBaaabaGae8NVdGhabeaakiabgwSixlabdIfayjabgkHiTiabdseaejabgwSixlab=57a4jabgUcaRiabdAeagTWaaSbaaeaacqWF+oaEaeqaaaaa@4472@

where *ξ *is the specie concentration, *v*_*ξ *_its flux (substrate uptake or product formation), *X *the biomass concentration,*D *the dilution term and *F*_*ξ *_the net exchange of the specie with the outside. Notice that this equation is only valid for extracellular species; however, the biomass growth and the mass balance around an internal metabolite not assumed to be at pseudo-steady state can be represented in a similar way [40,41].

It is possible to calculate *v*_*ξ *_as a function of *ξ*, *X*, *D*, *F*_*ξ *_and *dξ/dt*. But this presents two main difficulties: i) approximate a derivative (directly or indirectly) and ii) deal with the presence of errors and noise in the measurements of the concentration *ξ *[42]. The underlying problem is how precision can be combined with robustness with respect to measurement errors. The most straightforward approach is to approximate the derivative with a simple method (e.g. Euler or Runge-Kutta methods) and then solve (3) [42]. Very often this straight approximation needs to be combined with the use of filters to eliminate -or at least to reduce- the presence of noise. This approach provides very good results when centred methods can be used to approximate the derivative and to filter the resultant signal, i.e. when the whole procedure is done off-line, or when it is done on-line but certain delay in the calculation of the fluxes is allowable (i.e. when past, *k-i*, and future information, *k+i*, is available for the calculation of *v*_*ξ*_*(k)*). Furthermore, there are methods especially aimed to the on-line approximation of the derivative. If the noise signal is well characterized (e.g. the frequency band or a stochastic feature is known) a linear differentiator [43] or even a Luenberger observer may be used [44]. If nothing is known on the structure of the signal, then sliding mode techniques are profitable. For example, the method introduced in [45] combines exact differentiation for a large class of input signals with robustness with respect to any small noises. Finally, there are other approaches to calculate the extracellular fluxes that avoid the approximation of the derivative, as for example the use of extended Kalman filters [26,46] or the observers based on concepts from nonlinear systems theory, such as the high gain estimators described in [40,47]. These methods do not use future information because they are aimed to the on-line operation mode.

The importance of the use of filters should be remarked: not only the signal of measured concentrations should be filtered to reduce its noise, but also the calculated extracellular fluxes may be filtered to get a smooth signal. Filters based on the moving average will be used in this work since they are simple and versatile. Basically, the filtered value at time *k *is calculated by averaging the values of the original signal within a time window. There are several versions that differ in the time window used (backward or centred) and in the distribution of weight over the averaged values (uniform or exponential). Interestingly, this kind of filters has already been successfully applied to the calculation of metabolic fluxes [42].

**Figure 2:**
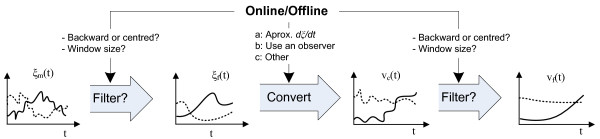
**Conversion of measured concentrations to measured fluxes**. First, the measured concentrations should be filtered. Then, fluxes are calculated from the concentration data (e.g. approximating the derivative or using a dynamic observer). Finally, the calculated fluxes may be filtered to get a smooth signal. Each step is conditioned by the operation mode (on-line or off-line).

To provide a complete description of our procedure, two conversion approaches are described in the methods section: the combination of an Euler method with a moving average filter, and the use of a nonlinear observer (see Figure 2). The first one is especially suitable when the procedure is done off-line, while the second one is aimed to work on-line. Nevertheless, it must be taken into account that there is not a universal solution for the conversion problem. In real applications, the particularities of the concentrations measurements (accuracy, sample rate, importance and characteristics of the noise, etc.) and the operation mode (off-line, on-line with an acceptable delay or purely on-line) will determine which method is the most suitable one. A real off-line conversion is described below, but the most illustrative example of the step 2 is given in the Additional File 3, which addresses the on-line and the off-line operation modes and the use of filters. A practical guide about step 2 is also given in the mentioned file.

### Step 3: estimation of the non-measured fluxes using FSA

Finally, the measured fluxes obtained in step 2 are coupled with known biological constraints in order to estimate the non-measured fluxes (Figure 1). Basically, this implies that a solution for system (2) has to be found at each time instant *k*. Traditionally MFA was successively applied with this purpose. Unfortunately, as mentioned in the background section, this has some limitations -which become especially critical if the procedure is done on-line, due to the traditional lack of reliable on-line sensors. To overcome them, the Flux Spectrum Approach (FSA) will be used instead in the third step of our procedure.

Using FSA, the estimation of the non-measured fluxes at each time instant *k *is obtained as follows [33]: 3.1) impose the set of constraints given by (2) and the reversibility constraints. They define a region where the actual fluxes may live. 3.2) calculate the interval of possible values for each non-measured flux by solving two linear programming problems, one to compute its maximum value within the region and the other one to compute its minimum (details are given in the methods section). Thus, at each time *k*, and for each non-measured flux, an interval bracketing its possible values will be obtained: *v*_*uj*_*(k) *= [*v*_*uj, min*_, *v*_*uj, max*_]. The size of the intervals (i.e. the imprecision of the estimation) depends on the number of non-measured fluxes, the irreversible reactions, the available measurements and the degree of uncertainty considered. Of course, the more constraints are available, the tighter intervals are obtained. If uncertainty is not considered, reversibility constraints are not used, and the system (2) is determined, FSA gives the same unique solution as MFA [33]. But in addition, the use of FSA provides several advantages to the estimation procedure (see Table 1):

**Table 1:** Comparison between MFA and FSA

	**Traditional Metabolic Flux Analysis (MFA)**		**Flux Spectrum Approach (FSA)**	
	*Flux estimation*	*Consistency*	*Flux estimation*	*Consistency*
*Determined*	Yes.	Consistency check χ^2^.	Yes.	Consistency check χ^2^.
*Redundant*		Flux adjustment.	Considers uncertainty.	Flux adjustment or use of a band of uncertainty.
			Detects sensitivity problems.	
*Determined*	Yes.	No.	Yes.	Detect some inconsistencies.
*Not Redundant*		No.	Considers uncertainty.	
			Detects sensitivity problems.	
*Underdetermined*	No.	Consistency check χ^2^.	Yes (not guaranteed).	Consistency check χ^2^.
*Redundant*		Flux adjustment.	Considers uncertainty.	Flux adjustment or use of a band of uncertainty.
			Detects sensitivity problems.	
*Underdetermined*	No.	No.	Yes (not guaranteed).	Detect some inconsistencies.
*Not Redundant*		No.	Considers uncertainty.	
			Detects sensitivity problems.	

**Figure 3:**
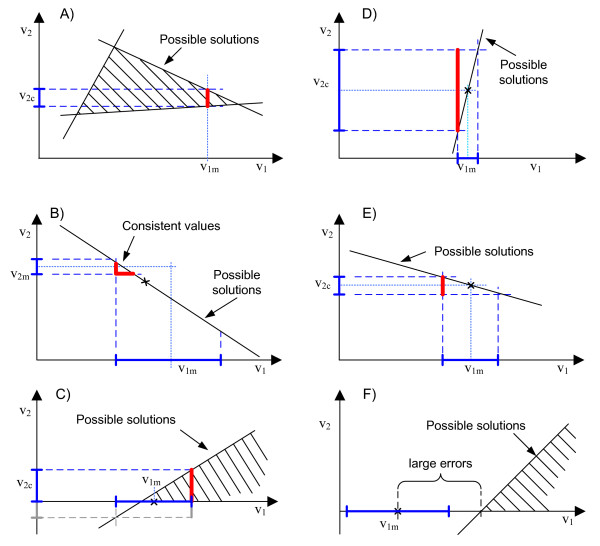
**Flux spectrum approach in use**. Each figure shows a schematic projection of a high-dimensional flux space into two dimensions. The space of possible solutions (before taken into account *v*_*1 *_and *v*_*2*_) is represented by a polygon or a solid black line and tagged with a label. Subindex *m *denotes a measured flux, and *c *a calculated one. The band of uncertainty around measured fluxes is represented with a blue, solid interval in the axis. The estimations provided by FSA are represented with red, thick lines. Dotted lines are just auxiliary projections. (A) Underdetermined case. The interval of possible values for *v*_*2 *_is computed even when the system is underdetermined. (B) Determined and redundant case. Both fluxes are measured, but its values are inconsistent. With the band of uncertainty used, all the values of *v*_*1 *_and *v*_*2 *_within the red line are considered valid. Due to the shape of the band, the values given by a least squares adjustment (denoted with an x) are not considered as a valid solution. (C) Reversibility constraints. A reversibility constraint reduces the interval of possible values for *v*_*2*_. (D) Detection of sensitivity problems. The strangely large interval of possible values for *v*_*2 *_may indicate a problem of sensitivity. (E) Representation of uncertainty. A non-measured flux is estimated from an uncertain measurement. (F) Detection of large errors. A large error in the measured flux *v*_*1m *_is detected with the reversibility constraint.

• It makes it possible to consider the uncertainty of experimental measurements and even qualitative knowledge (e.g. maximum values of certain fluxes). Hence, if measurements uncertainty is indeed present and it is well characterized, the estimation of non-measured fluxes will be more reliable (Figure 3E). FSA provides not only a prediction of the fluxes, but also an indication of the reliability of this prediction.

• It considers the reversibility constraints of certain fluxes. This provides an estimation of the non-measured fluxes even when measurements are insufficient, i.e. when (2) is underdetermined (Figure 3A). This estimation will be precise if the degree of underdeterminancy is limited and there are irreversible fluxes. On the contrary, the estimation could be poor and some intervals may be unbounded. The reversibility constraints will also restrict the intervals of the estimated fluxes when uncertainty is considered (Figure 3C). Finally, the reversibility constraints can also provide a means to detect inconsistencies even when the system is not redundant (Figure 3F).

• It provides a straight method for coping with inconsistency: a band of uncertainty is used instead of adjusting the inconsistent measurements. As any inconsistent set of measurements is necessarily uncertain, it seems reasonably to define a band of uncertainty around the measured values trying to enclose nearby consistent sets of measurements. Thus, every consistent set of measurements enclosed by the band will be taken into account in the estimation of the non-measured fluxes (Figure 3B). Furthermore, the band size needed to find the nearest consistent flux distribution gives an indication of the degree of inconsistency.

Additional advantages arise when FSA is used in a successive way to estimate the temporal evolution of the metabolic fluxes:

• It may detect sensitivity problems. Assume that a band of uncertainty is being used and that the measured fluxes change smoothly over time. If the interval of values for an estimated flux is strangely large at a certain instant *k*, it indicates that a slight change in the measured fluxes has a big effect over the estimated flux, i.e. that a sensitivity problem exists (Figure 3D). Thereby, an analysis of sensitivity is incorporated in the estimation procedure.

• The peak values at certain time instants *k *-which may appear when MFA is used- are avoided with FSA. These peaks are consequence of slight errors in the measurements (which are common due to the lack of reliable sources of measurements and due to the uncertainty of the conversion of concentration data into measured fluxes). Since FSA considers a band of uncertainty around the measured values, it avoids, or at least reduces, this phenomenon.

• The estimation given by FSA for a certain flux at time *k *(an interval of possible values), combined with the inspection of past and future estimations and with our qualitative knowledge about cell behaviour, may be used to hypothesize which of the possible temporal evolutions corresponds to the actual one. That is to say, the richness of the estimation given by FSA makes it possible to exploit our qualitative knowledge to support certain hypothesis without being confused by measurements uncertainty.

### Application: estimation of the fluxes during a cultivation of CHO cells

The three-step procedure described in the previous section is now applied to a real problem taken from the literature: the estimation of the intracellular fluxes of CHO cells cultivated in batch mode in stirred flasks. The available experimental data are the typical data measured off-line (accurate measurements of the concentration of a few species but with a low sample rate), and therefore this example will be approached assuming that the procedure is done off-line. This assumption is important during the second step of the procedure, and for this reason an example has been included in the Additional File 3 that illustrates the differences between the on-line and the off-line operation modes. However, hereinafter we will pay special attention to the third step of the procedure because it is the most important one. In particular, the benefits provided by the use of FSA will be compared with those obtained with the well-established MFA methodology, which is the basis of most of the similar procedures [24-26]. This comparison illustrates the advantages of the new estimation procedure in three different scenarios:

S1. When measurements are *almost *sufficient. The number of measured fluxes is almost sufficient when there are enough to determine all the non-measured fluxes but there are not redundant measurements (i.e. when the system (2) is determined and not redundant).

S2. When measurements are sufficient, i.e. when the measured fluxes are enough to determine the non-measured fluxes and there are also redundant measurements (the system (2) is determined and redundant).

S3. When measurements are insufficient. The number of measured fluxes is insufficient when there are not enough to determine all the non-measured fluxes (i.e. when the system (2) is underdetermined and not redundant).

For completeness, the most uncommon case (when the system is underdetermined but redundant) is illustrated with a toy example in an appendix [Additional File 2]. In the three scenarios, the intrinsic uncertainty of the measured fluxes is taken into account.

#### Metabolic network of CHO Cells

**Figure 4:**
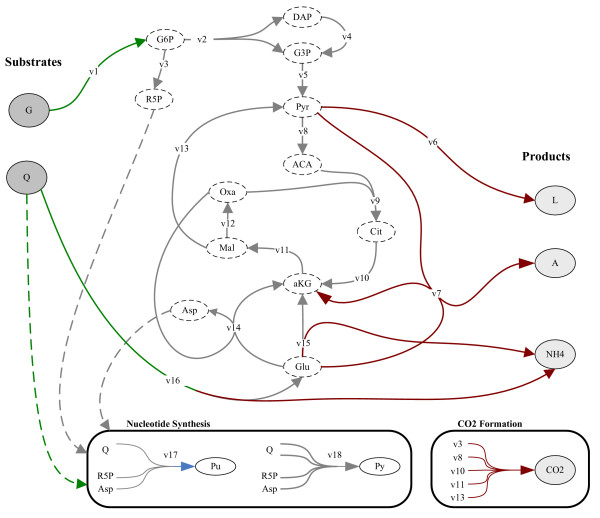
**Metabolic Network of CHO cells**. Extracted from [41]. Initial substrates (dark grey ovals), extracellular products (light grey ovals), terminal internal metabolites (white ovals) and internal metabolites (white ovals with dashed line). The CO_2 _formation and the nucleotide synthesis are described separately. The nomenclature is given in the additional file 1.

The metabolic network (Figure 4) has been taken from [48]. The network describes only the metabolism concerned with the two main energetic nutrients, glucose and glutamine. Thus, the metabolism of the amino-acids provided by the culture medium is not included. Four pathways are considered: the glycolysis, the glutaminolysis, the TCA cycle and the nucleotides synthesis. All reactions are assumed to carry flux only in only one direction, except reactions 2, 4, 5, 6 and 7 that are reversible (e.g. when glucose is exhausted lactate and alanine are consumed instead of produced). The complete lists of species and reactions are given in the Additional File 1.

The mass balance around intracellular metabolites at pseudo-steady state is given by eq. 1 (the stoichiometric matrix *S *is given in the Additional File 1). There are 12 metabolites (*m*) and 18 intracellular fluxes (*n*). Therefore, the system is underdetermined and has 6 degrees of freedom. The extracellular fluxes *v*_*G*_, *v*_*L *_and *v*_*A *_coincide with the fluxes -*v*_*1*_, *v*_*6 *_and *v*_*7*_. Three equations that link *v*_*NH4*_, *v*_*Q *_and *v*_*CO2 *_with the intracellular fluxes can be obtained by inspection of the metabolic network. Moreover, it is a natural assumption to consider that the formation of purine and pyrimidine nucleotides is the same. As a result, four equations are incorporated by the authors [48]:

vNH4:v19=v15+v16−vQ:v20=v16+v17+2⋅v18vCO2:v21=v3+v8+v10+v11+v13vExtra:v22=0=v17−v18
 MathType@MTEF@5@5@+=feaafiart1ev1aaatCvAUfKttLearuWrP9MDH5MBPbIqV92AaeXatLxBI9gBaebbnrfifHhDYfgasaacH8akY=wiFfYdH8Gipec8Eeeu0xXdbba9frFj0=OqFfea0dXdd9vqai=hGuQ8kuc9pgc9s8qqaq=dirpe0xb9q8qiLsFr0=vr0=vr0dc8meaabaqaciaacaGaaeqabaqabeGadaaakqaabeqaaiabdAha2TWaaSbaaWqaaiabd6eaojabdIeaiHqaaiab=rda0aqabaGccqGG6aGocqWG2bGDdaWgaaWcbaGaeGymaeJaeGyoaKdabeaakiabg2da9iabdAha2naaBaaaleaacqaIXaqmcqaI1aqnaeqaaOGaey4kaSIaemODay3aaSbaaSqaaiabigdaXiabiAda2aqabaaakeaacqGHsislcqWG2bGDlmaaBaaameaacqWGrbquaeqaaOGaeiOoaOJaemODay3aaSbaaSqaaiabikdaYiabicdaWaqabaGccqGH9aqpcqWG2bGDdaWgaaWcbaGaeGymaeJaeGOnaydabeaakiabgUcaRiabdAha2naaBaaaleaacqaIXaqmcqaI3aWnaeqaaOGaey4kaSIaeGOmaiJaeyyXICTaemODay3aaSbaaSqaaiabigdaXiabiIda4aqabaaakeaacqWG2bGDlmaaBaaameaacqWGdbWqcqWGpbWtcqWFYaGmaeqaaOGaeiOoaOJaemODay3aaSbaaSqaaiabikdaYiabigdaXaqabaGccqGH9aqpcqWG2bGDdaWgaaWcbaGaeG4mamdabeaakiabgUcaRiabdAha2naaBaaaleaacqaI4aaoaeqaaOGaey4kaSIaemODay3aaSbaaSqaaiabigdaXiabicdaWaqabaGccqGHRaWkcqWG2bGDdaWgaaWcbaGaeGymaeJaeGymaedabeaakiabgUcaRiabdAha2naaBaaaleaacqaIXaqmcqaIZaWmaeqaaaGcbaGaemODay3cdaWgaaadbaGaemyrauKaemiEaGNaemiDaqNaemOCaiNaemyyaegabeaakiabcQda6iabdAha2naaBaaaleaacqaIYaGmcqaIYaGmaeqaaOGaeyypa0JaeGimaaJaeyypa0JaemODay3aaSbaaSqaaiabigdaXiabiEda3aqabaGccqGHsislcqWG2bGDdaWgaaWcbaGaeGymaeJaeGioaGdabeaaaaaa@8E44@

These constraints can be represented with a *4×18 *matrix *S*_*ξ *_fulfilling (11). Then, (11) and (1) can be joined to define an extended homogeneous system of linear equations (see methods). The extended system has 16 metabolites (*mx*) and 22 reactions (*nx*).

The mathematical model, formed by the stoichiometric matrixes S and S_ξ _, is given in a Matlab script and a standard SBML file [see Additional File 4].

#### Step 1: getting experimental measurements of species

**Figure 5:**
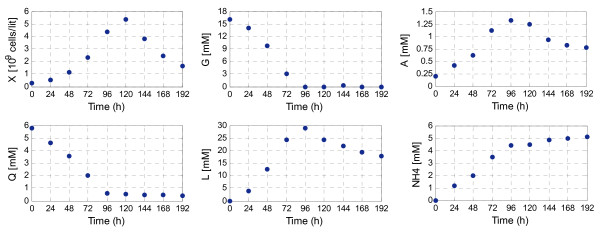
**Concentration of measured extracellular species and biomass during a cultivation of CHO cells**. The measurements correspond to cell density (X), glucose (G), glutamine (Q), lactate (L), alanine (A) and ammonia (NH4).

The experimental data taken from [28] is given in Figure 5. The cell density (X) and the concentration of 5 extracellular species are measured; two substrates, glucose (G) and glutamine (Q), and three excreted products, lactate (L), alanine (A) and ammonia (NH4). This data was collected with a sample rate of 24 h. These measurements cannot be filtered because -due to the low sample rate- it is impossible to distinguish between noise and true changes of the signal.

#### Step 2: conversion of measured concentrations in measured fluxes

**Figure 6:**
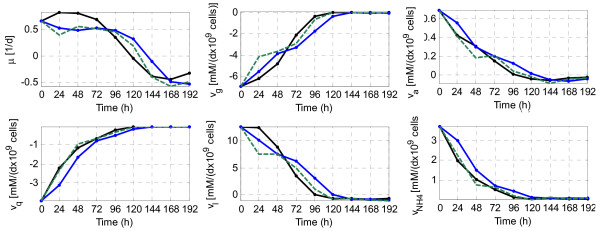
**Extracellular fluxes and growth rate calculated from the measured concentrations**. *μ *is the biomass growth rate, *v*_*g*_, the flux of glucose, *v*_*Q *_the flux of glutamine, *v*_*L *_the flux of lactate, *v*_*A *_the flux of alanine and *v*_*NH*4 _the flux of ammonia. Fluxes are calculated with the middle point Euler approximation (black solid line) and the backward Euler approximation (green dotted line). In addition, fluxes calculated with the backward Euler approximation and filtered with a standard moving average of order 2 are also depicted (blue solid line).

The second step of the procedure is the conversion of the measured concentrations in measured fluxes. The measured fluxes (and the biomass growth) calculated with three different approximations of the derivative are depicted in Figure 6 (see methods). Since the procedure is being done off-line, a centred approximation is the most advisable choice. Therefore, the fluxes calculated with the middle point Euler approximation will be used hereinafter. We obtained similar results (not shown) when the complete example was done using a backward Euler approximation (which would be more suitable in case the procedure were done on-line). It is also remarkable that Figure 6 already gives the idea of uncertainty -differences between the conversions obtained with different methods are significant. In fact, the different conversions, along with the precision of the sensors and the protocols used to measure the concentration of species, could be used to characterize the uncertainty in the measured fluxes.

#### Step 3 (S1): estimation of fluxes if measurements are almost sufficient and uncertain

**Figure 7:**
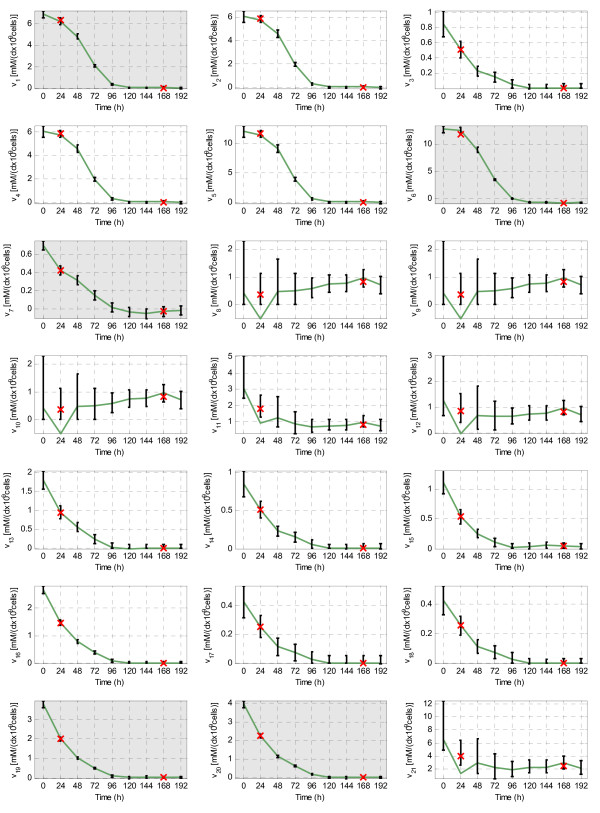
**FSA and MFA in the determined and not redundant case (S1)**. Known fluxes are: *v*_*1*_*(G)*, *v*_*6*_*(L)*, *v*_7_*(A)*, *v*_*19 *_*(NH_4_)*, *v*_*20*_*(Q) *and *v*_*22*_. The measured fluxes have a grey background and its band of uncertainty is represented with a black interval. The non-measured fluxes estimated with FSA are represented with a black interval, and the non-measured fluxes estimated with MFA with a green line. Two additional estimations with MFA are given at times 24 h and 168 h, where fluxes have been estimated from slight variations of the measured values for *v*_*1 *_and *v*_*6 *_(red x).

If the five measured fluxes are used (*v*_*1 *_(*G*), *v*_*6 *_(*L*),*v*_*7 *_(*A*), *v*_*19 *_(*NH*_*4*_) and *v*_*20 *_(*Q*)) and it is assumed that the formation of purine and pyrimidine nucleotides is the same (*v*_*22 *_= 0), the rank of *S*_*u *_(16) is equal to the number of unknown fluxes (22-5-1). Thereby the system (2) is determined but not redundant. In this case we could use MFA to determine the non-measured fluxes. More precisely, at each time instant *k*, the unique flux distribution fulfilling (2) can be obtained by using the inverse matrix of *S*_*m *_(see methods). However, as it can be observed in Figure 7 (green solid line) the results obtained are not very satisfactory:

• The estimated values at time 24 h an 168 h for fluxes *v*_*8*_, *v*_*9*_, *v*_*10*_, *v*_*11*_, *v*_*12 *_and *v*_*21 *_seem unreasonable: the measured fluxes evolve in a smooth way, but these fluxes show peak values.

• The estimated fluxes *v*_*8*_, *v*_*9 *_and *v*_*10 *_do not fulfil the reversibility constraints (they are not considered by MFA).

• MFA assumes that there is not any kind of error in the measurements, which is unlikely, and therefore the estimated fluxes are unreliable.

A new estimation has been done at time 24 h, where the measured values for fluxes *v*_*1 *_and *v*_*6 *_are slightly modified (+2% and -5% respectively). In a similar way, a new estimation at time 168 h assumes a slight variation of the measured values for *v*_*1 *_and *v*_*6 *_(-0.05 and +0.05 mM/(d·10^9^·cells), respectively). As it can be observed in Figure 7 (red crosses), the peak values in fluxes *v*_*8*_, *v*_*9*_, *v*_*10*_, *v*_*11*_, *v*_*12 *_and *v*_*21 *_are eliminated or reduced, while the values of the rest of non-measured fluxes remain almost unchanged. This demonstrates that the peak values at times 24 h and 168 h could be caused by slight errors in the measured fluxes. The same issue is illustrated with figure A1 (Additional File 7). Hence, the main weakness of MFA in the determined case is pointed out: the effect of slight errors in the measured fluxes is not under control. These slight errors will exist in virtually all the measured fluxes (none sensor has a precision of 100%). Moreover, even the conversion of the measured concentrations into measured fluxes may introduce slight errors. For this reason, the fluxes estimated with MFA are unreliable in this scenario.

The same scenario is now approached following the procedure introduced in this paper, i.e. using FSA instead of MFA in the third step. If uncertainty is not considered and all reactions are assumed to be reversible, FSA provides the same solution that MFA (results not shown). But it is possible to include the reversibility constraints for those reactions classified as irreversible. By using these constraints, FSA has detected a high inconsistency at 24 h and a lower one at 144 h (i.e. the region defined by the imposed constraints does not contain any solution at these time instants). It must be highlighted that the system is not redundant, so methods to check consistency based on redundancy cannot be used; however, FSA is detecting inconsistencies thanks to the reversibility constraints. Afterwards, it is also interesting to consider the intrinsic uncertainty of the measurements. We will define a band of uncertainty around the measured values, and then we will use FSA to estimate the non-measured fluxes. The most common ways to define a band of uncertainty are the use of a relative error around the measured values (e.g. of the 5%) and the use of an absolute one (e.g. 0.05 mM/(d•109•cells)). Herein, we use a mixed approach. For each measured flux *v*_*m*_, at each time instant *k*, the band is defined as:

If relErr⋅vm>absErr→band=vm±relErr⋅vmElse→band=vm±absErr
 MathType@MTEF@5@5@+=feaafiart1ev1aaatCvAUfKttLearuWrP9MDH5MBPbIqV92AaeXatLxBI9gBaebbnrfifHhDYfgasaacH8akY=wiFfYdH8Gipec8Eeeu0xXdbba9frFj0=OqFfea0dXdd9vqai=hGuQ8kuc9pgc9s8qqaq=dirpe0xb9q8qiLsFr0=vr0=vr0dc8meaabaqaciaacaGaaeqabaqabeGadaaakeaafaqaaeGadaaabaGaeeysaKKaeeOzayMaeeiiaaIaemOCaiNaemyzauMaemiBaWMaemyrauKaemOCaiNaemOCaiNaeyyXICTaemODay3aaSbaaSqaaiabd2gaTbqabaGccqGH+aGpcqWGHbqycqWGIbGycqWGZbWCcqWGfbqrcqWGYbGCcqWGYbGCaeaacqGHsgIRaeaacqWGIbGycqWGHbqycqWGUbGBcqWGKbazcqGH9aqpcqWG2bGDdaWgaaWcbaGaemyBa0gabeaakiabgglaXkabdkhaYjabdwgaLjabdYgaSjabdweafjabdkhaYjabdkhaYjabgwSixlabdAha2naaBaaaleaacqWGTbqBaeqaaaGcbaGaeeyrauKaeeiBaWMaee4CamNaeeyzaugabaGaeyOKH4kabaGaemOyaiMaemyyaeMaemOBa4MaemizaqMaeyypa0JaemODay3aaSbaaSqaaiabd2gaTbqabaGccqGHXcqScqWGHbqycqWGIbGycqWGZbWCcqWGfbqrcqWGYbGCcqWGYbGCaaaaaa@7B55@

With this expression the relative error (*relErr*) will be considered when the measured value is high, and the absolute one (*absErr*) when it is near to zero (see figure A2 in the Additional File 7). If more information about the measurements sources were available, the range of uncertainty of each measured flux could be defined accordingly. For example, if a commercial sensor is employed, its technical specifications can be used to define the band.

The non-measured fluxes estimated with FSA -when the band of uncertainty is considered and the reversibility constraints are incorporated- are shown in Figure 7 (black intervals). If they are compared with those obtained when MFA was used, several conclusions can be pointed out:

• The peaks at time 24 h an 168 h for fluxes *v*_*8*_, *v*_*9*_, *v*_*10*_, *v*_*11*_, *v*_*12 *_and *v*_*21 *_-which appeared when MFA was used- are avoided with FSA. As it was shown, when the measurements were slightly modified, these peak-values were replaced by more sensible predictions. Since these modified measurements are included in the band of uncertainty, the obtained intervals for *v*_*8*_, *v*_*9*_, *v*_*10*_, *v*_*11*_, *v*_*12 *_and *v*_*21 *_contain the sensible predictions. In principle, the peak-values would be within the intervals. However, a peak value could not satisfy the reversibility constraints and therefore it will not be considered a valid solution by FSA -this is the case at *k *= 24 h.

• The uncertainty of experimental measurements is non-trivially propagated to the non-measured fluxes. Hence, the use of FSA provides not only a prediction of the non-measured fluxes, but also an indication of the reliability of this prediction. For example, the predicted *v*_*8*_, *v*_*9 *_and *v*_*10 *_are highly influenced by measurements uncertainty, while *v*_*2*_, *v*_*4*_, and *v*_*5 *_are quite insensitive. Although all fluxes can be determined, FSA highlights that the estimated values for *v*_*8*_, *v*_*9 *_and *v*_*10 *_are less reliable (or less precise) than those assigned to *v*_*2*_, *v*_*4 *_and *v*_*5*_. This issue is more deeply analyzed in a subsequent section.

• Reversibility constraints provide a method to detect inconsistencies. For example, it can be easily checked that the solution provided by MFA do not satisfy the reversibility constraints at 24 h (a negative value is given to the irreversible fluxes *v*_*8*_, *v*_*9 *_and *v*_*10*_). This inconsistency is detected and avoided with FSA.

• The underdeterminancy introduced as uncertainty in the measurements can be partially neutralized with the reversibility constraints. Hence, the estimated fluxes are more reliable but not necessarily highly imprecise.

This example shows that the procedure provides a reliable and rich estimation of the evolution along time of the non-measured fluxes when the measurements are only *almost *sufficient, i.e. when the system is determined but not redundant. In particular, the use of FSA in the third step of the procedure -instead of the well-established MFA- provides several benefits, thanks to taking into account the uncertainty of measurements and considering the reversibility constraints.

#### Step 3 (S2): estimation of fluxes if measurements are sufficient and uncertain

When the system (2) is determined and redundant, an estimation based on MFA will work as follows (approach 1): firstly, the importance of the inconsistency is checked and the measured flux values are adjusted; then, the pseudo-inverse matrix is used to estimate the non-measured fluxes. These two properties -checkable consistency and adjustable measurements- are responsible of the success of MFA in this scenario. However, FSA provides a new approach (approach 2) which holds the property of checkable consistency, but replaces the adjustment of the measurements by the definition of a band of uncertainty. We will apply both alternatives to our example.

The system (2) was determined and not redundant when six fluxes were known. If another independent flux is measured, the system will be redundant because the rank of *S*_*u *_(15) will be less than *m *(16). Since no more fluxes were measured in [28], we will assume that the evolution of *v*_*21*_*(CO2) *has been measured -we chose it because it is a well-known extracellular flux. We assume that *v*_*21 *_evolves smoothly and that its values are within the intervals estimated with FSA in the previous section. Hence, at each time instant *k*, except 24 h and 168 h, the values given by MFA in the previous section are used as measured values (they lay within the intervals). The values at 24 h and 168 h are calculated by the approximation of a spline curve (see Figure A3 in the Additional File 7).

**Figure 8:**
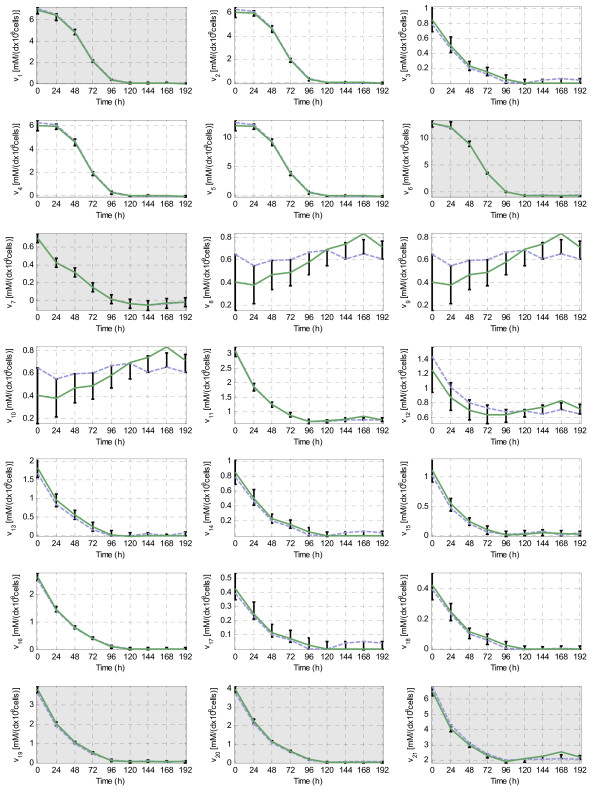
**FSA and MFA in the determined and redundant case (S2)**. The known fluxes are: *v*_*1*_*(G)*, *v*_*6*_*(L)*, *v*_*7*_*(A)*, *v*_*19*_*(NH*_*4*_), *v*_*20*_*(Q)*, *v*_*21*_*(CO2) *and *v*_*22*_. The measured fluxes have a grey background and its band of uncertainty is represented with a black interval. The non-measured fluxes estimated by FSA are denoted with a black interval, and the non-measured fluxes estimated with MFA with a green line. One consistent flux distribution within the intervals given by FSA has been highlighted (blue dotted line) to show its discrepancy with the one calculated with MFA. Notice that this flux distribution corresponds to a set of measurements very close to the original ones (± 5% or ± 0.05 mM/(d•10^9^•cells)).

First of all, we apply the χ-square method to estimate the importance of the inconsistency at each time instant *k *(see methods). The data fails the consistency check at time 168 h, what indicates that the set of measurements contains gross errors at this point (see table A1 in the Additional File 7). Afterwards, we estimate the non-measured fluxes at each time instant *k *with the two approaches described above. In the first one, the measured values are adjusted to be consistent (as explained in methods). Then, the non-measured fluxes are estimated with MFA. In the second one, a band of uncertainty around the measured values is defined trying to enclose some nearby consistent sets of measured fluxes (the band is the same that in the previous section). Then, the non-measured fluxes are estimated with FSA. The results (shown in Figure 8) illustrate the benefits of using FSA in this scenario:

• All the consistent sets of measured values enclosed by the band of uncertainty are considered by FSA. That guarantees that the intervals obtained enclose the actual values of the fluxes if the band was correctly chosen. Contrarily, when MFA is used (approach 1), the actual values of the measured fluxes need to be *exactly *found to ensure that the estimations fit in with the actual fluxes. To illustrate this idea a consistent flux distribution within the band of uncertainty has been highlighted in Figure 8 (dotted line). This flux distribution corresponds to a set of measured values very near to the original ones; nevertheless the evolution of *v*_*8*_, *v*_*9*_, *v*_*10 *_and *v*_*12 *_is quite different to the estimation given by MFA. That proves that the values estimated with MFA may be deviated from the actual ones, even if there are only slight errors in the measured fluxes. Conversely, FSA shows that two qualitatively different interpretations of fluxes *v*_*8*_, *v*_*9 *_and *v*_*10 *_are possible: they can be stable around 0.6 or evolve from 0.2 to 0.7 mM/(d * 10^9 ^cells). If there were other evidences supporting one alternative over the other one, we could hypothesize which of these two scenarios corresponds to the actual one. Hence, FSA not only reduces the number of wrong predictions, but may also provide a quantitative support for our qualitative knowledge.

• Although there is a gross error in the measurements at 168 h, FSA finds at least one consistent set of measured values within the band of uncertainty (providing an error bound that complements the χ-square method). The estimations provided by FSA at 168 h seem sensible: the measurements are only slightly adjusted (the adjustment is limited by the band size) and the peak values are avoided. On the contrary, the fluxes estimated with MFA are very sensitive to the gross error. The value of *v*_*21 *_is significantly changed by the adjustment method resulting in a peak. Moreover, this insensible peak also appears in the estimated values of *v*_*8*_, *v*_*9*_, *v*_*10*_, *v*_*11 *_and *v*_*12*_. In fact, the fluxes calculated with MFA are generally discarded when the measurements fail the χ-square method.

• When FSA is used, the uncertainty of experimental measurements is non-trivially translated to the non-measured fluxes. Again, FSA provides not only a prediction of the non-measured fluxes, but also an indication of the reliability of this prediction.

This example has shown that the procedure can be useful to estimate the evolution of the fluxes even when measurements are sufficient but uncertain, i.e. when the system is determined and redundant. Although this is the scenarios were the procedures based on the use of MFA are most successful, the use of FSA provides a more reliable estimation of the non-measured fluxes and offers an interesting approach to cope with inconsistency.

#### Step 3 (S3): estimation of fluxes if measurements are insufficient and uncertain

**Figure 9:**
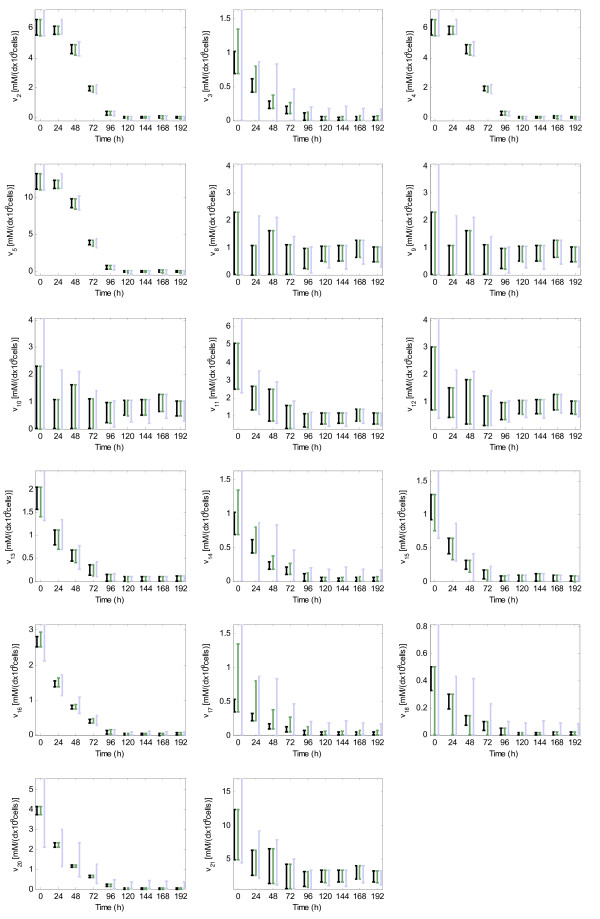
**Non-measured fluxes estimated with FSA in two underdetermined cases (S3)**. The estimations when five fluxes are measured (*v*_*1*_, *v*_*6*_, *v*_*7*_, *v*_*19 *_and *v*_*20*_) are depicted in green (second interval). The estimations when four fluxes are measured (*v*_*1*_, *v*_*6*_, *v*_*7 *_and *v*_19_) are depicted in blue (third interval). The estimations obtained in the determined case (when six fluxes were measured) are included for the shake of comparison (in black).

Finally, it will be shown that our procedure can be used even when the available measurements are insufficient (i.e. when system (2) is underdetermined). In this situation procedures based on MFA cannot be applied, but the use of FSA allows our procedure to estimate the interval of possible values for each non-measured flux. In particular, the non-measured fluxes will be estimated by using different sets of 5 and 4 measured fluxes -remember that 6 were necessary to get a determined system. In all cases, uncertainty has been considered using the band described above. All results are given in Table 2 and two illustrative cases are depicted in Figure 9.

**Table 2:** Comparison of different estimations of the non-measured fluxes

**Ref (v_**1**_, v_**6**_, v_**7**_, v_**19**_, v_**20**_, v_**22**_)**		**G (no v_**22**_)**		**F (no v_**20**_)**		**E (no v_**19**_)**		**B (no v_**6**_)**		**A (no v_**1**_)**		**C (no v_**7**_)**		**I (no v_**20 **_v_**22**_)**		**H (no v_**19 **_v_**22**_)**	
								
*Reactions *^*d*^	MI^a ^[^b^]	MI [^a^]	[%^c^]	MI [^b^]	[%]	MI [^b^]	[%]	MI [^b^]	[%]	MI [^b^]	[%]	MI [^b^]	[%]	MI [^b^]	[%]	MI [^b^]	[%]
*1: G→G6P*	0.267^*e*^	0.267	-	0.267	-	0.267	-	0.267	-	x	-	0.267	-	0.267	-	0.267	-
*2: G6P→G3P+DAP*	0.367	0.387	5%	0.628	71%	0.541	47%	0.398	8%	x	-	0.627	71%	0.628	71%	0.572	56%
*3: G6P→R5P+CO2*	0.131	0.199	53%	0.526	303%	0.340	160%	0.131	0%	0.131	0%	0.401	207%	0.526	303%	0.383	193%
*4: DAP→G3P*	0.367	0.387	5%	0.628	71%	0.541	47%	0.398	8%	x	-	0.627	71%	0.628	71%	0.572	56%
*5: G3P→Pyr*	0.735	0.774	5%	1.256	71%	1.082	47%	0.795	8%	x	-	1.253	71%	1.256	71%	1.144	56%
*6: Pyr→L*	0.475	0.475	-	0.475	-	0.475	-	x	-	0.475	-	0.475	-	0.475	-	0.475	-
*7: Pyr+Glu→A+aKG*	0.100	0.100	-	0.100	-	0.100	-	0.100	-	0.100	-	1.488	inf	0.100	-	0.100	-
*8: Pyr→ACA+CO2*	1.031	1.031	0%	1.562	51%	1.901	84%	x	-	x	-	0.957	-7%	1.562	51%	1.906	85%
*9: Oxa+ACA→Cit*	1.031	1.031	0%	1.562	51%	1.901	84%	x	-	x	-	0.957	-7%	1.562	51%	1.906	85%
*10: Cit→aKG+CO2*	1.031	1.031	0%	1.562	51%	1.901	84%	x	-	x	-	0.957	-7%	1.562	51%	1.906	85%
*11: aKG→Mal+CO2*	1.156	1.156	0%	1.604	39%	2.532	119%	x	-	x	-	1.443	25%	1.604	39%	2.530	119%
*12: Mal→Oxa*	0.994	0.994	0%	1.398	41%	1.769	78%	x	-	x	-	1.093	10%	1.398	41%	1.769	78%
*13: Mal→Pyr+CO2*	0.209	0.240	15%	0.352	68%	0.920	341%	0.209	0%	0.209	0%	0.903	332%	0.352	68%	0.918	340%
*14: Oxa+Glu→Asp+aKG*	0.131	0.199	53%	0.526	303%	0.340	160%	0.131	0%	0.131	0%	0.401	207%	0.526	303%	0.383	193%
*15: Glu→aKG+NH4*	0.150	0.182	21%	0.298	98%	0.870	479%	0.150	0%	0.150	0%	0.586	289%	0.298	98%	0.870	479%
*16: Q→Glu+NH4*	0.117	0.145	23%	0.325	177%	0.553	372%	0.117	0%	0.117	0%	0.569	386%	0.325	177%	0.548	367%
*17: R5P+Asp+Q→Pu*	0.104	0.277	165%	0.293	181%	0.200	91%	0.104	0%	0.104	0%	0.225	116%	0.526	404%	0.383	267%
*18: R5P+Asp+2Q→Py*	0.078	0.132	69%	0.283	262%	0.177	126%	0.078	0%	0.078	0%	0.209	168%	0.263	237%	0.163	108%
*19:→NH4*	0.141	0.141	-	0.141	-	1.419	904%	0.141	-	0.141	-	0.141	-	0.141	-	1.412	899%
*20:→Q*	0.132	0.132	-	1.127	752%	0.132	-	0.132	-	0.132	-	0.132	-	1.107	737%	0.132	-
*21:→CO2*	3.338	3.338	0%	4.770	43%	6.966	109%	x	-	x	-	3.843	15%	4.770	43%	6.966	109%
*22: Pu-Py (constraint)*	0.100	0.354	254%	0.100	-	0.100	-	0.100	-	0.100	-	0.100	-	0.526	426%	0.383	283%
																	
*Mean*	0.554	0.587	39%	0.899	155%	1.138	196%	0.217	2%	0.156	0%	0.802	122%	0.927	180%	1.168	214%

*Measured fluxes [number]*	6	5		5		5		5		5		5		4		4	
*Estimated fluxes [number]*	16/16	17/17		17/17		17/17		7/17		10/17		17/17		18/18		18/18	
*<25% (·Ref)*			12		0		0		10		7		5		0		0
*25–100% (<2·Ref)*			3		11		8		0		0		4		11		7
*100–300% (2–4·Ref)*			2		3		5		0		0		5		2		7
*>300% (>4·Ref)*			0		3		4		0		0		3		5		4

Column Ref: FSA is applied by using the six available measurements (Determined case). Columns F, G, E B, A and C: FSA is applied by using a different set of 5 measurements in each case (underdetermined, 1 degree of freedom). Columns I and H: FSA is applied by using two different sets of 4 measurements (underdetermined, 2 degrees of freedom). In all cases the band of uncertainty described in the text has been used. ^a ^Mean interval size along time evolution; ^b ^in [mM/(d * 10^9 cells)]; ^c ^Intervals enlargement w.r.t. case Ref. (in percentage); ^d ^The nomenclature is given in the additional file 1; ^e ^Measured values are in bold.

With four sets of 5 measurements (G, F, E and C) the evolution over time of all the non-measured fluxes can be estimated. Case G, where *v*_*22 *_is not known, provides the best results. There is a mean interval increment of 39% with respect to the determined case and the increment is minor than 25% for 12 fluxes (out of 17). This case is depicted in Figure 9 (in green). The intervals are practically the same than in the determined case for most fluxes (*v*_*2*_, *v*_*4*_, *v*_*5*_, *v*_*8*_, *v*_*9*_, *v*_*10*_, *v*_*11*_, *v*_*12*_, *v*_13_, *v*_*15 *_and *v*_*21*_). Intervals for *v*_*3 *_and *v*_*14 *_are larger, but still accurate, and only the estimations of *v*_*16*_, *v*_*17 *_and *v*_*18 *_are highly imprecise. Moreover, the temporal evolution -that can be characterized by using the middle point of the interval- is almost the same than of the determined case even for these fluxes (see figure A4 in the Additional File 7). Case C, where *v*_*7 *_is not measured, provides very good results. All fluxes are predicted (with a mean interval increment of 122%), the interval increment is minor than 25% for 5 fluxes and minor than 100% for 9 fluxes. Case F, where *v*_*20 *_is not measured, provides good results too. There is a mean interval increment of 155% and the interval increment is minor than 100% for 11 fluxes (out 17). Case E, where *v*_*19 *_is not measured, provides slightly worse results than F. With the other sets of 5 measurements (B and A), some non-measured fluxes cannot be estimated. Nevertheless the intervals of the fluxes that can be estimated (10 and 7 fluxes respectively) are exactly the same that in the determined case.

Two sets of 4 measurements have been studied (I and H). Case I, where *v*_*20 *_and *v*_*22 *_are not measured, provides remarkable results. There is a mean interval increment of 180% with respect to the determined case and the increment is minor than 100% for 11 fluxes (out 18). This case is depicted in Figure 9 (in blue). For most fluxes the intervals are similar to the determined case (*v*_*2*_, *v*_*4*_, *v*_*5*_, *v*_*8*_, *v*_*9*_, *v*_*10*_, *v*_*11*_, *v*_*12*_, *v*_*13*_, *v*_*15 *_and *v*_*21*_). Intervals for *v*_*16 *_and *v*_*20 *_are larger but still useful, and only *v*_*3*_, *v*_*14*_, *v*_*17 *_and *v*_*18 *_are highly imprecise. Again, the temporal evolution of the estimated fluxes is similar to the determined case (see figure A4 in the Additional File 7).

This scenario has illustrated an important feature of the introduced procedure: it can estimate the evolution of the non-measured fluxes even when there is a lack of measurable species (i.e. the system is underdetermined) and the available measurements are uncertain.

### Unbalanced propagation of measurements uncertainty

As it has been shown in previous sections, the uncertainty of the experimentally measured fluxes is not equally propagated to the estimated fluxes (i.e. not all the estimated fluxes are equally affected by measurements uncertainty). On the contrary, the structure of the metabolic network (the stoichiometric relations and the reversibility constraints) will determine how the uncertainty is propagated from the measured fluxes to the estimated ones.

A convenient way of measuring this effect is to calculate the interval size for each estimated flux at each time instant -in absolute and relative terms. The complete dataset has been included in the Additional File 6, but, as a summary, the average interval size (AIS) for each estimated flux is given in Table 3. It can be observed (determined case) that certain fluxes -such as *v*_*10*_, *v*_*12 *_and *v*_*21*_- are highly affected by the uncertainty of the measurements (they have an average interval size larger than 1 mM/(d·10^9^·cells)), while other fluxes -such as *v*_*14 *_and *v*_*17*_-are less sensitive (values around 0.1 mM/(d·10^9^·cells)). Although it is obvious that in relative terms the smaller fluxes are usually more affected by the uncertainty, this phenomenon is not the only responsible for the unbalanced propagation of the uncertainty. For example, the calculated fluxes *v*_*8 *_and *v*_*14 *_have a similar maximum value (around 1 mM/(d·10^9^·cells)), but the effect of the uncertainty over them is dramatically different: *v*_*8 *_is the flux more influenced by the uncertainty (with an AIS of 90.3% in relative terms) whereas *v*_*14 *_is quite insensitive to it (an AIS of 15.12%). Another example is given by *v*_*21*_: although being one of the fluxes with a bigger maximum value (8.6 mM/(d·10^9^·cells)), it is highly affected by the uncertainty (an AIS interval size of 3.4 mM/(d·10^9^·cells), which represents a 39.1%).

**Table 3:** Imprecision of the estimated fluxes caused by measurements uncertainty

	**Determined case**			**Determined/Redundant case**			**Comparative**	
	*Max [*^*a*^]	*AIS [*^*a*^]	*AIS [%*^*b*^]	*Max [*^*a*^]	*AIS [*^*a*^]	*AIS [%*^*b*^]	*Diff. [*^*a*^]	*Diff. [%]*
	
**v**_**2**_	6,041	0,377	6,25%	6,032	0,321	5,32%	0,057	14,97%
**v**_**3**_	0,853	0,129	15,12%	0,859	0,123	14,35%	0,006	4,41%
**v**_**4**_	6,041	0,377	6,25%	6,032	0,321	5,32%	0,057	14,97%
**v**_**5**_	12,081	0,755	6,25%	12,065	0,642	5,32%	0,113	14,98%
**v**_**8**_	1,166	1,053	90,37%	0,715	0,231	32,32%	0,822	78,07%
**v**_**9**_	1,166	1,053	90,37%	0,715	0,231	32,32%	0,822	78,07%
**v**_**10**_	1,166	1,053	90,37%	0,715	0,231	32,32%	0,822	78,07%
**v**_**11**_	3,769	1,180	31,30%	3,073	0,165	5,37%	1,015	86,02%
**v**_**12**_	1,854	1,017	54,89%	1,263	0,241	19,05%	0,777	76,34%
**v**_**13**_	1,813	0,209	11,52%	1,809	0,195	10,78%	0,014	6,58%
**v**_**14**_	0,853	0,129	15,12%	0,859	0,123	14,35%	0,006	4,41%
**v**_**15**_	1,113	0,150	13,52%	1,109	0,147	13,27%	0,003	2,11%
**v**_**16**_	2,665	0,117	4,39%	2,668	0,114	4,26%	0,003	2,91%
**v**_**17**_	0,426	0,101	23,64%	0,442	0,087	19,60%	0,014	14,10%
**v**_**18**_	0,426	0,079	18,42%	0,417	0,063	15,17%	0,015	19,48%
**v**_**21**_	8,698	3,407	39,17%		-	-	-	-
								
**Mean**		0,699	32,31%		0,202	14,32%	0,497	71,09%

Max: Maximum value of the estimated flux along time; AIS: Averaged interval size for each estimated fluxes (average of its interval sizes along time); Diff: Difference between determined and overdetermined cases; ^a ^in [mM/(d × 109 × cells)]; ^b ^the interval size for each estimated flux is expressed w.r.t. its maximum value. The complete dataset is given in the additional file 6.

Furthermore, the data given in Table 3 provides a quantitative indication of the benefits of incorporating a redundant measurement. When seven fluxes are assumed to be measurable instead of six, the estimations of the non-measured fluxes are more precise (the interval sizes are reduced around 71% on average). This is particularly important for those fluxes that were poorly estimated in the determined case (reductions of 78% for *v*_*8*_, *v*_*9 *_and *v*_*10 *_and 76% for *v*_*12*_).

### Non-linear propagation of measurements uncertainty

In the previous section we analyzed the unbalanced propagation of the uncertainty from the measured fluxes to the estimated ones. Herein we investigate some characteristics of this propagation and, in particular, the interrelation between the uncertainty of the different measured fluxes and their combined effect over the estimated fluxes.

Again, the time series of the five measured species (*G*, *L*, *A*, *NH*_*4 *_and *Q*) have been used, under the assumption that the formation of purine and pyrimidine are equal (*v*_*22 *_= 0). Then, 15·15 executions of the estimation procedure have been carried out with different degrees of uncertainty for the measured fluxes *v*_*1 *_and *v*_*6 *_(between ± 2% and ± 30%). Afterwards, the averaged interval size for each estimated flux was calculated. This makes it possible to analyze how the different combinations of uncertainty in *v*_*1 *_and *v*_*6 *_affect to the estimated fluxes.

**Figure 10:**
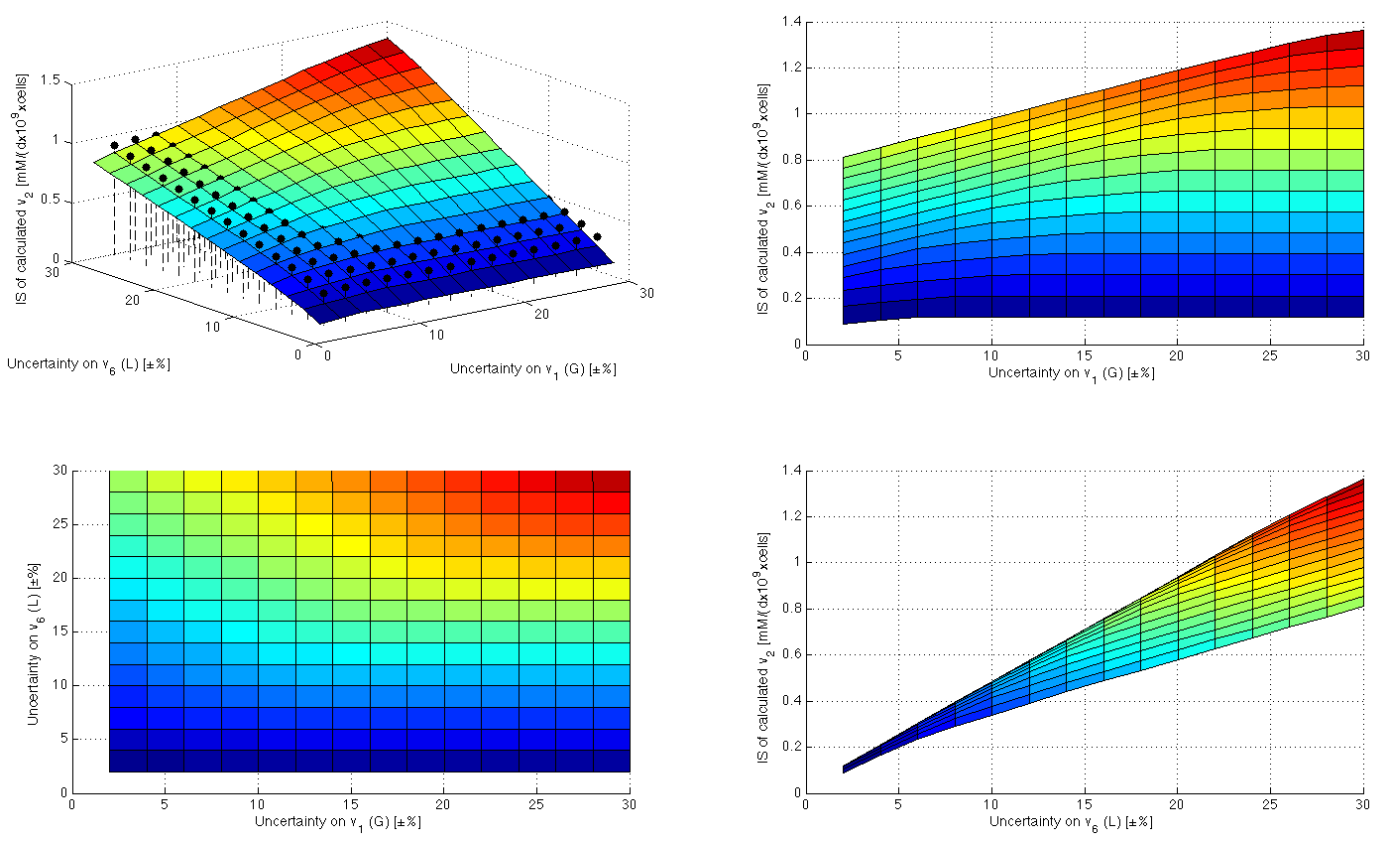
**Effect over the estimated flux *v*_*2 *_of the uncertainty on the measured values of *v*_*1 *_and *v*_*6*_**. The surface (and its projections) represents the averaged interval size (IS) of the estimated *v*_*2 *_when different degrees of uncertainty are considered for the measured fluxes *v*_*1 *_and *v*_*6*_. In the top left figure the results of summing up the independents effects of *v*_*6 *_uncertainty and *v*_*1 *_uncertainty are depicted with black dots.

Figure 10 shows the averaged interval size (AIS) of one of the estimated fluxes (*v*_*2*_) for each execution (similar figures are given in the Additional File 7). As it was predictable, the interval size tends to increase as the uncertainty of the measurements is increased. Therefore, the less precise estimation (i.e. the biggest AIS) corresponds to the execution with maximum uncertainty for *v*_*1 *_and *v*_*6*_. It is also seen that, as it was expected, the uncertainty of all the measured fluxes has not the same effect over the estimated ones. For instance, the uncertainty of *v*_*6 *_has a bigger effect over *v*_*2 *_than the uncertainty of *v*_*1*_. More even, the figures illustrate two important properties of the propagation of the measurements uncertainty to the estimated fluxes.

On the one hand, *the propagation of the uncertainty does not satisfy the principle of superposition*. Let *f*(*u*_*i*_) be the interval size of a calculated flux when the degree of measurements uncertainty is *u*_*i*_, then: *f *(*u*_1_) + *f *(*u*_2_) ≠ *f *(*u*_1 _+ *u*_2_). To remark this, the result of summing up the independent effect of the uncertainty of *v*_*6 *_and *v*_*1 *_has been depicted in Figure 10 (black dots). Interestingly, if the uncertainty of one of the two measured fluxes is kept low then *f *(*u*_1_) + *f *(*u*_2_) > *f *(*u*_1 _+ *u*_2_); but if the uncertainty of both fluxes is increased, this is inverted: *f *(*u*_1_) + *f *(*u*_2_) <*f *(*u*_1 _+ *u*_2_). This implies that the net result of considering the uncertainty of two (or more) measured fluxes can not be predicted just by summing up the results of considering only the uncertainty of one measured flux at a time. On the contrary, the net result may be given by a complex non-linear function, as it happens in the example of Figure 10, where:

• The uncertainty in *v*_*6 *_is always translated to the estimated *v*_*2 *_(see right bottom figure). When *v*_*6 *_uncertainty increases, the AIS of *v*_*2 *_increases -even if there is not *v*_*1 *_uncertainty. However, the bigger *v*_*1 *_uncertainty gets, the bigger the effect of adding *v*_*6 *_uncertainty is.

• When *v*_*6 *_uncertainty is low, the addition of *v*_*1 *_uncertainty has a low effect over the estimated *v*_*2 *_(see right bottom figure) More precisely, the first small addition of *v*_*1 *_uncertainty has a slight effect, but the subsequent additions do not (there is a saturation). However, the saturation limit increases with *v*_*6 *_uncertainty, and, therefore, the more uncertainty there is in *v*_*6*_, the more important effect has addition of uncertainty in *v*_*1*_. In summary, *v*_*1 *_uncertainty has not an important effect itself, but its combination with *v*_*6 *_uncertainty boosts it.

On the other hand, as the superposition principle is not fulfilled *the effect of the uncertainty of one measured flux over one estimated flux is not linear*, i.e. *f *(*k*·*u*_1_) ≠ *k*·*f *(*u*_1_) For example, assume that the uncertainty of *v*_*6 *_is fixed in 10% (fourth row in the right top figure). It can be observed that the effect of adding a first 4% of uncertainty to *v*_*1 *_is higher than the effect of adding a second one. In fact, when the uncertainty of *v*_*1 *_is bigger than 16%, the addition of more uncertainty has practically zero effect (there is a saturation phenomenon).

In the last two sections it has been shown that the relationship between the uncertainty of the measurements and the precision of the estimation is a complex one. On the one hand, the propagation of measurements uncertainty to each estimated flux will be different. On the other hand, the net effect of considering the uncertainty of two (or more) measured fluxes simultaneously does not correspond to the sum of the effects of considering the uncertainty of each measured flux one at a time. Finally, the effect of the uncertainty of one measured flux over one estimated flux is not linear.

Therefore, when the procedure introduced in this paper considers the propagation of the uncertainty from the measurements to the estimated fluxes, it provides non-trivial information.

### Analysis of the effect of the uncertainty of each measured flux

In this section we analyze the effect of the uncertainty of each measured flux over the imprecision of the estimated fluxes. Basically, we can apply the estimation procedure over previously logged data -considering the uncertainty of each measured flux one at a time- in order to determine which measured fluxes have the more critical uncertainty. There are two similar approaches to carry out the analysis:

a) Direct approach. Calculate the *increase *of the imprecision of the estimated fluxes when the uncertainty of one measured flux is *increased*. This calculation is repeated for each measured flux.

b) Indirect approach. Calculate the *reduction *of the imprecision of the estimated fluxes when the uncertainty of one measured flux is *decreased*. This is repeated for each measured flux. Notice that the effect of decreasing the uncertainty is not the inverse of increasing it, i.e. *f *(*u *+ *x*).+ *f *(*u *+ *x*) ≠ *f *(0).

The direct approach (a) informs about the effect of considering the uncertainty of each measured flux over the estimated ones (it is similar to a classical analysis of sensitivity). This information may be useful during the setting-up of a process plant in order to choose the sources of measurements (the equipment and the protocols). Nevertheless, the indirect approach (b) is probably more promising. It calculates how much the imprecision of the estimated fluxes will be reduced, if we reduce the uncertainty of one of the measured fluxes. Given the characteristics of our current equipment and our measuring protocols (e.g. our sensors provide measurements with a ± 5% of uncertainty), we can calculate which of the measured fluxes should be more accurately measured in order to improve the precision of the estimations (e.g. using a more accurate sensor or taking redundant measurements).

**Figure 11:**
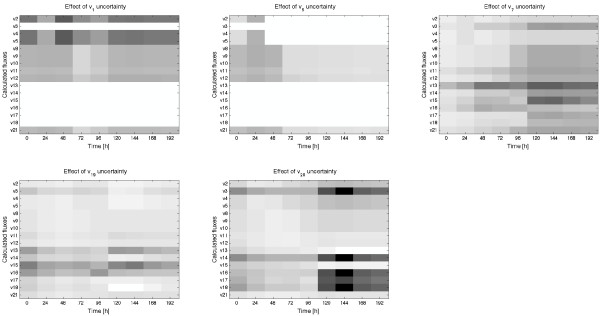
**Effect of the uncertainty of each measured flux over the imprecision of the estimated fluxes along time**. The figures show the reduction of the imprecision of the estimated (or calculated) fluxes when the uncertainty of the measured flux is decreased a 3%. The reductions are quantified between 0% (white colour) and 100% (black).

**Figure 12:**
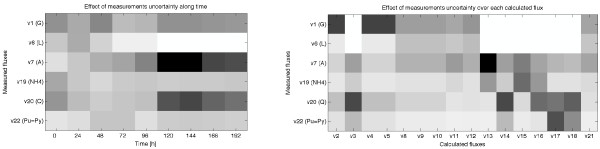
**Summary of the effect of the uncertainty of each measured flux over the imprecision of the estimated fluxes**. (Left) Averaged reduction at each time instant of the imprecision of the estimated (or calculated) fluxes when the uncertainty of the measured flux is decreased a 3% (Right) Averaged reduction of the imprecision of each estimated (or calculated) flux when the uncertainty of the measured flux is decreased a 3%. The reductions are quantified between 0% (white colour) and 100% (black).

The indirect analysis has been applied to the cultivation of CHO cells (using the set of 5 measurements described above and the assumption of equal formation of purine and pyrimidine). Figure 11 shows the reduction of the imprecision of each estimated flux when the uncertainty of a measured flux is decreased a 3%. This is repeated for each measured flux (*v*_*1*_, *v*_*6*_, *v*_*7*_, *v*_*19 *_and *v*_*20*_). Those data provide valuable information. For example, it is shown that the maximum reduction of the imprecision occurs when the uncertainty of *v*_*20 *_is reduced at time 144 h: the imprecision of *v*_*3*_, *v*_*14*_, *v*_*16 *_and *v*_*18 *_is reduced more than 85%. It can be also observed that during the first 96 h removing the uncertainty of *v*_*20 *_slightly reduces the imprecision of the estimated *v*_*16*_, but this reduction is very important between 120 h and 192 h. Those data are summarized in Figure 12. The left figure shows the averaged reduction at each time instant, and the one on the right the averaged reduction for each estimated flux. For example, it can be observed that removing the uncertainty of *v*_*1 *_or *v*_*6 *_has no effect over the estimations of *v*_*3*_, *v*_*14*_, *v*_*15*_, *v*_*16 *_*v*_*17 *_and *v*_*18*_. This information can be used to improve our estimations in a rational manner. For example, one could be interested in increasing the precision of the estimation of *v*_*3*_. In this case the indirect analysis indicates that the best option is to reduce the uncertainty of the measured *v*_*20*_. However, if we want to improve the estimations during the transition phase (between 72 h and 120 h) we should reduce the uncertainty of *v*_*7*_. Finally, if we prefer to improve the overall precision of the estimations we should reduce the uncertainty of *v*_*7*_, although reducing the uncertainty of *v*_*1 *_or *v*_*20 *_brings similar benefits.

More details about this analysis -including the complete dataset- are given in the Additional File 8. In addition, the results obtained with the direct analysis are also included there.

## Conclusion

In this contribution we have presented a new procedure to estimate the temporal evolution of the metabolic fluxes. It copes with the intrinsic uncertainty of experimental measurements and with the lack of measurable species by means of the use of the Flux Spectrum Approach (FSA). The potential of the procedure has been demonstrated using a real problem: the estimation of the intracellular fluxes of CHO cells cultivated in batch mode in stirred flasks. Using this example, the benefits that the use of FSA brings to the whole procedure have been illustrated through a comparison with the use of Metabolic Flux Analysis (MFA), a well-established methodology that is the basis of related procedures [24-26]. When the available measurements are only *almost *sufficient (i.e. the system is determined but not redundant), the procedure provides a more reliable and richer estimation of the evolution of the fluxes, because it takes into account measurements uncertainty and it considers the reversibility constraints. It has been also shown that even when measurements are insufficient (i.e. the system is underdetermined), the procedure is capable of estimating the evolution of the non-measured fluxes. Finally, when measurements are sufficient (i.e. the system is determined and redundant), the procedure provides a reliable estimation of the non-measured fluxes -because it considers measurements uncertainty- and offers an interesting approach to cope with inconsistency.

The procedure to estimate the metabolic fluxes can be applied off-line (with previously collected data), providing an insight into the time-varying behaviour of the organism. This can help in the understanding of its dynamic metabolic regulation and its adaptation to the environmental conditions. It can also be useful for physiological studies, strain characterization tasks, and to guide research to improve strain and processes. On the other hand, the procedure is a promising tool for on-line monitoring processes in industrial environments, where there is still a lack of reliable on-line sensors. The features of the procedure are especially suitable for this application.

In summary, it has been shown that the temporal evolution of non-measured fluxes can be estimated by using a set of measurable species and a set of known biological constraints. Moreover, the procedure proposed considers the intrinsic uncertainty of the experimental measurements and could be applied even if there is a lack of measurable species.

## Methods

### Calculation of the measured fluxes by approximating the derivative

If the concentration of an extracellular specie is measured, the value of its corresponding flux can be worked out from eq. 3. But that means the derivative *dξ/dt *has to be approximated. The Euler methods provide the most straightforward approximations:

Backward:df(k)dt≈f(k)−f(k−1)t(k)−t(k−1)Middlepoint:df(k)dt≈f(k+1)−f(k−1)t(k+1)−t(k−1)
 MathType@MTEF@5@5@+=feaafiart1ev1aaatCvAUfKttLearuWrP9MDH5MBPbIqV92AaeXatLxBI9gBaebbnrfifHhDYfgasaacH8akY=wiFfYdH8Gipec8Eeeu0xXdbba9frFj0=OqFfea0dXdd9vqai=hGuQ8kuc9pgc9s8qqaq=dirpe0xb9q8qiLsFr0=vr0=vr0dc8meaabaqaciaacaGaaeqabaqabeGadaaakeaafaqaaeGacaaabaGaeeOqaiKaeeyyaeMaee4yamMaee4AaSMaee4DaCNaeeyyaeMaeeOCaiNaeeizaqMaeiOoaOdabaWaaSaaaeaacqWGKbazcqWGMbGzcqGGOaakcqWGRbWAcqGGPaqkaeaacqWGKbazcqWG0baDaaGaeyisIS7aaSaaaeaacqWGMbGzcqGGOaakcqWGRbWAcqGGPaqkcqGHsislcqWGMbGzcqGGOaakcqWGRbWAcqGHsislcqaIXaqmcqGGPaqkaeaacqWG0baDcqGGOaakcqWGRbWAcqGGPaqkcqGHsislcqWG0baDcqGGOaakcqWGRbWAcqGHsislcqaIXaqmcqGGPaqkaaaabaGaeeyta0KaeeyAaKMaeeizaqMaeeizaqMaeeiBaWMaeeyzauMaeeiCaaNaee4Ba8MaeeyAaKMaeeOBa4MaeeiDaqNaeiOoaOdabaWaaSaaaeaacqWGKbazcqWGMbGzcqGGOaakcqWGRbWAcqGGPaqkaeaacqWGKbazcqWG0baDaaGaeyisIS7aaSaaaeaacqWGMbGzcqGGOaakcqWGRbWAcqGHRaWkcqaIXaqmcqGGPaqkcqGHsislcqWGMbGzcqGGOaakcqWGRbWAcqGHsislcqaIXaqmcqGGPaqkaeaacqWG0baDcqGGOaakcqWGRbWAcqGHRaWkcqaIXaqmcqGGPaqkcqGHsislcqWG0baDcqGGOaakcqWGRbWAcqGHsislcqaIXaqmcqGGPaqkaaaaaaaa@8EE8@

The backward version does not introduce an intrinsic delay (the derivative of *f(.) *at *k *is calculated with the values of *f(.) *at *k *and *k-1*), but the middle point provides a less noisy approximation. In any case, usually this approach needs to be combined with the use of filters.

### Moving average filters

These filters calculate each value of a new signal by averaging the values of the original signal within a time window. Thus, the new signal becomes smoother. The centred moving average (CMA) provides the best results because it uses past and future information. The filtered value for instant *k *(*CMA*_*k*_) is calculated by averaging the values of the original signal (*S*) between *k-n *and *k+n*:

CMAk=∑1nSk−i+Sk+∑1nSk+i2⋅n+1
 MathType@MTEF@5@5@+=feaafiart1ev1aaatCvAUfKttLearuWrP9MDH5MBPbIqV92AaeXatLxBI9gBaebbnrfifHhDYfgasaacH8akY=wiFfYdH8Gipec8Eeeu0xXdbba9frFj0=OqFfea0dXdd9vqai=hGuQ8kuc9pgc9s8qqaq=dirpe0xb9q8qiLsFr0=vr0=vr0dc8meaabaqaciaacaGaaeqabaqabeGadaaakeaacqWGdbWqcqWGnbqtcqWGbbqqdaWgaaWcbaGaem4AaSgabeaakiabg2da9maalaaabaWaaabCaeaacqWGtbWudaWgaaWcbaGaem4AaSMaeyOeI0IaemyAaKgabeaakiabgUcaRiabdofatnaaBaaaleaacqWGRbWAaeqaaOGaey4kaSYaaabCaeaacqWGtbWudaWgaaWcbaGaem4AaSMaey4kaSIaemyAaKgabeaaaeaacqaIXaqmaeaacqWGUbGBa0GaeyyeIuoaaSqaaiabigdaXaqaaiabd6gaUbqdcqGHris5aaGcbaGaeGOmaiJaeyyXICTaemOBa4Maey4kaSIaeGymaedaaaaa@50BF@

If only past values of the original signal are available, the standard moving average (SMA) can be used instead:

SMAk=∑0nSk−in+1
 MathType@MTEF@5@5@+=feaafiart1ev1aaatCvAUfKttLearuWrP9MDH5MBPbIqV92AaeXatLxBI9gBaebbnrfifHhDYfgasaacH8akY=wiFfYdH8Gipec8Eeeu0xXdbba9frFj0=OqFfea0dXdd9vqai=hGuQ8kuc9pgc9s8qqaq=dirpe0xb9q8qiLsFr0=vr0=vr0dc8meaabaqaciaacaGaaeqabaqabeGadaaakeaacqWGtbWucqWGnbqtcqWGbbqqdaWgaaWcbaGaem4AaSgabeaakiabg2da9maalaaabaWaaabCaeaacqWGtbWudaWgaaWcbaGaem4AaSMaeyOeI0IaemyAaKgabeaaaeaacqaIWaamaeaacqWGUbGBa0GaeyyeIuoaaOqaaiabd6gaUjabgUcaRiabigdaXaaaaaa@3F81@

The key parameter of moving average filters is the size of the window (i.e., the number of averaged values of the original signal). The optimal size would be one observation in order to be as close as possible to the original signal. However, as noise rejection is desired, the window size needs to be increased. Hence, there is a trade-off between sensitivity to noise and delay with respect to the original signal. A typical variant of these filters includes multiplying factors to give a different weight to each value within the time window (e.g. the exponential moving average).

### Calculation of the measured fluxes with a nonlinear observer

A high-gain nonlinear observer of the extracellular fluxes can be directly synthesized from (3) by using the method proposed in [47]:

dξedt=ve⋅X−D⋅ξe−2⋅θ⋅(ξe−ξ)dvedt=−θ2⋅(ξe−ξ)X
 MathType@MTEF@5@5@+=feaafiart1ev1aaatCvAUfKttLearuWrP9MDH5MBPbIqV92AaeXatLxBI9gBaebbnrfifHhDYfgasaacH8akY=wiFfYdH8Gipec8Eeeu0xXdbba9frFj0=OqFfea0dXdd9vqai=hGuQ8kuc9pgc9s8qqaq=dirpe0xb9q8qiLsFr0=vr0=vr0dc8meaabaqaciaacaGaaeqabaqabeGadaaakeaafaqaaeGabaaabaWaaSaaaeaacqWGKbaziiGacqWF+oaEdaWgaaWcbaGaemyzaugabeaaaOqaaiabdsgaKjabdsha0baacqGH9aqpcqWG2bGDdaWgaaWcbaGaemyzaugabeaakiabgwSixlabdIfayjabgkHiTiabdseaejabgwSixlab=57a4naaBaaaleaacqWGLbqzaeqaaOGaeyOeI0IaeGOmaiJaeyyXICTae8hUdeNaeyyXICTaeiikaGIae8NVdG3aaSbaaSqaaiabdwgaLbqabaGccqGHsislcqWF+oaEcqGGPaqkaeaadaWcaaqaaiabdsgaKjabdAha2naaBaaaleaacqWGLbqzaeqaaaGcbaGaemizaqMaemiDaqhaaiabg2da9iabgkHiTmaalaaabaGae8hUde3aaWbaaSqabeaacqaIYaGmaaGccqGHflY1cqGGOaakcqWF+oaEdaWgaaWcbaGaemyzaugabeaakiabgkHiTiab=57a4jabcMcaPaqaaiabdIfaybaaaaaaaa@6A31@

where *ξ*_*e *_denotes the observed concentration of the extracellular specie and *v*_*e *_the observed flux. The unique adjustable parameter is *θ*. Not only these observers are proved to be stable, but also its asymptotic error can be made arbitrarily small by choosing sufficiently large values of *θ*. However, very large values need to be avoided in practice since the observer may become noise sensitive. Thereby the choice of *θ *represents a trade-off between fast convergence (minor delay) and sensitivity to noise.

### Flux spectrum approach

The non-measured fluxes at a certain time instant *k *can be estimated using the Flux Spectrum Approach (FSA). The method works as follows [33]:

1. Impose the set of constraints given by (2) and the reversibility constraints for the irreversible reactions, *v*_*i *_≥ 0. Then, in order to consider the uncertainty of the measured fluxes, the unique value of each measured flux *v*_*m *_can be replaced by an interval [*v*_*m*, *min*_, *v*_*m*, *max*_]. Accordingly, each equation of (2) is substituted by two inequalities. The resultant constraints define a region where the actual flux distribution may live.

2. Calculate the minimum and maximum values within the region for each non-measured flux *v*_*uj*_, by solving a set of min/max linear programming problems (*n*_*u *_minimizations and *n*_*u *_maximizations):

∀vuj,j=1,…,nuMin{vuj}subject:Su⋅vu≥{−Sm⋅[vm]}min⁡Su⋅vu≤{−Sm⋅[vm]}max⁡vi≥0Max{vuj}subject:Su⋅vu≥{−Sm⋅[vm]}min⁡Su⋅vu≤{−Sm⋅[vm]}max⁡vi≥0
 MathType@MTEF@5@5@+=feaafiart1ev1aaatCvAUfKttLearuWrP9MDH5MBPbIqV92AaeXatLxBI9gBaebbnrfifHhDYfgasaacH8akY=wiFfYdH8Gipec8Eeeu0xXdbba9frFj0=OqFfea0dXdd9vqai=hGuQ8kuc9pgc9s8qqaq=dirpe0xb9q8qiLsFr0=vr0=vr0dc8meaabaqaciaacaGaaeqabaqabeGadaaakqaabeqaaiabgcGiIiabdAha2naaBaaaleaacqWG1bqDcqWGQbGAaeqaaOGaeiilaWIaemOAaOMaeyypa0JaeGymaeJaeiilaWIaeSOjGSKaeiilaWIaemOBa42ccqWG1bqDaOqaauaabeqabiaaaeaacqWGnbqtcqWGPbqAcqWGUbGBcqGG7bWEcqWG2bGDdaWgaaWcbaGaemyDauNaemOAaOgabeaakiabc2ha9bqaaiabdohaZjabdwha1jabdkgaIjabdQgaQjabdwgaLjabdogaJjabdsha0jabcQda6aaaaeaafaqabeqaeaaaaeaaaeaacqWGtbWudaWgaaWcbaGaemyDauhabeaakiabgwSixlabhAha2naaBaaaleaacqWH1bqDaeqaaOGaeyyzIm7aaiWaaeaacqGHsislcqWGtbWudaWgaaWcbaGaemyBa0gabeaakiabgwSixlabcUfaBjabhAha2naaBaaaleaacqWHTbqBaeqaaOGaeiyxa0facaGL7bGaayzFaaWaaWbaaSqabeaacyGGTbqBcqGGPbqAcqGGUbGBaaaakeaacqWGtbWudaWgaaWcbaGaemyDauhabeaakiabgwSixlabhAha2naaBaaaleaacqWH1bqDaeqaaOGaeyizIm6aaiWaaeaacqGHsislcqWGtbWudaWgaaWcbaGaemyBa0gabeaakiabgwSixlabcUfaBjabhAha2naaBaaaleaacqWHTbqBaeqaaOGaeiyxa0facaGL7bGaayzFaaWaaWbaaSqabeaacyGGTbqBcqGGHbqycqGG4baEaaaakeaacqWG2bGDdaWgaaWcbaGaemyAaKgabeaakiabgwMiZkabicdaWaaaaeaafaqabeqacaaabaGaemyta0KaemyyaeMaemiEaGNaei4EaSNaemODay3aaSbaaSqaaiabdwha1jabdQgaQbqabaGccqGG9bqFaeaacqWGZbWCcqWG1bqDcqWGIbGycqWGQbGAcqWGLbqzcqWGJbWycqWG0baDcqGG6aGoaaaabaqbaeqabeabaaaabaaabaGaem4uam1aaSbaaSqaaiabdwha1bqabaGccqGHflY1cqWH2bGDdaWgaaWcbaGaeCyDauhabeaakiabgwMiZoaacmaabaGaeyOeI0Iaem4uam1aaSbaaSqaaiabd2gaTbqabaGccqGHflY1cqGGBbWwcqWH2bGDdaWgaaWcbaGaeCyBa0gabeaakiabc2faDbGaay5Eaiaaw2haamaaCaaaleqabaGagiyBa0MaeiyAaKMaeiOBa4gaaaGcbaGaem4uam1aaSbaaSqaaiabdwha1bqabaGccqGHflY1cqWH2bGDdaWgaaWcbaGaeCyDauhabeaakiabgsMiJoaacmaabaGaeyOeI0Iaem4uam1aaSbaaSqaaiabd2gaTbqabaGccqGHflY1cqGGBbWwcqWH2bGDdaWgaaWcbaGaeCyBa0gabeaakiabc2faDbGaay5Eaiaaw2haamaaCaaaleqabaGagiyBa0MaeiyyaeMaeiiEaGhaaaGcbaGaemODay3aaSbaaSqaaiabdMgaPbqabaGccqGHLjYScqaIWaamaaaaaaa@E443@

where *n*_*u *_is the number of unknown fluxes, **v**_**u **_is the vector of non-measured fluxes, *v*_i _represents each irreversible flux and [**v**_**m**_] represents the two vectors [**v**_**m, min**_, **v**_**m, max**_] formed with the uncertain representation of the measured fluxes. {-*S*_*m*_·[**v**_**m**_]}^min ^and {-*S*_*m*_·[**v**_**m**_]}^max ^are vectors formed with the maximum and minimum row values of the product -*S*_*m*_· [**v**_**m**_]. To calculate each row, maximum and minimum values of **v**_**m **_need to be combined taking into account the signs of the elements of the corresponding row of *S*_*m *_(you can find a detailed description of the algorithm in the Additional File 5).

The obtained *v*_*uj, min *_and *v*_*uj, max *_for each non-measured flux define an interval bracketing its possible values: *v*_*uj*_*(k) *= [*v*_*uj, min*_, *v*_*uj, max*_].

### Auxiliary: link between extracellular and intracellular fluxes

A set of *ne *extracellular fluxes can be linked with the intracellular fluxes by using a matrix *S*_*ξ *_fulfilling:

**v**_**ξ **_= *S*_*ξ*_·**v**

where **v**_**ξ **_is the vector of extracellular fluxes and **v **the vector of intracellular fluxes. Eq. 11 can be easily joined with eq. 1 as follows:

[Smxn0mxneSξnexnInexne]⋅[vvξ]=[00]
 MathType@MTEF@5@5@+=feaafiart1ev1aaatCvAUfKttLearuWrP9MDH5MBPbIqV92AaeXatLxBI9gBaebbnrfifHhDYfgasaacH8akY=wiFfYdH8Gipec8Eeeu0xXdbba9frFj0=OqFfea0dXdd9vqai=hGuQ8kuc9pgc9s8qqaq=dirpe0xb9q8qiLsFr0=vr0=vr0dc8meaabaqaciaacaGaaeqabaqabeGadaaakeaalmaadmaajugqbeaafaqabeGacaaabaGaem4uam1cdaWgaaqcLbuabaWccqWGTbqBcqWG4baEcqWGUbGBaKqzafqabaaabaGaeGimaaZcdaWgaaqcLbuabaWccqWGTbqBcqWG4baEcqWGUbGBcqWGLbqzaKqzafqabaaabaGaem4uamfcciWccqWF+oaEdaWgaaqcLbuabaWccqWGUbGBcqWGLbqzcqWG4baEcqWGUbGBaKqzafqabaaabaGaemysaK0cdaWgaaqcLbuabaWccqWGUbGBcqWGLbqzcqWG4baEcqWGUbGBcqWGLbqzaKqzafqabaaaaaGccaGLBbGaayzxaaqcLbuacqGHflY1lmaadmaajugqbeaafaqabeGabaaabaGaeCODayhabaGaeCODay3cdaWgaaqaaiab=57a4bqabaaaaaGccaGLBbGaayzxaaqcLbuacqGH9aqplmaadmaajugqbeaafaqabeGabaaabaGaeGimaadabaGaeGimaadaaaGccaGLBbGaayzxaaaaaa@6337@

Hence, the extended system holds the structure of a homogeneous system of linear equations.

### Auxiliary: inconsistency of the measured fluxes

A redundant system will be consistent if it fulfils the consistency condition:

R⋅vm=0R=Sm−Su⋅Su#⋅Sm
 MathType@MTEF@5@5@+=feaafiart1ev1aaatCvAUfKttLearuWrP9MDH5MBPbIqV92AaeXatLxBI9gBaebbnrfifHhDYfgasaacH8akY=wiFfYdH8Gipec8Eeeu0xXdbba9frFj0=OqFfea0dXdd9vqai=hGuQ8kuc9pgc9s8qqaq=dirpe0xb9q8qiLsFr0=vr0=vr0dc8meaabaqaciaacaGaaeqabaqabeGadaaakeaafaqabeqacaaabaGaemOuaiLaeyyXICTaeCODay3aaSbaaSqaaiabh2gaTbqabaGccqGH9aqpcqaIWaamaeaacqWGsbGucqGH9aqpcqWGtbWudaWgaaWcbaGaemyBa0gabeaakiabgkHiTiabdofatnaaBaaaleaacqWG1bqDaeqaaOGaeyyXICTaem4uam1aa0baaSqaaiabdwha1bqaaiabcocaJaaakiabgwSixlabdofatnaaBaaaleaacqWGTbqBaeqaaaaaaaa@48F3@

where *R *is the redundancy matrix and *S*_*u*_^# ^the Penrose pseudo-inverse of *S*_*u*_. In case inconsistency is detected, the method described in [38,39] can be used to estimate its importance. It is based upon statistical hypothesis testing to determine if redundancies are satisfied to within expected experimental error. The test is performed by calculating a consistency index *h *as follows:

h=εT⋅P−1⋅εε=−Rr⋅vmP=Rr⋅F⋅RrT
 MathType@MTEF@5@5@+=feaafiart1ev1aaatCvAUfKttLearuWrP9MDH5MBPbIqV92AaeXatLxBI9gBaebbnrfifHhDYfgasaacH8akY=wiFfYdH8Gipec8Eeeu0xXdbba9frFj0=OqFfea0dXdd9vqai=hGuQ8kuc9pgc9s8qqaq=dirpe0xb9q8qiLsFr0=vr0=vr0dc8meaabaqaciaacaGaaeqabaqabeGadaaakqaabeqaaiabdIgaOjabg2da9GGaciab=v7aLnaaCaaaleqabaGaemivaqfaaOGaeyyXICTaemiuaa1aaWbaaSqabeaacqGHsislcqaIXaqmaaGccqGHflY1cqWF1oqzaeaacqWF1oqzcqGH9aqpcqGHsislcqWGsbGudaWgaaWcbaGaemOCaihabeaakiabgwSixlabhAha2naaBaaaleaacqWHTbqBaeqaaaGcbaGaemiuaaLaeyypa0JaemOuai1aaSbaaSqaaiabdkhaYbqabaGccqGHflY1cqWGgbGrcqGHflY1cqWGsbGudaqhaaWcbaGaemOCaihabaGaemivaqfaaaaaaa@5601@

where *R*_*r *_is the reduced redundancy matrix and *F *the variances-covariances matrix of the measurements in **v**_**m**_. If a given set of measured fluxes **v**_**m **_fails the consistency check (*h>*χ^2^), then there is a (confidence level)% chance that either **v**_**m **_contains gross errors or the assumed stoichiometric matrix is incorrect. The χ^2 ^values for two confidence levels are given in Table 4. It must be noticed that some measured fluxes have no impact on the consistency of the system, so they are not considered in the analysis of consistency. These fluxes are called non-balanceable. On the contrary, a measured flux is called balanceable if the consistency of the system depends on its value. They can be detected as explained in [14]. The balanceable fluxes can be adjusted (or balanced) if they are inconsistent. Following the method described in [38,39], the adjusted fluxes can be calculated as:

**Table 4:** Chi-square values for two confident levels

**Degrees of freedom**	**90%**	**95%**
1	2.71	3.84
2	4.61	5.99
3	6.25	7.81
4	7.78	9.49

v^m=(I−F⋅RrT⋅P−1⋅Rr#)⋅vm
 MathType@MTEF@5@5@+=feaafiart1ev1aaatCvAUfKttLearuWrP9MDH5MBPbIqV92AaeXatLxBI9gBaebbnrfifHhDYfgasaacH8akY=wiFfYdH8Gipec8Eeeu0xXdbba9frFj0=OqFfea0dXdd9vqai=hGuQ8kuc9pgc9s8qqaq=dirpe0xb9q8qiLsFr0=vr0=vr0dc8meaabaqaciaacaGaaeqabaqabeGadaaakeaacuWH2bGDgaqcamaaBaaaleaacqWHTbqBaeqaaOGaeyypa0ZaaeWaaeaacqWGjbqscqGHsislcqWGgbGrcqGHflY1cqWGsbGudaqhaaWcbaGaemOCaihabaGaemivaqfaaOGaeyyXICTaemiuaa1aaWbaaSqabeaacqGHsislcqaIXaqmaaGccqGHflY1cqWGsbGudaqhaaWcbaGaemOCaihabaGaei4iamcaaaGccaGLOaGaayzkaaGaeyyXICTaeCODay3aaSbaaSqaaiabh2gaTbqabaaaaa@4C90@

where *R*_*r*_^# ^denoted the Penrose pseudo-inverse of the matrix *R*_*r*_. This equation provides the adjusted values for the balanceable fluxes and the original values for the non-balanceable ones.

### Auxiliary: Metabolic flux analysis

When the system (2) is determined but not redundant the unique solution can be calculated by using the inverse matrix of *S*_*u*_:

vu=−Su−1⋅Sm⋅vm
 MathType@MTEF@5@5@+=feaafiart1ev1aaatCvAUfKttLearuWrP9MDH5MBPbIqV92AaeXatLxBI9gBaebbnrfifHhDYfgasaacH8akY=wiFfYdH8Gipec8Eeeu0xXdbba9frFj0=OqFfea0dXdd9vqai=hGuQ8kuc9pgc9s8qqaq=dirpe0xb9q8qiLsFr0=vr0=vr0dc8meaabaqaciaacaGaaeqabaqabeGadaaakeaacqWH2bGDdaWgaaWcbaGaeCyDauhabeaakiabg2da9iabgkHiTiabdofatnaaDaaaleaacqWG1bqDaeaacqGHsislcqaIXaqmaaGccqGHflY1cqWGtbWudaWgaaWcbaGaemyBa0gabeaakiabgwSixlabhAha2naaBaaaleaacqWHTbqBaeqaaaaa@40E3@

When the system is determined but redundant, matrix *S*_*u *_is not invertible so the Penrose pseudo-inverse is used instead (providing a least squares solution):

vu=−Su#⋅Sm⋅vm
 MathType@MTEF@5@5@+=feaafiart1ev1aaatCvAUfKttLearuWrP9MDH5MBPbIqV92AaeXatLxBI9gBaebbnrfifHhDYfgasaacH8akY=wiFfYdH8Gipec8Eeeu0xXdbba9frFj0=OqFfea0dXdd9vqai=hGuQ8kuc9pgc9s8qqaq=dirpe0xb9q8qiLsFr0=vr0=vr0dc8meaabaqaciaacaGaaeqabaqabeGadaaakeaacqWH2bGDdaWgaaWcbaGaeCyDauhabeaakiabg2da9iabgkHiTiabdofatnaaDaaaleaacqWG1bqDaeaacqGGJaWiaaGccqGHflY1cqWGtbWudaWgaaWcbaGaemyBa0gabeaakiabgwSixlabhAha2naaBaaaleaacqWHTbqBaeqaaaaa@3FD4@

Finally, if system (2) is underdetermined, Metabolic Flux Analysis cannot be used. Only some fluxes may be uniquely calculable by using the method explained in [14].

## List of abbreviations

MFA: Metabolic flux analysis

FSA: Flux spectrum approach

FBA: Flux balance analysis

CHO: Chinese hamster ovary (cells)

AIS: Average along time of the interval size of an estimated flux.

## Authors' contributions

FL and JP designed the research, analyzed the results and conceptualized the manuscript. FL performed the estimation of non-measured fluxes and drafted the manuscript. JP supervised and coordinated the project. All the authors read and approved the final manuscript.

## Supplementary Material

Additional file 3 **Conversion of measured concentrations to measured fluxes**. Example of the calculation of the measured fluxes when the measurements of concentration are obtained at a high sample rate. Click here for file

Additional file 2 **Flux spectrum approach in the underdetermined and redundant case**. Example of the estimation of fluxes with the Flux Spectrum Approach in an Underdetermined and redundant case. Click here for file

Additional file 1 **Metabolic network description**. List of metabolites, reactions and stoichiometric matrixes. Click here for file

Additional file 4 **Mathematical model (SBML and matlab)**. The zip file contains the mathematical model of the metabolism of CHO cells in three different formats: a matlab script, an SBML model and a metatool file. Click here for file

Additional file 7 **Additional figures and tables**. Additional figures cited in the manuscript. Click here for file

Additional file 6 **Analysis of the unbalanced propagation of the uncertainty**. Dataset with the interval size of the estimations of each non-measured flux at each time instant. Click here for file

Additional file 8 **Analysis of the effect of the uncertainty of each measured flux**. Details and datasets of the indirect and direct analysis of the effect of the uncertainty of each measured flux. Click here for file

Additional file 5 **Implementation of the Flux Spectrum Approach**. The zip file contains a matlab script which implements the method to estimate the non-measured fluxes with the Flux Spectrum Approach and a simple example that illustrates how to use it. Click here for file
