# Supplementary material for: A procedure for the estimation over time of metabolic fluxes in scenarios where measurements are uncertain and/or insufficient
Source: BMC Bioinformatics. 2007 Oct 30;8:421. doi: 10.1186/1471-2105-8-421 (PMC2212668; doi:10.1186/1471-2105-8-421)
Supplement: Additional file 3 — Conversion of measured concentrations to measured fluxes. Example of the calculation of the measured fluxes when the measurements of concentration are obtained at a high sample rate. [file 1471-2105-8-421-S3.doc]

Additional file 3: Example of the conversion of measured concentrations to measured fluxes.

This example will address the conversion of the measured concentrations to measured fluxes when they are obtained at a high sample rate. This is the typical behaviour of online sensors, the ones which need be used when the conversion (and the whole procedure) is done online. Nevertheless, these sensors can be also used when the procedure is done offline, and therefore both operation modes will be studied herein.

Again, the data provided by (Provost et al., 2005) will be used. However, the original measurements have a very low sampling rate (24h) that do not correspond to the typical output of an online sensor. Hence, a modification of the original data has been created by adding intermediate noisy points (1 measurement per hour and white noise of ±10%). In order to keep this example simple, only one extracellular flux will be calculated (the flux of glucose). The growth rate of biomass will be also calculated to shown that its calculation is completely analogous.

**Figure 1.** Modified measurements of the cell density and the concentration of glucose. This data has been by generated by adding intermediate noisy points (1 measurement per hour and white noise of ±10%) to the measurements given in (Provost et al., 2005). The red points denotes the original measurements and the black points the added ones.

*Offline case*

In this case, filtering is advisable (since the sample rate is high with respect to the dynamics of the process). As the procedure is supposed to be done offline, a centred moving average filter (CMA) will be used. It provides the best results thanks to the use of not only past information but also future one (see methods). A window size of 12 has been chosen because it offers a good balance between noise rejection and true signal preservation. The filtered signal seems quite good: the noise has been rejected and the signal evolution is sensible (results are given in figure 2).

**Figure 2.** Filtered signals of the cell density (biomass) and the concentration of glucose.

Once the signals of concentrations have been filtered, the extracellular flux of glucose (and the growth rate) can be calculated by using two different approaches:

- *Approximating the derivative with a centred method*. At each time instant, the derivative is approximated with the middle point method (see methods) and then the value of the flux is working out from eq. 1. As the procedure is done offline, the middle point method is the most advisable (the approximation of the derivative is less noisy). Afterwards, the calculated signal of glucose flux (and growth rate) should be filtered to make it smooth. A centred moving average filter (CMA) is used (since the procedure is done offline). In this case, a window size of 10 has been chosen.
- *By using a high-gain non-linear observer*. A value of 0.05 for the parameter θ provides a good balance between noise rejection and basic signal preservation (see methods).

Results are given in Figure 3, where they are compared with the ones obtained from the original measurements (those without added noise). The signal of the glucose flux (and the growth rate) calculated with both methods is similar to the one obtained in absence of noise. As was expected, the middle point method, combined with a second step of filtering, provides better results than the observer. That is sensible because the observer uses only past information, while the first approach uses past and future information (not only in the middle point approximation, but also in the second step of filtering).

**Figure 3.** Calculated signals of glucose flux and biomass growth by using two different approaches. The results obtain from the original measurements of concentration (those without added noise) are depicted for the shake of comparison.

*Online case*

In this section, the online conversion of the measured concentrations into measured fluxes will be approached. This conversion has to be used when the whole procedure is done online (e.g., to monitor industrial processes). Remember that in this situation, at each time instant *k*, the procedure works as follows: 1) the online sensors provide the concentration measurements in *k*, 2) the signals of concentrations can be filtered by using past values (*k, k-1…k-i*), 3) the measured fluxes are calculated from the concentrations at *k, k-1…k-I*, and 4) the non-measured fluxes at time *k* are estimated by using the measured fluxes.

Firstly, the signals of measured concentrations (given in figure 1) should be filtered to reduce the noise (noticed that the sample rate seems high enough). At this point, three situations will be compared:

- - We assume that a delay in the calculation is not allowable: Use of SMA. The signal is calculated without delay (value for *k* is computed in *k*).
  - We assume that a certain delay is allowable: Use of a low-order CMA. The signal is calculated with the allowable delay (value for time *k* is calculated before *k+j*).
  - We assume that a large delay is allowable: Use of a high-order CMA (as was done in the offline case).

In the first case a large window size (24) provides a balance between noise-rejection and delay with respect to the true signal evolution. In the second one, only a little window (5) can be used because the allowable delay is restricted. In the third one, a window size of 12 is chosen (as a balance between noise rejection and true signal preservation). The results obtained in each case are given in figure 4.

**Figure 4.** Filtered signals of the cell density (biomass) and the concentration of glucose in three different scenarios.

When the high-order SMA filter is used, the filtering is done in time *k* and the noise-rejection is very good, but the calculated signal is delayed with respect to the original signal. If a low-order CMA filter is used, the filtering is done in time *k+5*, but the noise-rejection is quite good and the calculated signal is not delayed. Finally, if a high-order CMA filter is used, the filtering is done in time *k+12*, but the noise-rejection is very good and the calculated signal is not delayed. In order to determine which the most suitable filter is, the particularities of the available measurements and the operation mode need to be analyzed.

Once the signal of measured concentrations has been filtered, the extracellular flux of glucose (and the growth rate) is calculated by using three different approaches. The first two approaches use the signal of concentrations filtered with SMA to ensure that the estimations of fluxes in time *k* are obtained in *k*. The third approach use the signal of concentrations filtered with a low-order CMA which could be useful when a slight delay in the estimation is admissible (e.g., when the estimation of fluxes in time *k* are obtained in *k+5*).

- *Approximating the derivative with a backward method*. At each time instant, the derivative is approximated with the backward method (see methods), and then the value of the flux is working out from eq. 1. This approximation does not introduces any delay (value for *k* is computed with the values in *k-1* and *k*), but the calculated fluxes are noisier than the one obtained with centred approximations. Afterwards, the calculated signal of flux of glucose (and growth rate) should be filtered to make it smooth. A SMA is used to ensure that the estimation of fluxes in *k* is done in *k*. A window size of 24 has been chosen.
- *By using a high-gain non-linear observer*. It provides an interesting alternative to the backwards approximation. It obtain quite similar results (in terms of smoothness), but it provide some additional profits (e.g., simple tuning or guarantied stability). A value of 0.05 for the parameter θ provides a good balance between noise rejection and true signal preservation (see methods).
- *Approximating the derivative with a centred method*. At each time instant, the derivative is approximated with the middle point method (see methods) and then the value of the flux is working out from eq. 1. It provides the smoother signal but it introduces a unitary delay in the calculation (value for *k* is computed with the values in *k-1*, *k* and *k+1*). Afterwards, the calculated signal of flux of glucose (and growth rate) should be filtered with a CMA filter (with the same window size that was used to filter the concentrations, in order to avoid a bigger delay).

Results are given in Figure 5, where they are compared with the results obtained in the offline case. In addition, the delay in the calculation of each approach is given in table 1. The two first approaches, those which make the estimation in time *k*, provide similar results. The noise rejection is quite good, but the estimated signal is delayed with respect to the original signal. This delay can be reduced if the order of the used filters is decreased, but then the noise-rejection is lower (there is trade-off). This reduction of the noise-rejection power will be problematic, especially in the estimation of the glucose flux (because this flux is affected by the noise in the measurements of glucose and biomass). The third approach eliminates the delay with respect to the true signal evolution, but at the cost of a delayed estimation (the values at time *k* are obtained in time *k+5*) and a slightly lower noise-rejection.

In summary, it has been shown that there is not a universal solution for the conversion problem. In real applications, the particularities of the concentrations measurements (accuracy, sample rate, importance and characteristics of the noise, etcetera) and the operation mode (offline, online with an acceptable delay or purely online) will determine which the most suitable method is.

**Figure 5.** Calculated signals of glucose flux and biomass growth by using three different approaches. The results obtain in the offline case are depicted for the shake of comparison.

**Table 1.** Delay in the calculation of the flux (estimation of fluxes in *k* is obtained in *k+d*).

| *Case* | *Delay (d)* |
| --- | --- |
| Best offline | 23 |
| SMA24 + Euler B. + SMA24 | 0 |
| SMA24 + Observer | 0 |
| CMA5/7 + Euler M.P. + CMA5/7 | 11/15 |

**Practical Guide**

| **When the procedure is done offline:**   1. Assuming that noise exists, the decision of filtering or not depends on the sample rate: Filtering is only advisable when the sample rate is high with respect to the dynamics of the measured specie (to avoid the elimination of valuable true signal information). 2. If filtering is advisable (i.e., the signal is noisy and the sample rate is high enough):    - Choose always a centred filter, such as the centred moving average (CMA).    - The order of the filter must be chosen taken into account that there is a trade-off between noise-rejection and true signal preservation. The key issue is the sample rate: Only if it is high with respect to the dynamics of the measured specie, a high-order filter, which provides a better noise-rejection, can be used without eliminating valuable true signal information. 3. Calculation of fluxes from the measured concentrations:    - The use of a centred approximation is advisable because they provide the smoother signal (these methods calculate the value in the time instant *k* using past information *k-i* and future information *k+i*). Hence, if an Euler method is used, the middle point version should be chosen. 4. If the calculated fluxes are noisy and the sample rate is high enough, filter them.    - Again, choose a centred method, such as CMA. |
| --- |
| **When the procedure is done online:**   1. Assuming that noise exists, the decision of filtering or not depends on:    - The sample rate. Filtering is only advisable when the sample rate is high enough with respect to the dynamics of the measured specie (to avoid the elimination of valuable true signal information).    - The inconvenience of introducing delays. The use of filters introduces two kinds of delays: a delay in the calculation (value for *k* is computed in *k+i*) and a delay in the calculated signal (the calculated signal is delayed with respect to the true signal). The first one appears when centred methods are used, and the second one when backward methods are used. In some applications any delays can be inadmissible (e.g., when the signal is used as input of a control device). 2. If filtering is advisable, there are several alternatives depending on the degree of inconvenience of introducing delays. On the one hand, the use of future information (i.e., the use of centred methods) needs to considered. On the other hand, the order of the filter has to be chosen carefully.    - Use standard moving average filters (SMA) if a delay in the calculation is not allowable. The signal is calculated without delay (value for *k* is computed in *k*) but the calculated signal is delayed. In order to choose the order of the filter, it must be remember that: higher order implies a better noise rejection but a higher delay (and also a higher risk of eliminating valuable true signal information).    - Use centred moving average filters (CMA) if a large delay is allowable. The signal is calculated with a delay (value for *k* is computed in *k+i*) but the calculated signal is not delayed (is *centred*). In order to choose the order of the filter, it must be remember that, again: higher order implies a better noise rejection but a higher delay (and also a higher risk of eliminating valuable true signal information).    - If only a certain delay is allowable (value for *k* needs to be calculated before *k+j*) two options are possible: using a low-order (*j*) CMA or a high-order SMA. The decision here is: lower noise-rejection but non-delayed signal (CMA) or better noise-rejection but delayed signal (SMA). 3. Calculation of fluxes from the measured concentrations:    - Use a centred approximation (e.g. Euler middle point method). It provides the smoother signal but it introduces a unitary delay in the calculation (value for *k* is computed with the values in *k-1*, *k* and *k+1*). If this delay is allowable, this is the best option.    - Use backward approximation (e.g. Euler backwards method). It does not introduces any delay (value for *k* is computed with the values in *k-1* and *k*), but the calculated signal is noisier than the one obtained with centred approximations. Hence, this approximation is only useful when the unitary delay introduced by the centred approximation is not allowable.    - Use a dynamic observer. It provides an interesting alternative to the backwards approximations. It usually obtain quite similar results (in terms of smoothness), but it provides some additional profits (e.g., simple tuning or guarantied stability). 4. If the calculated fluxes are noisy they can be filtered*. The issues described in points 1 and 2 are also useful in this case. In addition, it must be taken into account that the delay in the calculation is not accumulated (i.e., if the measured concentrations are filtered with a CMA of order 5 and the calculated fluxes are filtered with another CMA of order 5, the total delay of the calculation is 5 time instants), while the delay in the calculated signal is accumulative.   **when fluxes are calculate by using a dynamic observer the do not need to be filtered because the observer includes a filter effect through its unique parameter θ.* |
