# Supplementary material for: A procedure for the estimation over time of metabolic fluxes in scenarios where measurements are uncertain and/or insufficient
Source: BMC Bioinformatics. 2007 Oct 30;8:421. doi: 10.1186/1471-2105-8-421 (PMC2212668; doi:10.1186/1471-2105-8-421)
Supplement: Additional file 2 — Flux spectrum approach in the underdetermined and redundant case. Example of the estimation of fluxes with the Flux Spectrum Approach in an Underdetermined and redundant case. [file 1471-2105-8-421-S2.doc]

Additional file 2: Example of the estimation of fluxes in an underdetermined and redundant case.

A simple example will be used to demonstrate that the procedure can be applied when the system is simultaneously underdetermined and redundant. A toy network and its stoichiometric matrix *S* are given in figure 1A. There are 4 metabolites (*m*) and 8 reactions (*n*). All reactions are irreversible except reaction 4. Remember that the mass balance at pseudo steady-state is given by eq. 2. We assume that fluxes *v_1_, v_4_, v_7_* and *v_8_* are measured. Then, the number of unknown fluxes is 4 (*nu*) and the rank of *Nu* is 3. Hence:

Rank(*Nu*)<*m*  (3<4) → Redundant

Rank(*Nu*)<*nu* (3<4) → Underdetermined

The system results underdetermined, but redundant. That means that metabolic flux analysis (MFA) cannot be used to calculate the non-measured fluxes. However the flux-spectrum approach (FSA) can be used instead. Moreover, as the system is redundant, the consistency of the measurements can be analyzed. As an example, we can assume that, at some time instant *k*, the measured values are:

$$v_{1}=0.91$$

$$v_{4}=0.88$$

$$v_{7}=0.89$$

$$v_{8}=1.85$$

Firstly, the importance of the inconsistency of these measurements can be estimated with the χ-square method (see methods). They passed the consistency check for a confidence level of 95% (the obtained value of *h*, 0.595, is minor than 3.84). Afterwards, FSA can be used to estimate the non-measured fluxes. Before applying FSA we can choose between adjust the inconsistent measurements or define a band of uncertainty around them. We chose the second approach, and the non-measured fluxes were estimated considering a band of uncertainty of ±5% around the measured values (results are given in figure 1B).

The main conclusion is that, although the system is underdetermined, FSA provides a precise estimation of the non-measured fluxes. In addition, this estimation is reliable because measurements consistency is taken into account: We use the χ-square method to reject the presence of gross errors and we demonstrate that a consistent set of measurements exists near the original values (i.e., within the uncertainty band ±5%). Moreover, to consider the uncertainty of the measured values also improves the reliability of our results.

It is also remarkable the benefits of introducing the irreversibility constraints. In fact, these constraints are partially responsible of the quality of the results obtained in this example (they impose hard restrictions to the region of possible flux distributions).

**Figure 1.** The flux-spectrum approach (FSA) when the system is underdetermined but redundant. (A) Simple toy network and its stoichiometric matrix. (B) Estimation of the non-measured fluxes by using FSA. The black intervals represent the measured fluxes and its uncertainty band, and the blue intervals the intervals of possible values for the non-measured fluxes calculated with FSA.
