# Supplementary material for: A procedure for the estimation over time of metabolic fluxes in scenarios where measurements are uncertain and/or insufficient
Source: BMC Bioinformatics. 2007 Oct 30;8:421. doi: 10.1186/1471-2105-8-421 (PMC2212668; doi:10.1186/1471-2105-8-421)
Supplement: Additional file 1 — Metabolic network description. List of metabolites, reactions and stoichiometric matrixes. [file 1471-2105-8-421-S1.doc]

Additional file 1: list of metabolites, reactions and stoichiometric matrixes.

List of initial substrates and extracellular and intracellular products.

| G Glucose initial substrates | Q Glutamine initial substrates |
| --- | --- |
| L Lactate extracell. product | A Alanine extracell. product |
| NH4 Ammonia extracell. product | CO2 Carbon dioxide extracell. product |
| Pu Purine intracell. product | Py Pyrimidine intracell. product |

List of internal metabolites. The number in brackets denotes the corresponding row of *S*.

| G6P (1) Glucose-6-phosphate | G3P (3) Glyceraldehyde-3-phosphate |
| --- | --- |
| DAP (2) Dihydroxy-acetone Phosphate | Pyr (5) Pyruvate |
| R5P (4) Ribose-5-Phosphate | ACA (6) Acetyl-coenzyme A |
| Cit (7) Citrate | Oxa (11) Oxaloacetate |
| Mal (9) Malate | aKG (8) α-ketoglutarate |
| Glu (10) Glutamate | Asp (12) Aspartate |

Reactions.

| E1 : G => G6P .  E2 : G6P <=> DAP + G3P .  E3 : G6P => R5P + CO2 .  E4 : DAP <=> G3P .  E5 : G3P <=> Pyr .  E6 : Pyr <=> L .  E7 : Pyr + Glu< => aKG + A .  E8 : Pyr => ACA + CO2 .  E9 : ACA + Oxa => Cit .  E10 : Cit => aKG + CO2 . | E11 : aKG => Mal + CO2 .  E12 : Mal => Oxa .  E13 : Mal => Pyr + CO2 .  E14 : Oxa + Glu = Asp + aKG .  E15 : Glu => aKG + NH4 .  E16 : Q => Glu + NH4 .  E17 : Q + R5P + Asp => Pu .  E18 : 2Q + R5P + Asp => py . |
| --- | --- |

Stoichiometric matrix. Each column corresponds to a reaction (1-18) and each row to an intreacellular metabolite (1-12).

$$S=\left[\begin{matrix} 1 & \text{-1} & \text{-1} & 0 & 0 & 0 & 0 & 0 & 0 & 0 & 0 & 0 & 0 & 0 & 0 & 0 & 0 & 0 \\ 0 & 1 & 0 & \text{-1} & 0 & 0 & 0 & 0 & 0 & 0 & 0 & 0 & 0 & 0 & 0 & 0 & 0 & 0 \\ 0 & 1 & 0 & 1 & \text{-1} & 0 & 0 & 0 & 0 & 0 & 0 & 0 & 0 & 0 & 0 & 0 & 0 & 0 \\ 0 & 0 & 1 & 0 & 0 & 0 & 0 & 0 & 0 & 0 & 0 & 0 & 0 & 0 & 0 & 0 & \text{-1} & \text{-1} \\ 0 & 0 & 0 & 0 & 1 & \text{-1} & \text{-1} & -1 & 0 & 0 & 0 & 0 & 1 & 0 & 0 & 0 & 0 & 0 \\ 0 & 0 & 0 & 0 & 0 & 0 & 0 & 1 & \text{-1} & 0 & 0 & 0 & 0 & 0 & 0 & 0 & 0 & 0 \\ 0 & 0 & 0 & 0 & 0 & 0 & 0 & 0 & 1 & \text{-1} & 0 & 0 & 0 & 0 & 0 & 0 & 0 & 0 \\ 0 & 0 & 0 & 0 & 0 & 0 & 1 & 0 & 0 & 1 & \text{-1} & 0 & 0 & 1 & 1 & 0 & 0 & 0 \\ 0 & 0 & 0 & 0 & 0 & 0 & 0 & 0 & 0 & 0 & 1 & \text{-1} & \text{-1} & 0 & 0 & 0 & 0 & 0 \\ 0 & 0 & 0 & 0 & 0 & 0 & \text{-1} & 0 & 0 & 0 & 0 & 0 & 0 & \text{-1} & \text{-1} & 1 & 0 & 0 \\ 0 & 0 & 0 & 0 & 0 & 0 & 0 & 0 & \text{-1} & 0 & 0 & 1 & 0 & \text{-1} & 0 & 0 & 0 & 0 \\ 0 & 0 & 0 & 0 & 0 & 0 & 0 & 0 & 0 & 0 & 0 & 0 & 0 & 1 & 0 & 0 & \text{-1} & \text{-1} \end{matrix}\right]$$

The linkage with extracellular fluxes. Each column corresponds to a reaction (1-18) and rows correspond to the extracellular fluxes.

| *Flux of G = -v1* (*)  *Flux of L = v6* (*)  *Flux of A = v7*  (*)  *Flux of NH4 = v15 + v16* (v19) | *Flux of Q = -v6 - v17 - 2*v18*  (v20)  *Flux of CO2 = v3 + v8 + v10 + v11 + v13* (v21)  *Vpury=Vpiri or v17 - v18=0*  (v22) |
| --- | --- |

(*) Values for fluxes of G , L and A are directly substituted in matrix *S.*

The Extended system is defined as follows:
