# Supplementary material for: A procedure for the estimation over time of metabolic fluxes in scenarios where measurements are uncertain and/or insufficient
Source: BMC Bioinformatics. 2007 Oct 30;8:421. doi: 10.1186/1471-2105-8-421 (PMC2212668; doi:10.1186/1471-2105-8-421)
Supplement: Additional file 7 — Additional figures and tables. Additional figures cited in the manuscript. [file 1471-2105-8-421-S7.doc]

# Step 3 (S1): Estimation of fluxes when measurements are almost sufficient

**Figure A1.** Effect of slight deviations in measured fluxes over MFA estimations*.* The figure shows the variation over each estimated flux caused by a deviation of 5% in the original measurements of *v_1_ (G)* and *v_6_ (L)*.

**Figure A2.** Considered band of uncertainty. (A) The absolute error considered with the band (black solid line). (B) The relative error considered with the band (black solid line).

# Step 3 (S2): Estimation of fluxes when measurements are sufficient

**Figure A3.** Assumed evolution of *v_21_ (CO2)*. For each time instant *k*, except 24h and 168h, the values given by MFA are used as measured values. The values at 24h and 168h are calculated by approximating a spline curve.

**Table A1.** χ-square consistency check. Calculated values of *h*.

| *time* | *0h* | *24h* | *48h* | *72h* | *96h* | *120h* | *144h* | *168h* | *192h* |
| --- | --- | --- | --- | --- | --- | --- | --- | --- | --- |
| *Value h^a^* | 0 | 3.02 | 0.0001 | 0 | 0.0014 | 0.0021 | 2.9349 | 37.94 | 0 |

^a^ Values bigger than 3.84 fail the consistency check for a confidence level of 0.95 (i.e. h>χ^2^).

# Step 3 (S3): Estimation of fluxes when measurements are insufficient

**Figure A4.** Evolution of the non-measured fluxes estimated with FSA in two underdetermined cases. The evolution is characterized by using the middle point of the interval of possible values at each time instant. The results obtained when five fluxes are measured (*v_1_ , v_6_ , v_7_, v_19_* and  *v_20_*) are depicted in green (second interval). The results obtained when four fluxes are measured (*v_1_ , v_6_ , v_7_* and  *v_19_*) are depicted in blue (third interval). The results obtained in the determined case (when six fluxes were measured) are included for the shake of comparison (in black).

# Characteristics of the propagation of the uncertainty


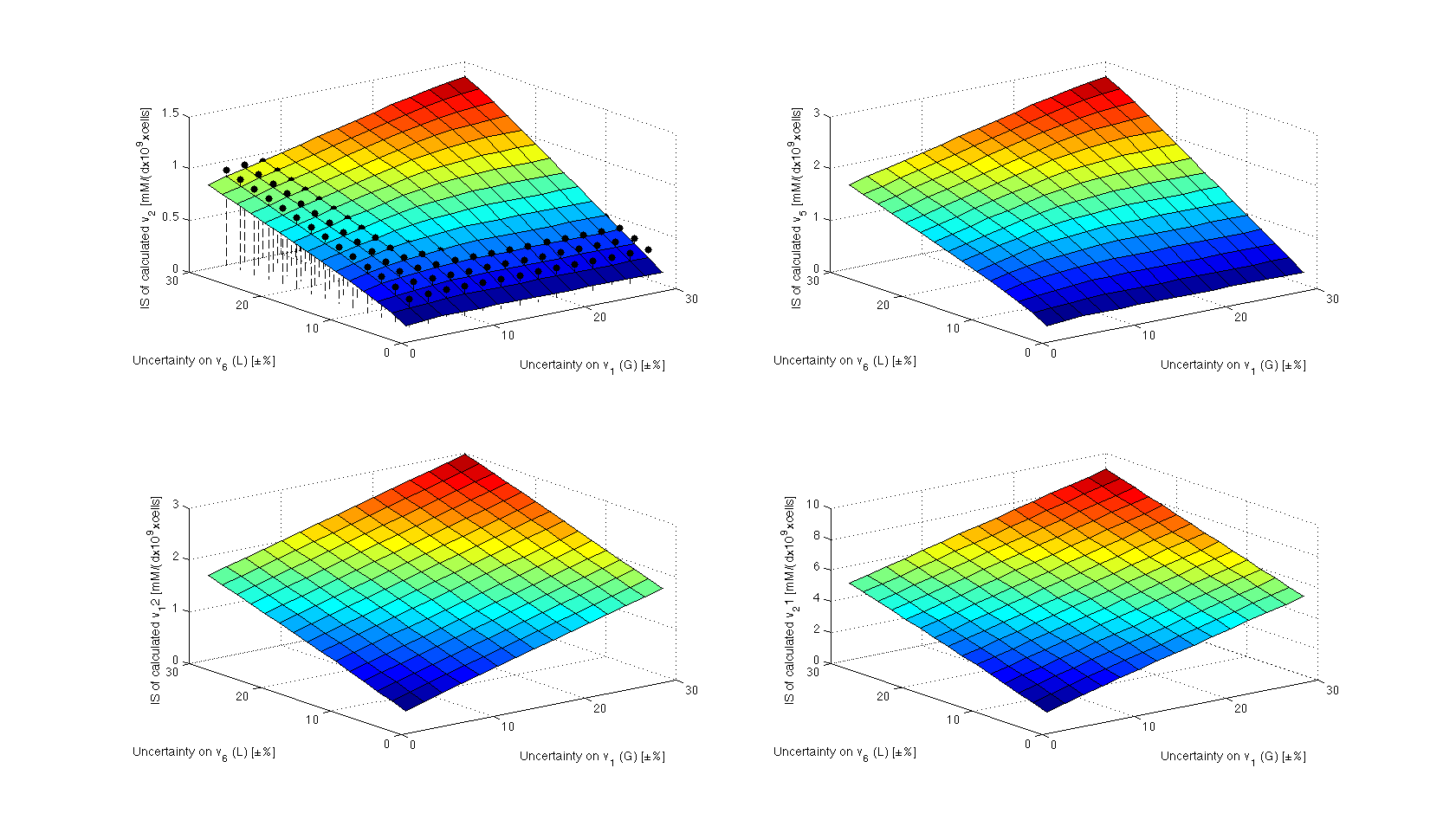


**Figure A5.** Effect over the estimated fluxes *v_2_*, *v_5_*, *v_12_* and *v_21_* of the uncertainty on the measured values of v_1_ and v_6_. The surfaces represent the averaged interval size of the estimated v_2_ when different degrees of uncertainty are considered for the measured fluxes v_1_ and v_6_. In the top left figure the results of summing up the independents effects (IS) of *v_6_* uncertainty and *v_1_* uncertainty are depicted with black dots.


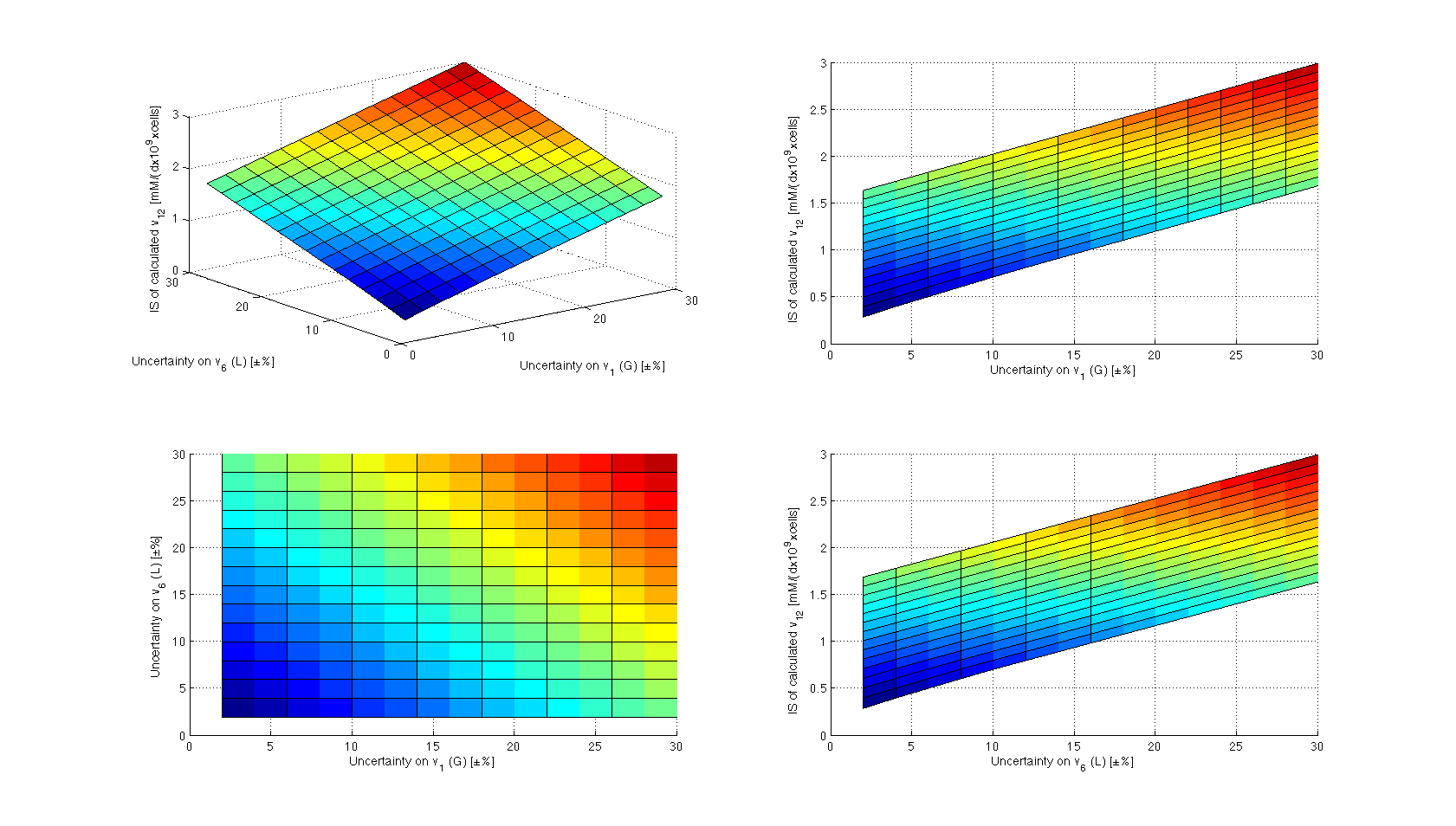


**Figure A6.** Effect over the estimated flux v_12_ of the uncertainty on the measured values of v_1_ and v_6_. The surfaces (and its projections) represent the averaged interval size of the estimated v_12_ when different degrees of uncertainty are considered for the measured fluxes v_1_ and v_6_.

## 
