# Supplementary material for: A procedure for the estimation over time of metabolic fluxes in scenarios where measurements are uncertain and/or insufficient
Source: BMC Bioinformatics. 2007 Oct 30;8:421. doi: 10.1186/1471-2105-8-421 (PMC2212668; doi:10.1186/1471-2105-8-421)
Supplement: Additional file 6 — Analysis of the unbalanced propagation of the uncertainty. Dataset with the interval size of the estimations of each non-measured flux at each time instant. [file 1471-2105-8-421-S6.doc]

**Analysis of the unbalanced propagation of the uncertainty**

Datasets with the interval size of the estimations of each non-measured flux at each time instant.

- Table 1. Results for each calculated flux along time (determined case).
- Table 2. Results for each calculated flux along time (overdetermined case).
- Table 3. Summary of results for each time instant (determined and overdetermined cases).
- Table 4. Summary of results for each calculated flux (determined and overdetermined cases).

*(tables are given in the following pages)*

Table 1. Results for each calculated flux along time (determined case).

|  |  |  | **0h** | | **24h** | | **48h** | | **72h** | | **96h** | | **120h** | | **144h** | | **168h** | | **192h** | |  |  | **mean** | | **median** | |
| --- | --- | --- | --- | --- | --- | --- | --- | --- | --- | --- | --- | --- | --- | --- | --- | --- | --- | --- | --- | --- | --- | --- | --- | --- | --- | --- |
|  | *Max [a]* |  | *IS [^a^]* | *[%b]* | *IS [^a^]* | *[%b]* | *IS [^a^]* | *[%b]* | *IS [^a^]* | *[%b]* | *IS [^a^]* | *[%b]* | *IS [^a^]* | *[%b]* | *IS [^a^]* | *[%b]* | *IS [^a^]* | *[%b]* | *IS [^a^]* | *[%b]* |  |  | *IS [^a^]* | *[%b]* | *IS [^a^]* | *[%b]* |
| **v_2_** | 6,04 |  | 1,02 | 16,9% | 0,58 | 9,5% | 0,62 | 10,2% | 0,33 | 5,5% | 0,21 | 3,5% | 0,16 | 2,6% | 0,16 | 2,6% | 0,16 | 2,7% | 0,16 | 2,7% |  |  | 0,38 | 6,2% | 0,21 | 3,5% |
| **v_3_** | 0,85 |  | 0,34 | 40,1% | 0,21 | 24,8% | 0,13 | 15,6% | 0,13 | 14,7% | 0,11 | 12,8% | 0,06 | 6,8% | 0,06 | 6,5% | 0,06 | 7,2% | 0,06 | 7,5% |  |  | 0,13 | 15,1% | 0,11 | 12,8% |
| **v_4_** | 6,04 |  | 1,02 | 16,9% | 0,58 | 9,5% | 0,62 | 10,2% | 0,33 | 5,5% | 0,21 | 3,5% | 0,16 | 2,6% | 0,16 | 2,6% | 0,16 | 2,7% | 0,16 | 2,7% |  |  | 0,38 | 6,2% | 0,21 | 3,5% |
| **v_5_** | 12,08 |  | 2,04 | 16,9% | 1,15 | 9,5% | 1,23 | 10,2% | 0,67 | 5,5% | 0,42 | 3,5% | 0,32 | 2,6% | 0,31 | 2,6% | 0,32 | 2,7% | 0,33 | 2,7% |  |  | 0,75 | 6,2% | 0,42 | 3,5% |
| **v_8_** | 1,17 |  | 2,33 | 200,0% | 1,12 | 95,7% | 1,66 | 142,8% | 1,13 | 96,9% | 0,73 | 62,6% | 0,62 | 53,1% | 0,62 | 52,9% | 0,63 | 54,3% | 0,64 | 55,0% |  |  | 1,05 | 90,4% | 0,73 | 62,6% |
| **v_9_** | 1,17 |  | 2,33 | 200,0% | 1,12 | 95,7% | 1,66 | 142,8% | 1,13 | 96,9% | 0,73 | 62,6% | 0,62 | 53,1% | 0,62 | 52,9% | 0,63 | 54,3% | 0,64 | 55,0% |  |  | 1,05 | 90,4% | 0,73 | 62,6% |
| **v_10_** | 1,17 |  | 2,33 | 200,0% | 1,12 | 95,7% | 1,66 | 142,8% | 1,13 | 96,9% | 0,73 | 62,6% | 0,62 | 53,1% | 0,62 | 52,9% | 0,63 | 54,3% | 0,64 | 55,0% |  |  | 1,05 | 90,4% | 0,73 | 62,6% |
| **v_11_** | 3,77 |  | 2,62 | 69,6% | 1,34 | 35,6% | 1,85 | 49,2% | 1,31 | 34,7% | 0,79 | 21,0% | 0,66 | 17,6% | 0,67 | 17,7% | 0,68 | 18,1% | 0,69 | 18,3% |  |  | 1,18 | 31,3% | 0,79 | 21,0% |
| **v_12_** | 1,85 |  | 2,34 | 126,4% | 1,13 | 60,9% | 1,68 | 90,4% | 1,10 | 59,2% | 0,64 | 34,5% | 0,56 | 30,2% | 0,56 | 30,3% | 0,57 | 30,8% | 0,58 | 31,2% |  |  | 1,02 | 54,9% | 0,64 | 34,5% |
| **v_13_** | 1,81 |  | 0,48 | 26,7% | 0,33 | 18,3% | 0,24 | 13,4% | 0,24 | 13,1% | 0,15 | 8,3% | 0,10 | 5,6% | 0,10 | 5,8% | 0,11 | 6,1% | 0,11 | 6,3% |  |  | 0,21 | 11,5% | 0,15 | 8,3% |
| **v_14_** | 0,85 |  | 0,34 | 40,1% | 0,21 | 24,8% | 0,13 | 15,6% | 0,13 | 14,7% | 0,11 | 12,8% | 0,06 | 6,8% | 0,06 | 6,5% | 0,06 | 7,2% | 0,06 | 7,5% |  |  | 0,13 | 15,1% | 0,11 | 12,8% |
| **v_15_** | 1,11 |  | 0,38 | 34,6% | 0,23 | 20,7% | 0,14 | 12,9% | 0,14 | 12,4% | 0,09 | 7,7% | 0,09 | 8,1% | 0,10 | 9,4% | 0,09 | 8,2% | 0,08 | 7,6% |  |  | 0,15 | 13,5% | 0,10 | 9,4% |
| **v_16_** | 2,67 |  | 0,29 | 10,9% | 0,18 | 6,8% | 0,12 | 4,4% | 0,11 | 4,2% | 0,11 | 4,2% | 0,06 | 2,1% | 0,06 | 2,1% | 0,06 | 2,3% | 0,07 | 2,5% |  |  | 0,12 | 4,4% | 0,11 | 4,2% |
| **v_17_** | 0,43 |  | 0,22 | 51,8% | 0,16 | 36,5% | 0,12 | 27,3% | 0,11 | 26,4% | 0,08 | 19,3% | 0,05 | 12,7% | 0,05 | 12,4% | 0,06 | 13,1% | 0,06 | 13,3% |  |  | 0,10 | 23,6% | 0,08 | 19,3% |
| **v_18_** | 0,43 |  | 0,20 | 45,9% | 0,13 | 30,7% | 0,09 | 21,5% | 0,09 | 20,5% | 0,07 | 16,8% | 0,03 | 7,3% | 0,03 | 6,9% | 0,03 | 7,9% | 0,04 | 8,3% |  |  | 0,08 | 18,4% | 0,07 | 16,8% |
| **v_21_** | 8,70 |  | 7,56 | 87,0% | 3,79 | 43,5% | 5,36 | 61,6% | 3,73 | 42,9% | 2,31 | 26,6% | 1,94 | 22,4% | 1,95 | 22,4% | 2,00 | 23,0% | 2,02 | 23,3% |  |  | 3,41 | 39,2% | 2,31 | 26,6% |
|  |  |  |  |  |  |  |  |  |  |  |  |  |  |  |  |  |  |  |  |  |  |  |  |  |  |  |
| **mean** | |  | 1,62 | 74,0% | 0,84 | 38,7% | 1,08 | 48,2% | 0,74 | 34,4% | 0,47 | 22,6% | 0,38 | 17,9% | 0,38 | 17,9% | 0,39 | 18,4% | 0,40 | 18,7% |  |  | 0,70 | 32,3% |  |  |
| **median** | |  | 1,02 | 43,0% | 0,58 | 27,7% | 0,62 | 18,5% | 0,33 | 17,6% | 0,21 | 14,8% | 0,16 | 7,7% | 0,16 | 8,2% | 0,16 | 8,0% | 0,16 | 7,9% |  |  |  |  |  |  |
|  |  |  |  |  |  |  |  |  |  |  |  |  |  |  |  |  |  |  |  |  |  |  |  |  |  |  |

Max: Maximum value of the estimated flux along time;

IS: Interval size of the estimated flux;

^a^ in [mM/(dx10^9^xcells)];

^b^ interval size w.r.t. maximum value of the flux along time.

Table 2. Results for each calculated flux along time (overdetermined case).

|  |  |  | **0h** | | **24h** | | **48h** | | **72h** | | **96h** | | **120h** | | **144h** | | **168h** | | **192h** | |  |  | **mean** | | **median** | |
| --- | --- | --- | --- | --- | --- | --- | --- | --- | --- | --- | --- | --- | --- | --- | --- | --- | --- | --- | --- | --- | --- | --- | --- | --- | --- | --- |
|  | *Max [a]* |  | *IS [^a^]* | *[%b]* | *IS [^a^]* | *[%b]* | *IS [^a^]* | *[%b]* | *IS [^a^]* | *[%b]* | *IS [^a^]* | *[%b]* | *IS [^a^]* | *[%b]* | *IS [^a^]* | *[%b]* | *IS [^a^]* | *[%b]* | *IS [^a^]* | *[%b]* |  |  | *IS [^a^]* | *[%b]* | *IS [^a^]* | *[%b]* |
| **v_2_** | 6,03 |  | 0,92 | 15,2% | 0,43 | 7,2% | 0,60 | 10,0% | 0,32 | 5,3% | 0,21 | 3,5% | 0,10 | 1,6% | 0,13 | 2,1% | 0,07 | 1,1% | 0,11 | 1,9% |  |  | 0,32 | 5,3% | 0,21 | 3,5% |
| **v_3_** | 0,86 |  | 0,33 | 38,3% | 0,20 | 23,2% | 0,12 | 14,0% | 0,11 | 13,1% | 0,11 | 12,6% | 0,06 | 6,8% | 0,06 | 6,5% | 0,06 | 7,2% | 0,06 | 7,4% |  |  | 0,12 | 14,3% | 0,11 | 12,6% |
| **v_4_** | 6,03 |  | 0,92 | 15,2% | 0,43 | 7,2% | 0,60 | 10,0% | 0,32 | 5,3% | 0,21 | 3,5% | 0,10 | 1,6% | 0,13 | 2,1% | 0,07 | 1,1% | 0,11 | 1,9% |  |  | 0,32 | 5,3% | 0,21 | 3,5% |
| **v_5_** | 12,06 |  | 1,83 | 15,2% | 0,87 | 7,2% | 1,21 | 10,0% | 0,64 | 5,3% | 0,42 | 3,5% | 0,20 | 1,6% | 0,25 | 2,1% | 0,14 | 1,1% | 0,23 | 1,9% |  |  | 0,64 | 5,3% | 0,42 | 3,5% |
| **v_8_** | 0,71 |  | 0,49 | 68,7% | 0,34 | 47,0% | 0,26 | 36,0% | 0,23 | 32,2% | 0,20 | 27,6% | 0,14 | 19,0% | 0,15 | 20,9% | 0,12 | 17,4% | 0,16 | 22,0% |  |  | 0,23 | 32,3% | 0,20 | 27,6% |
| **v_9_** | 0,71 |  | 0,49 | 68,7% | 0,34 | 47,0% | 0,26 | 36,0% | 0,23 | 32,2% | 0,20 | 27,6% | 0,14 | 19,0% | 0,15 | 20,9% | 0,12 | 17,4% | 0,16 | 22,0% |  |  | 0,23 | 32,3% | 0,20 | 27,6% |
| **v_10_** | 0,71 |  | 0,49 | 68,7% | 0,34 | 47,0% | 0,26 | 36,0% | 0,23 | 32,2% | 0,20 | 27,6% | 0,14 | 19,0% | 0,15 | 20,9% | 0,12 | 17,4% | 0,16 | 22,0% |  |  | 0,23 | 32,3% | 0,20 | 27,6% |
| **v_11_** | 3,07 |  | 0,35 | 11,5% | 0,24 | 7,7% | 0,18 | 5,8% | 0,15 | 5,0% | 0,13 | 4,3% | 0,11 | 3,4% | 0,11 | 3,7% | 0,10 | 3,1% | 0,12 | 3,8% |  |  | 0,16 | 5,4% | 0,13 | 4,3% |
| **v_12_** | 1,26 |  | 0,63 | 49,9% | 0,38 | 30,4% | 0,28 | 22,3% | 0,25 | 19,9% | 0,17 | 13,8% | 0,10 | 8,0% | 0,13 | 10,0% | 0,08 | 6,6% | 0,13 | 10,6% |  |  | 0,24 | 19,1% | 0,17 | 13,8% |
| **v_13_** | 1,81 |  | 0,48 | 26,4% | 0,32 | 17,9% | 0,24 | 13,1% | 0,23 | 12,8% | 0,15 | 8,2% | 0,08 | 4,4% | 0,10 | 5,4% | 0,05 | 3,0% | 0,11 | 5,8% |  |  | 0,20 | 10,8% | 0,15 | 8,2% |
| **v_14_** | 0,86 |  | 0,33 | 38,3% | 0,20 | 23,2% | 0,12 | 14,0% | 0,11 | 13,1% | 0,11 | 12,6% | 0,06 | 6,8% | 0,06 | 6,5% | 0,06 | 7,2% | 0,06 | 7,4% |  |  | 0,12 | 14,3% | 0,11 | 12,6% |
| **v_15_** | 1,11 |  | 0,38 | 34,1% | 0,22 | 20,2% | 0,14 | 12,4% | 0,13 | 11,8% | 0,08 | 7,5% | 0,09 | 8,1% | 0,10 | 9,5% | 0,09 | 8,2% | 0,08 | 7,6% |  |  | 0,15 | 13,3% | 0,10 | 9,5% |
| **v_16_** | 2,67 |  | 0,28 | 10,6% | 0,17 | 6,5% | 0,11 | 4,2% | 0,11 | 4,0% | 0,11 | 4,0% | 0,06 | 2,1% | 0,06 | 2,1% | 0,06 | 2,3% | 0,07 | 2,5% |  |  | 0,11 | 4,3% | 0,11 | 4,0% |
| **v_17_** | 0,44 |  | 0,19 | 42,9% | 0,12 | 28,2% | 0,09 | 19,3% | 0,08 | 18,4% | 0,08 | 17,9% | 0,05 | 12,3% | 0,05 | 12,0% | 0,06 | 12,6% | 0,06 | 12,9% |  |  | 0,09 | 19,6% | 0,08 | 17,9% |
| **v_18_** | 0,42 |  | 0,18 | 42,5% | 0,11 | 26,9% | 0,07 | 17,5% | 0,07 | 16,5% | 0,05 | 12,5% | 0,02 | 5,0% | 0,02 | 4,7% | 0,02 | 5,4% | 0,02 | 5,7% |  |  | 0,06 | 15,2% | 0,05 | 12,5% |
|  |  |  |  |  |  |  |  |  |  |  |  |  |  |  |  |  |  |  |  |  |  |  |  |  |  |  |
| **mean** | |  | 0,52 | 34,1% | 0,29 | 21,7% | 0,28 | 16,3% | 0,20 | 14,2% | 0,15 | 11,7% | 0,09 | 7,4% | 0,10 | 8,1% | 0,08 | 6,9% | 0,10 | 8,5% |  |  | 0,20 | 14,3% |  |  |
| **median** | |  | 0,48 | 38,3% | 0,32 | 23,2% | 0,24 | 14,0% | 0,23 | 13,1% | 0,15 | 12,5% | 0,10 | 6,8% | 0,11 | 6,5% | 0,07 | 6,6% | 0,11 | 7,4% |  |  |  |  |  |  |

Max: Maximum value of the estimated flux along time;

IS: Interval size of the estimated flux;

^a^ in [mM/(dx10^9^xcells)];

^b^ interval size w.r.t. maximum value of the flux along time.

Table 3. Summary of results for each time instant (determined and overdetermined cases).

|  | **Det.** | | **Overdet.** | |  | **Comparative** | |
| --- | --- | --- | --- | --- | --- | --- | --- |
|  | *MIS [^a^]* | *MIS [%^b^]* | *MIS [^a^]* | *MIS [%^b^]* |  | *Diff. IS [^a^]* | *Diff. [%]* |
| **0h** | 1,617 | 73,99% | 0,518 | 34,14% |  | 1,099 | 67,96% |
| **24h** | 0,835 | 38,66% | 0,295 | 21,68% |  | 0,540 | 64,69% |
| **48h** | 1,083 | 48,18% | 0,283 | 16,30% |  | 0,799 | 73,85% |
| **72h** | 0,737 | 34,37% | 0,202 | 14,19% |  | 0,536 | 72,66% |
| **96h** | 0,468 | 22,64% | 0,151 | 11,66% |  | 0,317 | 67,75% |
| **120h** | 0,382 | 17,93% | 0,089 | 7,42% |  | 0,293 | 76,71% |
| **144h** | 0,382 | 17,90% | 0,102 | 8,08% |  | 0,280 | 73,23% |
| **168h** | 0,392 | 18,43% | 0,077 | 6,94% |  | 0,315 | 80,31% |
| **192h** | 0,397 | 18,68% | 0,103 | 8,46% |  | 0,295 | 74,18% |
|  |  |  |  |  |  |  |  |
| **mean** | 0,699 | 32,31% | 0,202 | 14,32% |  | 0,497 | 71,09% |

MIS: Mean interval size of the estimated fluxes at each time instant;

Diff: Difference between determined and overdetermined cases;

^a^ in [mM/(dx10^9^xcells)]; ^b^ mean of the interval sizes expressed w.r.t. the maximum value of each calculated flux.

Table 4. Summary of results for each calculated flux (determined and overdetermined cases).

|  | **Det.** | | | **Overdet.** | | |  |  | **Comparative** | |
| --- | --- | --- | --- | --- | --- | --- | --- | --- | --- | --- |
|  | *Max [^a^]* | *MIS [^a^]* | *MIS [%^b^]* | *Max [^a^]* | *MIS [^a^]* | *MIS [%^b^]* |  |  | *Diff. [^a^]* | *Diff. [%]* |
| **v_2_** | 6,041 | 0,377 | 6,25% | 6,032 | 0,321 | 5,32% |  |  | 0,057 | 14,97% |
| **v_3_** | 0,853 | 0,129 | 15,12% | 0,859 | 0,123 | 14,35% |  |  | 0,006 | 4,41% |
| **v_4_** | 6,041 | 0,377 | 6,25% | 6,032 | 0,321 | 5,32% |  |  | 0,057 | 14,97% |
| **v_5_** | 12,081 | 0,755 | 6,25% | 12,065 | 0,642 | 5,32% |  |  | 0,113 | 14,98% |
| **v_8_** | 1,166 | 1,053 | 90,37% | 0,715 | 0,231 | 32,32% |  |  | 0,822 | 78,07% |
| **v_9_** | 1,166 | 1,053 | 90,37% | 0,715 | 0,231 | 32,32% |  |  | 0,822 | 78,07% |
| **v_10_** | 1,166 | 1,053 | 90,37% | 0,715 | 0,231 | 32,32% |  |  | 0,822 | 78,07% |
| **v_11_** | 3,769 | 1,180 | 31,30% | 3,073 | 0,165 | 5,37% |  |  | 1,015 | 86,02% |
| **v_12_** | 1,854 | 1,017 | 54,89% | 1,263 | 0,241 | 19,05% |  |  | 0,777 | 76,34% |
| **v_13_** | 1,813 | 0,209 | 11,52% | 1,809 | 0,195 | 10,78% |  |  | 0,014 | 6,58% |
| **v_14_** | 0,853 | 0,129 | 15,12% | 0,859 | 0,123 | 14,35% |  |  | 0,006 | 4,41% |
| **v_15_** | 1,113 | 0,150 | 13,52% | 1,109 | 0,147 | 13,27% |  |  | 0,003 | 2,11% |
| **v_16_** | 2,665 | 0,117 | 4,39% | 2,668 | 0,114 | 4,26% |  |  | 0,003 | 2,91% |
| **v_17_** | 0,426 | 0,101 | 23,64% | 0,442 | 0,087 | 19,60% |  |  | 0,014 | 14,10% |
| **v_18_** | 0,426 | 0,079 | 18,42% | 0,417 | 0,063 | 15,17% |  |  | 0,015 | 19,48% |
| **v_21_** | 8,698 | 3,407 | 39,17% |  | - | - |  |  | - | - |
|  |  |  |  |  |  |  |  |  |  |  |
| **Mean** | 3,133 | 0,699 | 32,31% | 2,585 | 0,202 | 14,32% |  |  | 0,497 | 71,09% |

Max: Maximum value of the estimated flux along time;

MIS: Interval size for each estimated fluxes (mean value along time);

Diff: Difference between determined and overdetermined cases;

^a^ in [mM/(dx10^9^xcells)]; ^b^ the interval size for each estimated flux is expressed w.r.t. its maximum value.
