# Supplementary material for: A procedure for the estimation over time of metabolic fluxes in scenarios where measurements are uncertain and/or insufficient
Source: BMC Bioinformatics. 2007 Oct 30;8:421. doi: 10.1186/1471-2105-8-421 (PMC2212668; doi:10.1186/1471-2105-8-421)
Supplement: Additional file 8 — Analysis of the effect of the uncertainty of each measured flux. Details and datasets of the indirect and direct analysis of the effect of the uncertainty of each measured flux. [file 1471-2105-8-421-S8.doc]

# Effect of the uncertainty of each measured flux

## Indirect analysis

This analysis investigates the effect of the uncertainty of each measured flux over the imprecision of the estimated fluxes. Concretely, we will calculate the reduction of the imprecision of the estimated fluxes when the uncertainty of one of the measured flux is decreased a 3%. This calculation will be repeated for each measured flux.

Thus, for each measured flux *v_x_*,

1. We obtain the estimated fluxes at each time instant *k* considering[[1]](#footnote-2):

$\pm5\%$of uncertainty for all the measured fluxes *except v_x_*.

$\pm2\%$of uncertainty for *v_x_*.

1. We calculate the interval size for each estimated flux at each time instant *k, v_c_(k)*

The following equation quantifies the reduction of the imprecision of each estimated flux *v_c_(k)* if the uncertainty of *v_x_* is decreased a 3%:

*Reduction* $=100-\frac{\left(BC-D_{X}\right)}{\left(BC-WC\right)}\cdot100$

D_X_: Interval size of *v_c_(k)*.

WC: Interval size of *v_c_* at time *k* obtained in a worst-case scenario.

BC: Interval size of *v_c_* at time *k* obtained in a best-case scenario.

Hence,

| *Reduction*$=100\%$ | D_X_ = BC, i.e. we can go from the worst to the best case by removing a 3% of uncertainty only to *v_x_*. |
| --- | --- |
| *Reduction* $=0\%$ | D_X_ = WC, i.e. when removing a 3% of uncertainty to the measured flux *v_x_* has no effect. |

*Reference Scenarios:*

worst-case $\pm5\%$of uncertainty for all the measured fluxes (*v_1_*, *v_6_*, *v_7_*, *v_19_* and *v_20_*).

best-case $\pm2\%$of uncertainty for all the measured fluxes (*v_1_*, *v_6_*, *v_7_*, *v_19_* and *v_20_*).

## Indirect analysis: Results

The indirect analysis explained above has been applied to the cultivation of CHO cells. As usually, we used the five measured fluxes (*v_1_* (*G*), *v_6_* (*L*), *v_7_* (*A*), *v_19_* (*NH_4_*) and *v_20_* (*Q*)) and we assumed that the formation of purine and pyrimidine (*v_22_*=0).

The obtained results are given in the following tables and depicted in the figures:


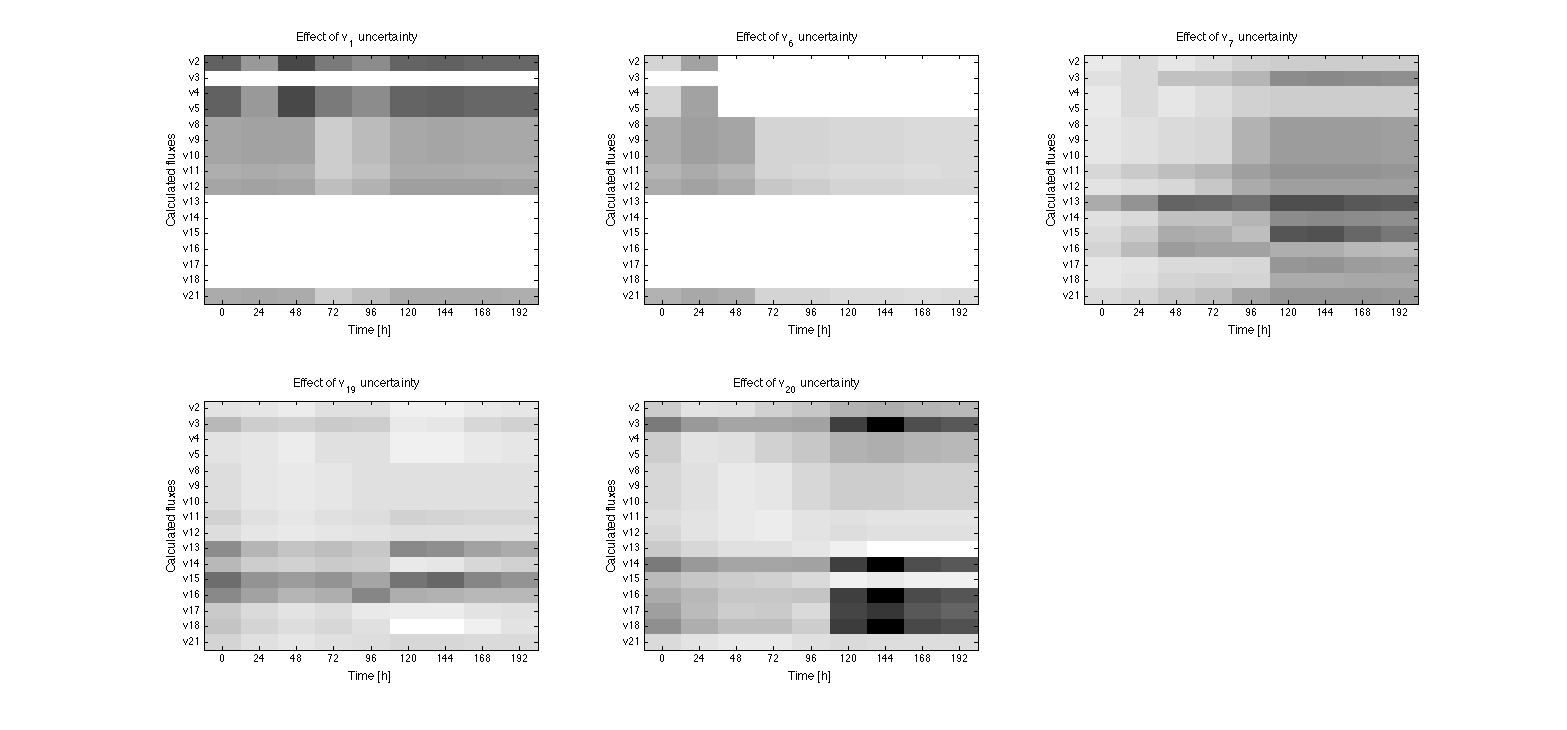


Figure 1. Effect of the uncertainty of each measured flux over the imprecision of the estimated fluxes along time. The figures show the reduction of the imprecision of the estimated (or calculated) fluxes when the uncertainty of the measured flux is decreased a 3%. The reductions are quantified between 0% (white colour) and 100% (black).

Table 1. Effect of the uncertainty of the measured flux *v_1_* over the imprecision of the estimated fluxes along time.

| **Estimated flux** | **0h** | **24h** | **48h** | **72h** | **96h** | **120h** | **144h** | **168h** | **192h** |  |  | **mean** | **median** |
| --- | --- | --- | --- | --- | --- | --- | --- | --- | --- | --- | --- | --- | --- |
| *v_2_* | 65%* | 40% | 75% | 54% | 45% | 63% | 65% | 62% | 61% |  |  | 59% | 62% |
| *v_3_* | 0% | 0% | 0% | 0% | 0% | 0% | 0% | 0% | 0% |  |  | 0% | 0% |
| *v_4_* | 65% | 40% | 75% | 54% | 45% | 63% | 65% | 62% | 61% |  |  | 59% | 62% |
| *v_5_* | 65% | 40% | 75% | 54% | 45% | 63% | 65% | 62% | 61% |  |  | 59% | 62% |
| *v_8_* | 35% | 37% | 37% | 17% | 25% | 34% | 34% | 33% | 33% |  |  | 32% | 34% |
| *v_9_* | 35% | 37% | 37% | 17% | 25% | 34% | 34% | 33% | 33% |  |  | 32% | 34% |
| *v_10_* | 35% | 37% | 37% | 17% | 25% | 34% | 34% | 33% | 33% |  |  | 32% | 34% |
| *v_11_* | 30% | 31% | 30% | 16% | 23% | 31% | 32% | 31% | 30% |  |  | 28% | 30% |
| *v_12_* | 35% | 36% | 35% | 24% | 29% | 37% | 38% | 37% | 37% |  |  | 34% | 36% |
| *v_13_* | 0% | 0% | 0% | 0% | 0% | 0% | 0% | 0% | 0% |  |  | 0% | 0% |
| *v_14_* | 0% | 0% | 0% | 0% | 0% | 0% | 0% | 0% | 0% |  |  | 0% | 0% |
| *v_15_* | 0% | 0% | 0% | 0% | 0% | 0% | 0% | 0% | 0% |  |  | 0% | 0% |
| *v_16_* | 0% | 0% | 0% | 0% | 0% | 0% | 0% | 0% | 0% |  |  | 0% | 0% |
| *v_17_* | 0% | 0% | 0% | 0% | 0% | 0% | 0% | 0% | 0% |  |  | 0% | 0% |
| *v_18_* | 0% | 0% | 0% | 0% | 0% | 0% | 0% | 0% | 0% |  |  | 0% | 0% |
| *v_21_* | 32% | 33% | 32% | 16% | 24% | 32% | 32% | 32% | 31% |  |  | 29% | 32% |
|  | 0% | 0% | 0% | 0% | 0% | 0% | 0% | 0% | 0% |  |  |  |  |
|  |  |  |  |  |  |  |  |  |  |  |  |  |  |
| **mean** | 25% | 21% | 27% | 17% | 18% | 25% | 25% | 24% | 24% |  |  |  |  |
| **median** | 31% | 32% | 31% | 16% | 23% | 32% | 32% | 31% | 31% |  |  |  |  |

* Reduction of the imprecision of the calculated flux when the uncertainty of *v_1_* is decreased a 3%. The reduction is quantified between 0% and 100%.

Table 2. Effect of the uncertainty of the measured flux *v_6_* over the imprecision of the estimated fluxes along time.

| **Estimated flux** | **0h** | **24h** | **48h** | **72h** | **96h** | **120h** | **144h** | **168h** | **192h** |  |  | **mean** | **median** |
| --- | --- | --- | --- | --- | --- | --- | --- | --- | --- | --- | --- | --- | --- |
| *v_2_* | 14%* | 36% | 0% | 0% | 0% | 0% | 0% | 0% | 0% |  |  | 5% | 0% |
| *v_3_* | 0% | 0% | 0% | 0% | 0% | 0% | 0% | 0% | 0% |  |  | 0% | 0% |
| *v_4_* | 14% | 36% | 0% | 0% | 0% | 0% | 0% | 0% | 0% |  |  | 5% | 0% |
| *v_5_* | 14% | 36% | 0% | 0% | 0% | 0% | 0% | 0% | 0% |  |  | 5% | 0% |
| *v_8_* | 32% | 37% | 34% | 14% | 14% | 12% | 13% | 11% | 12% |  |  | 20% | 14% |
| *v_9_* | 32% | 37% | 34% | 14% | 14% | 12% | 13% | 11% | 12% |  |  | 20% | 14% |
| *v_10_* | 32% | 37% | 34% | 14% | 14% | 12% | 13% | 11% | 12% |  |  | 20% | 14% |
| *v_11_* | 28% | 31% | 28% | 14% | 13% | 12% | 12% | 11% | 11% |  |  | 18% | 13% |
| *v_12_* | 32% | 36% | 32% | 20% | 17% | 14% | 14% | 13% | 13% |  |  | 21% | 17% |
| *v_13_* | 0% | 0% | 0% | 0% | 0% | 0% | 0% | 0% | 0% |  |  | 0% | 0% |
| *v_14_* | 0% | 0% | 0% | 0% | 0% | 0% | 0% | 0% | 0% |  |  | 0% | 0% |
| *v_15_* | 0% | 0% | 0% | 0% | 0% | 0% | 0% | 0% | 0% |  |  | 0% | 0% |
| *v_16_* | 0% | 0% | 0% | 0% | 0% | 0% | 0% | 0% | 0% |  |  | 0% | 0% |
| *v_17_* | 0% | 0% | 0% | 0% | 0% | 0% | 0% | 0% | 0% |  |  | 0% | 0% |
| *v_18_* | 0% | 0% | 0% | 0% | 0% | 0% | 0% | 0% | 0% |  |  | 0% | 0% |
| *v_21_* | 29% | 33% | 30% | 14% | 14% | 12% | 12% | 11% | 11% |  |  | 18% | 14% |
|  | 0% | 0% | 0% | 0% | 0% | 0% | 0% | 0% | 0% |  |  |  |  |
| **mean** | 14% | 20% | 12% | 6% | 5% | 5% | 5% | 4% | 4% |  |  |  |  |
| **median** | 14% | 32% | 0% | 0% | 0% | 0% | 0% | 0% | 0% |  |  |  |  |

* Reduction of the imprecision of the calculated flux when the uncertainty of *v_1_* is decreased a 3%. The reduction is quantified between 0% and 100%.

Table 3. Effect of the uncertainty of the measured flux *v_7_* over the imprecision of the estimated fluxes along time.

| **Estimated flux** | **0h** | **24h** | **48h** | **72h** | **96h** | **120h** | **144h** | **168h** | **192h** |  |  | **mean** | **median** |
| --- | --- | --- | --- | --- | --- | --- | --- | --- | --- | --- | --- | --- | --- |
| *v_2_* | 5%* | 11% | 6% | 10% | 16% | 17% | 17% | 17% | 17% |  |  | 13% | 16% |
| *v_3_* | 8% | 12% | 23% | 22% | 28% | 46% | 47% | 45% | 45% |  |  | 31% | 28% |
| *v_4_* | 5% | 11% | 6% | 10% | 16% | 17% | 17% | 17% | 17% |  |  | 13% | 16% |
| *v_5_* | 5% | 11% | 6% | 10% | 16% | 17% | 17% | 17% | 17% |  |  | 13% | 16% |
| *v_8_* | 6% | 9% | 12% | 13% | 30% | 38% | 38% | 39% | 38% |  |  | 25% | 30% |
| *v_9_* | 6% | 9% | 12% | 13% | 30% | 38% | 38% | 39% | 38% |  |  | 25% | 30% |
| *v_10_* | 6% | 9% | 12% | 13% | 30% | 38% | 38% | 39% | 38% |  |  | 25% | 30% |
| *v_11_* | 13% | 18% | 23% | 28% | 37% | 43% | 43% | 43% | 42% |  |  | 32% | 37% |
| *v_12_* | 7% | 10% | 12% | 20% | 32% | 38% | 37% | 38% | 37% |  |  | 26% | 32% |
| *v_13_* | 32% | 43% | 64% | 62% | 58% | 73% | 73% | 69% | 67% |  |  | 60% | 64% |
| *v_14_* | 8% | 12% | 23% | 22% | 28% | 46% | 47% | 45% | 45% |  |  | 31% | 28% |
| *v_15_* | 11% | 18% | 32% | 30% | 24% | 69% | 72% | 62% | 54% |  |  | 41% | 32% |
| *v_16_* | 15% | 25% | 38% | 37% | 36% | 30% | 30% | 27% | 25% |  |  | 29% | 30% |
| *v_17_* | 6% | 8% | 11% | 11% | 12% | 41% | 43% | 39% | 38% |  |  | 23% | 12% |
| *v_18_* | 7% | 9% | 15% | 15% | 14% | 32% | 32% | 33% | 33% |  |  | 21% | 15% |
| *v_21_* | 11% | 15% | 20% | 23% | 35% | 42% | 42% | 42% | 41% |  |  | 30% | 35% |
|  | 0% | 0% | 0% | 0% | 0% | 0% | 0% | 0% | 0% |  |  |  |  |
| **mean** | 10% | 14% | 20% | 21% | 28% | 39% | 39% | 38% | 37% |  |  |  |  |
| **median** | 7% | 11% | 14% | 17% | 29% | 38% | 38% | 39% | 38% |  |  |  |  |

* Reduction of the imprecision of the calculated flux when the uncertainty of *v_7_* is decreased a 3%. The reduction is quantified between 0% and 100%.

Table 4. Effect of the uncertainty of the measured flux *v_19_* over the imprecision of the estimated fluxes along time.

| **Estimated flux** | **0h** | **24h** | **48h** | **72h** | **96h** | **120h** | **144h** | **168h** | **192h** |  |  | **mean** | **median** |
| --- | --- | --- | --- | --- | --- | --- | --- | --- | --- | --- | --- | --- | --- |
| *v_2_* | 8%* | 6% | 4% | 9% | 9% | 2% | 2% | 5% | 6% |  |  | 6% | 6% |
| *v_3_* | 26% | 18% | 16% | 19% | 16% | 5% | 7% | 13% | 16% |  |  | 15% | 16% |
| *v_4_* | 8% | 6% | 4% | 9% | 9% | 2% | 2% | 5% | 6% |  |  | 6% | 6% |
| *v_5_* | 8% | 6% | 4% | 9% | 9% | 2% | 2% | 5% | 6% |  |  | 6% | 6% |
| *v_8_* | 11% | 7% | 4% | 6% | 9% | 9% | 9% | 9% | 9% |  |  | 8% | 9% |
| *v_9_* | 11% | 7% | 4% | 6% | 9% | 9% | 9% | 9% | 9% |  |  | 8% | 9% |
| *v_10_* | 11% | 7% | 4% | 6% | 9% | 9% | 9% | 9% | 9% |  |  | 8% | 9% |
| *v_11_* | 15% | 9% | 6% | 9% | 10% | 15% | 15% | 13% | 12% |  |  | 12% | 12% |
| *v_12_* | 11% | 7% | 4% | 6% | 8% | 9% | 9% | 9% | 8% |  |  | 8% | 8% |
| *v_13_* | 45% | 28% | 21% | 24% | 20% | 46% | 44% | 36% | 32% |  |  | 33% | 32% |
| *v_14_* | 26% | 18% | 16% | 19% | 16% | 5% | 7% | 13% | 16% |  |  | 15% | 16% |
| *v_15_* | 59% | 43% | 39% | 43% | 35% | 56% | 62% | 49% | 43% |  |  | 48% | 43% |
| *v_16_* | 46% | 36% | 27% | 31% | 49% | 30% | 30% | 27% | 26% |  |  | 34% | 30% |
| *v_17_* | 18% | 11% | 8% | 10% | 5% | 3% | 3% | 7% | 9% |  |  | 8% | 8% |
| *v_18_* | 21% | 14% | 11% | 13% | 9% | 0% | 0% | 2% | 7% |  |  | 8% | 9% |
| *v_21_* | 14% | 9% | 6% | 8% | 10% | 13% | 13% | 12% | 11% |  |  | 11% | 11% |
|  | 0% | 0% | 0% | 0% | 0% | 0% | 0% | 0% | 0% |  |  |  |  |
| **mean** | 21% | 14% | 11% | 14% | 14% | 14% | 14% | 14% | 14% |  |  |  |  |
| **median** | 15% | 9% | 6% | 9% | 9% | 9% | 9% | 9% | 9% |  |  |  |  |

* Reduction of the imprecision of the calculated flux when the uncertainty of *v_19_* is decreased a 3%. The reduction is quantified between 0% and 100%.

Table 5. Effect of the uncertainty of the measured flux *v_20_* over the imprecision of the estimated fluxes along time.

| **Estimated flux** | **0h** | **24h** | **48h** | **72h** | **96h** | **120h** | **144h** | **168h** | **192h** |  |  | **mean** | **median** |
| --- | --- | --- | --- | --- | --- | --- | --- | --- | --- | --- | --- | --- | --- |
| *v_2_* | 18% | 8% | 9% | 16% | 20% | 29% | 31% | 28% | 26% |  |  | 20% | 20% |
| *v_3_* | 54% | 39% | 35% | 35% | 36% | 80% | 86% | 73% | 68% |  |  | 56% | 54% |
| *v_4_* | 18% | 8% | 9% | 16% | 20% | 29% | 31% | 28% | 26% |  |  | 20% | 20% |
| *v_5_* | 18% | 8% | 9% | 16% | 20% | 29% | 31% | 28% | 26% |  |  | 20% | 20% |
| *v_8_* | 13% | 8% | 5% | 6% | 12% | 17% | 17% | 16% | 15% |  |  | 12% | 13% |
| *v_9_* | 13% | 8% | 5% | 6% | 12% | 17% | 17% | 16% | 15% |  |  | 12% | 13% |
| *v_10_* | 13% | 8% | 5% | 6% | 12% | 17% | 17% | 16% | 15% |  |  | 12% | 13% |
| *v_11_* | 11% | 7% | 4% | 4% | 8% | 9% | 8% | 8% | 7% |  |  | 7% | 8% |
| *v_12_* | 12% | 8% | 5% | 4% | 8% | 10% | 9% | 9% | 9% |  |  | 8% | 9% |
| *v_13_* | 19% | 13% | 9% | 9% | 6% | 2% | 1% | 1% | 1% |  |  | 7% | 6% |
| *v_14_* | 54% | 39% | 35% | 35% | 36% | 80% | 86% | 73% | 68% |  |  | 56% | 54% |
| *v_15_* | 25% | 19% | 17% | 16% | 11% | 2% | 4% | 1% | 2% |  |  | 11% | 11% |
| *v_16_* | 32% | 27% | 20% | 19% | 22% | 79% | 87% | 74% | 69% |  |  | 48% | 32% |
| *v_17_* | 37% | 25% | 17% | 18% | 12% | 77% | 84% | 69% | 64% |  |  | 45% | 37% |
| *v_18_* | 44% | 30% | 23% | 24% | 17% | 81% | 87% | 75% | 71% |  |  | 50% | 44% |
| *v_21_* | 11% | 7% | 5% | 5% | 9% | 11% | 11% | 10% | 10% |  |  | 9% | 10% |
|  | 0% | 0% | 0% | 0% | 0% | 0% | 0% | 0% | 0% |  |  |  |  |
| **mean** | 24% | 17% | 13% | 15% | 16% | 36% | 38% | 33% | 31% |  |  |  |  |
| **median** | 18% | 8% | 9% | 16% | 12% | 23% | 24% | 22% | 21% |  |  |  |  |

* Reduction of the imprecision of the calculated flux when the uncertainty of *v_20_* is decreased a 3%. The reduction is quantified between 0% and 100%.

## Indirect analysis: Summary


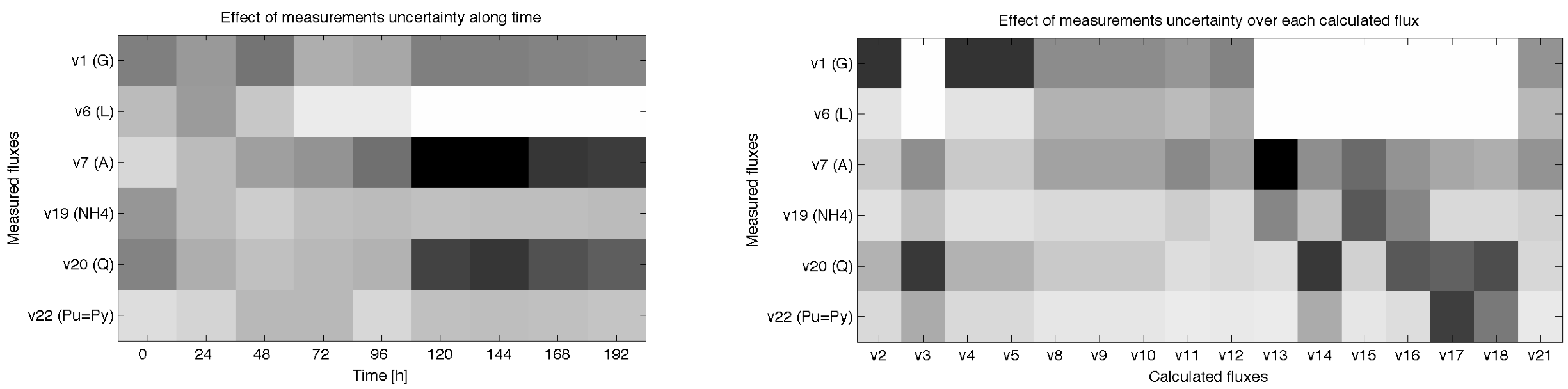


Figure 2. Summary of the effect of the uncertainty of each measured flux over the imprecision of the estimated fluxes along time. (Left) Averaged reduction at each time instant of the imprecision of the estimated (or calculated) fluxes when the uncertainty of the measured flux is decreased a 3%. (Right) Averaged reduction of the imprecision of each estimated (or calculated) fluxes when the uncertainty of the measured flux is decreased a 3%. The reductions are quantified between 0% (white colour) and 100% (black).

Table 6. Averaged reduction at each time instant of the imprecision of the estimated (or calculated) fluxes when the uncertainty of the measured flux is decreased a 3%.

| *Calculated flux* | **0h** | **24h** | **48h** | **72h** | **96h** | **120h** | **144h** | **168h** | **192h** |  |  | **mean** | **median** |
| --- | --- | --- | --- | --- | --- | --- | --- | --- | --- | --- | --- | --- | --- |
| *v_1_* | 25%* | 21% | 27% | 17% | 18% | 25% | 25% | 24% | 24% |  |  | 23% | 24% |
| *v_6_* | 14% | 20% | 12% | 6% | 5% | 5% | 5% | 4% | 4% |  |  | 8% | 5% |
| *v_7_* | 10% | 14% | 20% | 21% | 28% | 39% | 39% | 38% | 37% |  |  | 27% | 28% |
| *v_19_* | 21% | 14% | 11% | 14% | 14% | 14% | 14% | 14% | 14% |  |  | 15% | 14% |
| *v_20_* | 24% | 17% | 13% | 15% | 16% | 36% | 38% | 33% | 31% |  |  | 25% | 24% |
| *v_22_* | 8% | 10% | 15% | 15% | 9% | 13% | 14% | 13% | 13% |  |  | 12% | 13% |

* The reduction is quantified between 0% and 100%.

Table 7. Averaged reduction of the imprecision of each estimated (or calculated) fluxes when the uncertainty of the measured flux is decreased a 3%.

| *Estimated flux* | **-3% unc. v_1_** | **-3% unc. v_6_** | **-3% unc. v_7_** | **-3% unc. v_19_** | **-3% unc. v_20_** | **-3% unc. v_22_** |
| --- | --- | --- | --- | --- | --- | --- |
| *v_2_* | 59%* | 5% | 13% | 6% | 20% | 8% |
| *v_3_* | 0% | 0% | 31% | 15% | 56% | 22% |
| *v_4_* | 59% | 5% | 13% | 6% | 20% | 8% |
| *v_5_* | 59% | 5% | 13% | 6% | 20% | 8% |
| *v_8_* | 32% | 20% | 25% | 8% | 12% | 4% |
| *v_9_* | 32% | 20% | 25% | 8% | 12% | 4% |
| *v_10_* | 32% | 20% | 25% | 8% | 12% | 4% |
| *v_11_* | 28% | 18% | 32% | 12% | 7% | 3% |
| *v_12_* | 34% | 21% | 26% | 8% | 8% | 3% |
| *v_13_* | 0% | 0% | 60% | 33% | 7% | 3% |
| *v_14_* | 0% | 0% | 31% | 15% | 56% | 22% |
| *v_15_* | 0% | 0% | 41% | 48% | 11% | 4% |
| *v_16_* | 0% | 0% | 29% | 34% | 48% | 7% |
| *v_17_* | 0% | 0% | 23% | 8% | 45% | 55% |
| *v_18_* | 0% | 0% | 21% | 8% | 50% | 37% |
| *v_21_* | 29% | 18% | 30% | 11% | 9% | 3% |
|  |  |  |  |  |  |  |
| **mean** | 23% | 8% | 27% | 15% | 25% | 12% |
| **median** | 29% | 5% | 25% | 8% | 16% | 5% |

* The reduction is quantified between 0% and 100%.

## Direct analysis

This analysis is a variant of the previous one. Herein, we will calculate the increase of the imprecision of the estimated fluxes when the uncertainty of one of the measured flux is increased a 3%. This calculation will be repeated for each measured flux.

Thus, for each measured flux *v_x_*,

1. We obtain the estimated fluxes at each time instant *k* considering[[2]](#footnote-3):

$\pm2\%$of uncertainty for all the measured fluxes *except v_x_*.

$\pm5\%$of uncertainty for *v_x_* (an increase of 3%).

1. We calculate the interval size for each estimated flux at each time instant *k, v_c_(k)*

The following equation quantifies the increase of the imprecision of each estimated flux *v_c_(k)* if the uncertainty of *v_x_* is increased a 3%:

*Increase* $=\frac{\left(D_{X}-BC\right)}{\left(WC-BC\right)}\cdot100$

D_X_: Interval size of *v_c_(k)*.

WC: Interval size of *v_c_* at time *k* obtained in a worst-case scenario.

BC: Interval size of *v_c_* at time *k* obtained in a best-case scenario.

Hence,

| *Increase*$=100\%$ | D_X_ = BC, i.e. we can go from the best to the worst case by adding a 3% of uncertainty only to *v_x_*. |
| --- | --- |
| *Increase* $=0\%$ | D_X_ = WC, i.e. when adding a 3% of uncertainty to the measured flux *v_x_* has no effect. |

**Reference Scenarios:**

worst-case $\pm5\%$of uncertainty for all the measured fluxes (*v_1_*, *v_6_*, *v_7_*, *v_19_* and *v_20_*).

best-case $\pm2\%$of uncertainty for all the measured fluxes (*v_1_*, *v_6_*, *v_7_*, *v_19_* and *v_20_*).

## Direct analysis: Results

The direct analysis explained above has been applied to the cultivation of CHO cells. As usually, we used the five measured fluxes (*v_1_* (*G*), *v_6_* (*L*), *v_7_* (*A*), *v_19_* (*NH_4_*) and *v_20_* (*Q*)) and we assumed that the formation of purine and pyrimidine (*v_22_*=0).

The obtained results are given in the following tables and depicted in the figures:


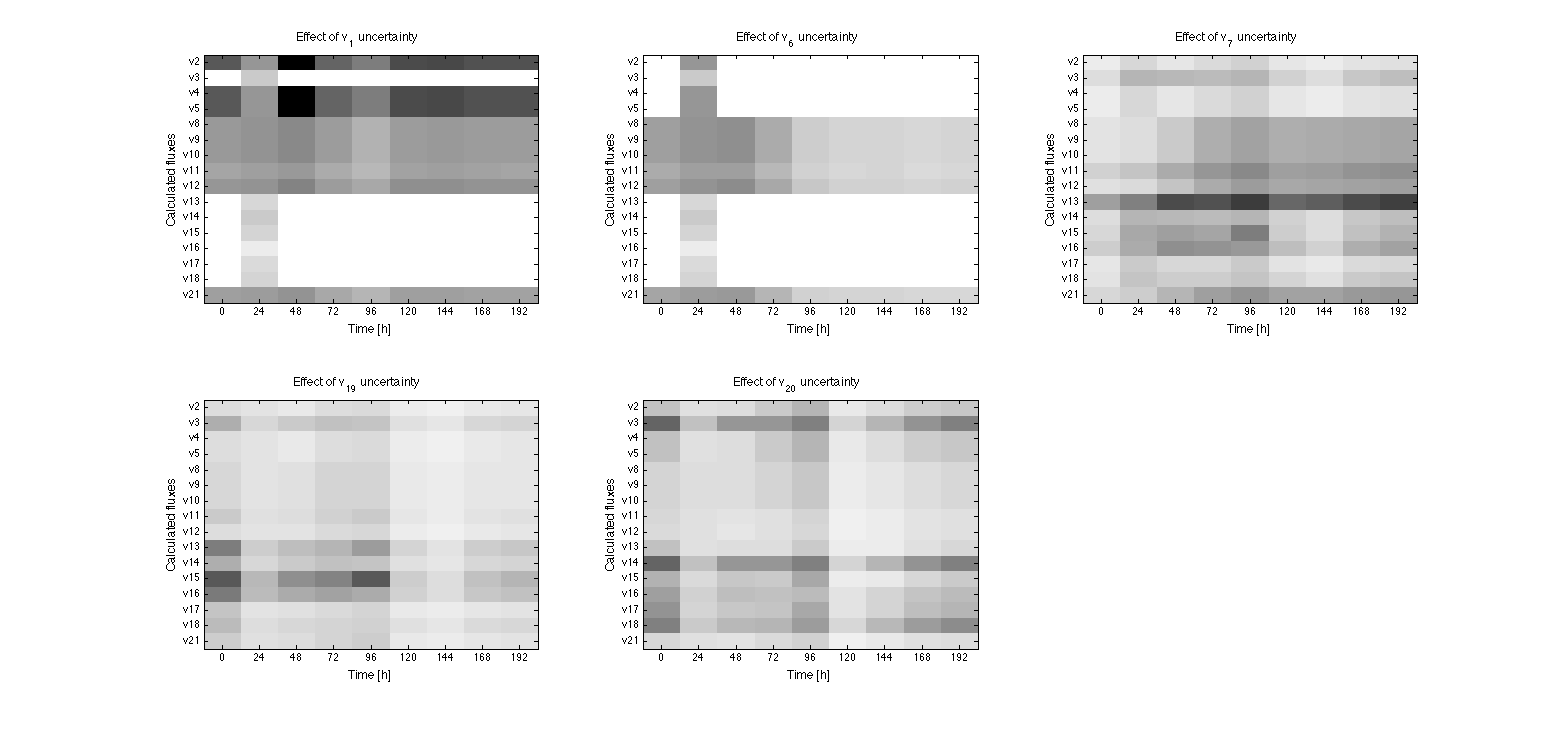


Figure 3. Effect of the uncertainty of each measured flux over the imprecision of the estimated fluxes along time. The figures show the increase of the imprecision of the estimated (or calculated) fluxes when the uncertainty of the measured flux is increased a 3%. The increases are quantified between 0% (white colour) and 100% (black).

Table 8. Effect of the uncertainty of the measured flux *v_1_* over the imprecision of the estimated fluxes along time.

| **Estimated flux** | **0h** | **24h** | **48h** | **72h** | **96h** | **120h** | **144h** | **168h** | **192h** |  |  | **mean** | **median** |
| --- | --- | --- | --- | --- | --- | --- | --- | --- | --- | --- | --- | --- | --- |
| *v_2_* | 59%* | 36% | 75% | 54% | 45% | 63% | 65% | 62% | 61% |  |  | 58% | 61% |
| *v_3_* | 0% | 16% | 0% | 0% | 0% | 0% | 0% | 0% | 0% |  |  | 2% | 0% |
| *v_4_* | 59% | 36% | 75% | 54% | 45% | 63% | 65% | 62% | 61% |  |  | 58% | 61% |
| *v_5_* | 59% | 36% | 75% | 54% | 45% | 63% | 65% | 62% | 61% |  |  | 58% | 61% |
| *v_8_* | 35% | 37% | 40% | 33% | 25% | 34% | 34% | 33% | 33% |  |  | 34% | 34% |
| *v_9_* | 35% | 37% | 40% | 33% | 25% | 34% | 34% | 33% | 33% |  |  | 34% | 34% |
| *v_10_* | 35% | 37% | 40% | 33% | 25% | 34% | 34% | 33% | 33% |  |  | 34% | 34% |
| *v_11_* | 30% | 32% | 35% | 27% | 23% | 31% | 32% | 31% | 30% |  |  | 30% | 31% |
| *v_12_* | 35% | 36% | 42% | 34% | 29% | 37% | 38% | 37% | 37% |  |  | 36% | 37% |
| *v_13_* | 0% | 11% | 0% | 0% | 0% | 0% | 0% | 0% | 0% |  |  | 1% | 0% |
| *v_14_* | 0% | 16% | 0% | 0% | 0% | 0% | 0% | 0% | 0% |  |  | 2% | 0% |
| *v_15_* | 0% | 12% | 0% | 0% | 0% | 0% | 0% | 0% | 0% |  |  | 1% | 0% |
| *v_16_* | 0% | 2% | 0% | 0% | 0% | 0% | 0% | 0% | 0% |  |  | 0% | 0% |
| *v_17_* | 0% | 10% | 0% | 0% | 0% | 0% | 0% | 0% | 0% |  |  | 1% | 0% |
| *v_18_* | 0% | 12% | 0% | 0% | 0% | 0% | 0% | 0% | 0% |  |  | 1% | 0% |
| *v_21_* | 32% | 33% | 36% | 29% | 24% | 32% | 32% | 32% | 31% |  |  | 31% | 32% |
|  | 0% | 0% | 0% | 0% | 0% | 0% | 0% | 0% | 0% |  |  |  |  |
| **mean** | 24% | 25% | 29% | 22% | 18% | 25% | 25% | 24% | 24% |  |  |  |  |
| **median** | 31% | 32% | 35% | 28% | 23% | 32% | 32% | 31% | 31% |  |  |  |  |

* Increase of the imprecision of the calculated flux when the uncertainty of *v_1_* is increased a 3%. The increase is quantified between 0% and 100%.

Table 9. Effect of the uncertainty of the measured flux *v_6_* over the imprecision of the estimated fluxes along time.

| **Estimated flux** | **0h** | **24h** | **48h** | **72h** | **96h** | **120h** | **144h** | **168h** | **192h** |  |  | **mean** | **median** |
| --- | --- | --- | --- | --- | --- | --- | --- | --- | --- | --- | --- | --- | --- |
| *v_2_ IS** | 0%* | 36% | 0% | 0% | 0% | 0% | 0% | 0% | 0% |  |  | 4% | 0% |
| *v_3_* | 0% | 16% | 0% | 0% | 0% | 0% | 0% | 0% | 0% |  |  | 2% | 0% |
| *v_4_* | 0% | 36% | 0% | 0% | 0% | 0% | 0% | 0% | 0% |  |  | 4% | 0% |
| *v_5_* | 0% | 36% | 0% | 0% | 0% | 0% | 0% | 0% | 0% |  |  | 4% | 0% |
| *v_8_* | 32% | 37% | 38% | 28% | 14% | 12% | 13% | 11% | 12% |  |  | 22% | 14% |
| *v_9_* | 32% | 37% | 38% | 28% | 14% | 12% | 13% | 11% | 12% |  |  | 22% | 14% |
| *v_10_* | 32% | 37% | 38% | 28% | 14% | 12% | 13% | 11% | 12% |  |  | 22% | 14% |
| *v_11_* | 28% | 32% | 32% | 23% | 13% | 12% | 12% | 11% | 11% |  |  | 19% | 13% |
| *v_12_* | 33% | 36% | 40% | 28% | 17% | 14% | 14% | 13% | 13% |  |  | 23% | 17% |
| *v_13_* | 0% | 11% | 0% | 0% | 0% | 0% | 0% | 0% | 0% |  |  | 1% | 0% |
| *v_14_* | 0% | 16% | 0% | 0% | 0% | 0% | 0% | 0% | 0% |  |  | 2% | 0% |
| *v_15_* | 0% | 12% | 0% | 0% | 0% | 0% | 0% | 0% | 0% |  |  | 1% | 0% |
| *v_16_* | 0% | 2% | 0% | 0% | 0% | 0% | 0% | 0% | 0% |  |  | 0% | 0% |
| *v_17_* | 0% | 10% | 0% | 0% | 0% | 0% | 0% | 0% | 0% |  |  | 1% | 0% |
| *v_18_* | 0% | 12% | 0% | 0% | 0% | 0% | 0% | 0% | 0% |  |  | 1% | 0% |
| *v_21_* | 29% | 33% | 34% | 24% | 14% | 12% | 12% | 11% | 11% |  |  | 20% | 14% |
|  | 0% | 0% | 0% | 0% | 0% | 0% | 0% | 0% | 0% |  |  |  |  |
| **mean** | 12% | 25% | 14% | 10% | 5% | 5% | 5% | 4% | 4% |  |  |  |  |
| **median** | 0% | 33% | 0% | 0% | 0% | 0% | 0% | 0% | 0% |  |  |  |  |

* Increase of the imprecision of the calculated flux when the uncertainty of *v_6_* is increased a 3%. The increase is quantified between 0% and 100%.

Table 10. Effect of the uncertainty of the measured flux *v_7_* over the imprecision of the estimated fluxes along time.

| **Estimated flux** | **0h** | **24h** | **48h** | **72h** | **96h** | **120h** | **144h** | **168h** | **192h** |  |  | **mean** | **median** |
| --- | --- | --- | --- | --- | --- | --- | --- | --- | --- | --- | --- | --- | --- |
| *v_2_ IS** | 3%* | 11% | 6% | 10% | 13% | 5% | 3% | 7% | 8% |  |  | 7% | 7% |
| *v_3_* | 8% | 24% | 23% | 22% | 24% | 13% | 8% | 17% | 21% |  |  | 18% | 21% |
| *v_4_* | 3% | 11% | 6% | 10% | 13% | 5% | 3% | 7% | 8% |  |  | 7% | 7% |
| *v_5_* | 3% | 11% | 6% | 10% | 13% | 5% | 3% | 7% | 8% |  |  | 7% | 7% |
| *v_8_* | 6% | 9% | 15% | 26% | 31% | 27% | 27% | 29% | 30% |  |  | 22% | 27% |
| *v_9_* | 6% | 9% | 15% | 26% | 31% | 27% | 27% | 29% | 30% |  |  | 22% | 27% |
| *v_10_* | 6% | 9% | 15% | 26% | 31% | 27% | 27% | 29% | 30% |  |  | 22% | 27% |
| *v_11_* | 13% | 18% | 28% | 36% | 40% | 33% | 34% | 37% | 38% |  |  | 31% | 34% |
| *v_12_* | 8% | 10% | 18% | 27% | 33% | 29% | 29% | 32% | 32% |  |  | 24% | 29% |
| *v_13_* | 32% | 44% | 64% | 62% | 69% | 54% | 56% | 64% | 68% |  |  | 57% | 62% |
| *v_14_* | 8% | 24% | 23% | 22% | 24% | 13% | 8% | 17% | 21% |  |  | 18% | 21% |
| *v_15_* | 11% | 28% | 32% | 30% | 45% | 15% | 9% | 20% | 26% |  |  | 24% | 26% |
| *v_16_* | 15% | 27% | 38% | 37% | 34% | 21% | 13% | 26% | 31% |  |  | 27% | 27% |
| *v_17_* | 6% | 15% | 11% | 11% | 16% | 7% | 4% | 10% | 12% |  |  | 10% | 11% |
| *v_18_* | 7% | 19% | 15% | 15% | 18% | 12% | 8% | 16% | 19% |  |  | 14% | 15% |
| *v_21_* | 11% | 15% | 24% | 32% | 37% | 31% | 31% | 34% | 35% |  |  | 28% | 31% |
|  | 0% | 0% | 0% | 0% | 0% | 0% | 0% | 0% | 0% |  |  |  |  |
| **mean** | 9% | 18% | 21% | 25% | 29% | 20% | 18% | 24% | 26% |  |  |  |  |
| **median** | 7% | 15% | 17% | 26% | 31% | 18% | 11% | 23% | 28% |  |  |  |  |

* Increase of the imprecision of the calculated flux when the uncertainty of *v_7_* is increased a 3%. The increase is quantified between 0% and 100%.

Table 11. Effect of the uncertainty of the measured flux *v_19_* over the imprecision of the estimated fluxes along time.

| **Estimated flux** | **0h** | **24h** | **48h** | **72h** | **96h** | **120h** | **144h** | **168h** | **192h** |  |  | **mean** | **median** |
| --- | --- | --- | --- | --- | --- | --- | --- | --- | --- | --- | --- | --- | --- |
| *v_2_ IS** | 9%* | 6% | 4% | 9% | 10% | 3% | 2% | 4% | 5% |  |  | 6% | 5% |
| *v_3_* | 26% | 11% | 16% | 19% | 18% | 8% | 6% | 11% | 12% |  |  | 14% | 12% |
| *v_4_* | 9% | 6% | 4% | 9% | 10% | 3% | 2% | 4% | 5% |  |  | 6% | 5% |
| *v_5_* | 9% | 6% | 4% | 9% | 10% | 3% | 2% | 4% | 5% |  |  | 6% | 5% |
| *v_8_* | 11% | 7% | 8% | 12% | 13% | 4% | 2% | 5% | 6% |  |  | 7% | 7% |
| *v_9_* | 11% | 7% | 8% | 12% | 13% | 4% | 2% | 5% | 6% |  |  | 7% | 7% |
| *v_10_* | 11% | 7% | 8% | 12% | 13% | 4% | 2% | 5% | 6% |  |  | 7% | 7% |
| *v_11_* | 15% | 8% | 9% | 13% | 15% | 5% | 3% | 6% | 7% |  |  | 9% | 8% |
| *v_12_* | 8% | 7% | 6% | 9% | 11% | 3% | 2% | 4% | 5% |  |  | 6% | 6% |
| *v_13_* | 45% | 14% | 21% | 24% | 33% | 13% | 6% | 15% | 17% |  |  | 21% | 17% |
| *v_14_* | 26% | 11% | 16% | 19% | 18% | 8% | 6% | 11% | 12% |  |  | 14% | 12% |
| *v_15_* | 59% | 23% | 39% | 43% | 59% | 14% | 8% | 19% | 24% |  |  | 32% | 24% |
| *v_16_* | 46% | 22% | 27% | 31% | 28% | 14% | 9% | 17% | 19% |  |  | 24% | 22% |
| *v_17_* | 18% | 7% | 8% | 10% | 12% | 4% | 3% | 6% | 7% |  |  | 8% | 7% |
| *v_18_* | 21% | 8% | 11% | 13% | 14% | 8% | 5% | 10% | 11% |  |  | 11% | 11% |
| *v_21_* | 14% | 7% | 9% | 13% | 15% | 5% | 3% | 6% | 7% |  |  | 9% | 7% |
|  | 0% | 0% | 0% | 0% | 0% | 0% | 0% | 0% | 0% |  |  |  |  |
| **mean** | 21% | 10% | 12% | 16% | 18% | 6% | 4% | 8% | 10% |  |  |  |  |
| **median** | 15% | 7% | 9% | 12% | 13% | 4% | 3% | 6% | 7% |  |  |  |  |

* Increase of the imprecision of the calculated flux when the uncertainty of *v_19_* is increased a 3%. The increase is quantified between 0% and 100%.

Table 12. Effect of the uncertainty of the measured flux *v_20_* over the imprecision of the estimated fluxes along time.

| **Estimated flux** | **0h** | **24h** | **48h** | **72h** | **96h** | **120h** | **144h** | **168h** | **192h** |  |  | **mean** | **median** |
| --- | --- | --- | --- | --- | --- | --- | --- | --- | --- | --- | --- | --- | --- |
| *v_2_ IS** | 19%* | 8% | 9% | 16% | 24% | 4% | 9% | 14% | 17% |  |  | 13% | 14% |
| *v_3_* | 54% | 20% | 35% | 35% | 44% | 12% | 24% | 37% | 44% |  |  | 34% | 35% |
| *v_4_* | 19% | 8% | 9% | 16% | 24% | 4% | 9% | 14% | 17% |  |  | 13% | 14% |
| *v_5_* | 19% | 8% | 9% | 16% | 24% | 4% | 9% | 14% | 17% |  |  | 13% | 14% |
| *v_8_* | 13% | 8% | 9% | 12% | 17% | 3% | 5% | 9% | 11% |  |  | 10% | 9% |
| *v_9_* | 13% | 8% | 9% | 12% | 17% | 3% | 5% | 9% | 11% |  |  | 10% | 9% |
| *v_10_* | 13% | 8% | 9% | 12% | 17% | 3% | 5% | 9% | 11% |  |  | 10% | 9% |
| *v_11_* | 11% | 7% | 6% | 8% | 12% | 2% | 3% | 6% | 8% |  |  | 7% | 7% |
| *v_12_* | 9% | 8% | 5% | 8% | 12% | 2% | 3% | 6% | 7% |  |  | 7% | 7% |
| *v_13_* | 19% | 8% | 9% | 9% | 16% | 2% | 2% | 8% | 11% |  |  | 9% | 9% |
| *v_14_* | 54% | 20% | 35% | 35% | 44% | 12% | 24% | 37% | 44% |  |  | 34% | 35% |
| *v_15_* | 25% | 10% | 17% | 16% | 29% | 3% | 5% | 11% | 16% |  |  | 14% | 16% |
| *v_16_* | 32% | 13% | 20% | 19% | 21% | 6% | 12% | 19% | 22% |  |  | 18% | 19% |
| *v_17_* | 37% | 12% | 17% | 18% | 29% | 6% | 13% | 20% | 24% |  |  | 20% | 18% |
| *v_18_* | 44% | 15% | 23% | 24% | 34% | 11% | 23% | 34% | 40% |  |  | 27% | 24% |
| *v_21_* | 11% | 8% | 7% | 9% | 14% | 2% | 4% | 7% | 9% |  |  | 8% | 8% |
|  | 0% | 0% | 0% | 0% | 0% | 0% | 0% | 0% | 0% |  |  |  |  |
| **mean** | 24% | 11% | 14% | 17% | 24% | 5% | 10% | 16% | 19% |  |  |  |  |
| **median** | 19% | 8% | 9% | 16% | 23% | 4% | 7% | 13% | 16% |  |  |  |  |

* Increase of the imprecision of the calculated flux when the uncertainty of *v_20_* is increased a 3%. The increase is quantified between 0% and 100%.

## Indirect analysis: Summary


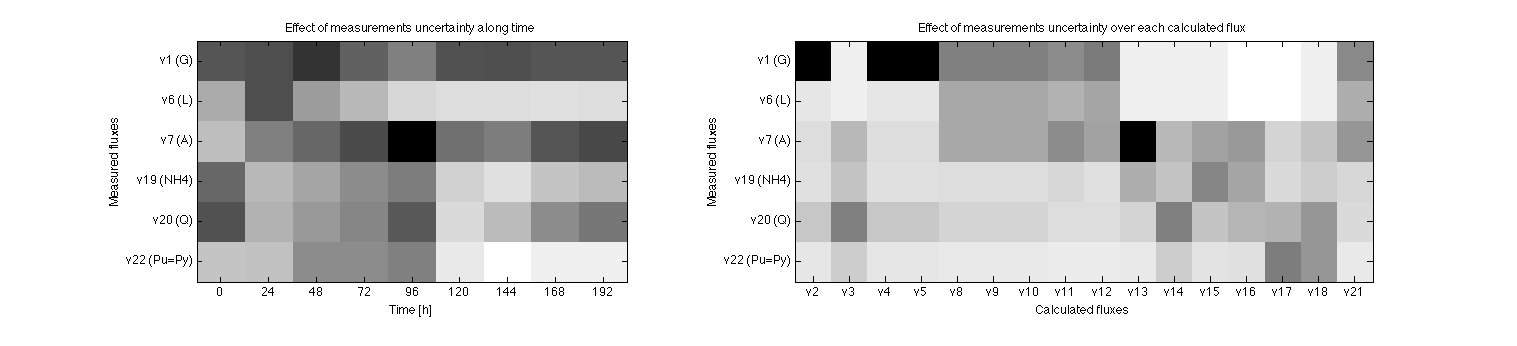


Figure 4. Summary of the effect of the uncertainty of each measured flux over the imprecision of the estimated fluxes along time. (Left) Averaged increase at each time instant of the imprecision of the estimated (or calculated) fluxes when the uncertainty of the measured flux is increased a 3%. (Right) Averaged increase of the imprecision of each estimated (or calculated) fluxes when the uncertainty of the measured flux is increased a 3%. The increase are quantified between 0% (white colour) and 100% (black).

Table 13. Averaged increase at each time instant of the imprecision of the estimated (or calculated) fluxes when the uncertainty of the measured flux is increased a 3%.

| *Calculated flux* | **0h** | **24h** | **48h** | **72h** | **96h** | **120h** | **144h** | **168h** | **192h** |  |  | **mean** | **median** |
| --- | --- | --- | --- | --- | --- | --- | --- | --- | --- | --- | --- | --- | --- |
| *v_1_* | 24%* | 25% | 29% | 22% | 18% | 25% | 25% | 24% | 24% |  |  | 24% | 24% |
| *v_6_* | 12% | 25% | 14% | 10% | 5% | 5% | 5% | 4% | 4% |  |  | 9% | 5% |
| *v_7_* | 9% | 18% | 21% | 25% | 29% | 20% | 18% | 24% | 26% |  |  | 21% | 21% |
| *v_19_* | 21% | 10% | 12% | 16% | 18% | 6% | 4% | 8% | 10% |  |  | 12% | 10% |
| *v_20_* | 24% | 11% | 14% | 17% | 24% | 5% | 10% | 16% | 19% |  |  | 16% | 16% |
| *v_22_* | 8% | 8% | 16% | 16% | 18% | 3% | 1% | 2% | 2% |  |  | 8% | 8% |

* The increase is quantified between 0% and 100%.

Table 14. Averaged increase of the imprecision of each estimated (or calculated) fluxes when the uncertainty of the measured flux is increased a 3%.

| *Calculated flux* | **+3% unc. v_1_** | **+3% unc. v_6_** | **+3% unc. v_7_** | **+3% unc. v_19_** | **+3% unc. v_20_** | **+3% unc. v_22_** |
| --- | --- | --- | --- | --- | --- | --- |
| *v_2_* | 58% | 4% | 7% | 6% | 13% | 5% |
| *v_3_* | 2% | 2% | 18% | 14% | 34% | 11% |
| *v_4_* | 58% | 4% | 7% | 6% | 13% | 5% |
| *v_5_* | 58% | 4% | 7% | 6% | 13% | 5% |
| *v_8_* | 34% | 22% | 22% | 7% | 10% | 4% |
| *v_9_* | 34% | 22% | 22% | 7% | 10% | 4% |
| *v_10_* | 34% | 22% | 22% | 7% | 10% | 4% |
| *v_11_* | 30% | 19% | 31% | 9% | 7% | 3% |
| *v_12_* | 36% | 23% | 24% | 6% | 7% | 2% |
| *v_13_* | 1% | 1% | 57% | 21% | 9% | 3% |
| *v_14_* | 2% | 2% | 18% | 14% | 34% | 11% |
| *v_15_* | 1% | 1% | 24% | 32% | 14% | 5% |
| *v_16_* | 0% | 0% | 27% | 24% | 18% | 6% |
| *v_17_* | 1% | 1% | 10% | 8% | 20% | 35% |
| *v_18_* | 1% | 1% | 14% | 11% | 27% | 28% |
| *v_21_* | 31% | 20% | 28% | 9% | 8% | 3% |
|  |  |  |  |  |  |  |
| **mean** | 24% | 9% | 21% | 12% | 16% | 8% |
| **median** | 31% | 4% | 22% | 8% | 13% | 5% |

* The increase is quantified between 0% and 100%.

1. Applying the procedure introduced in this paper. [↑](#footnote-ref-2)
2. Applying the procedure introduced in this paper. [↑](#footnote-ref-3)
