# Supplementary material for: A procedure for the estimation over time of metabolic fluxes in scenarios where measurements are uncertain and/or insufficient
Source: BMC Bioinformatics. 2007 Oct 30;8:421. doi: 10.1186/1471-2105-8-421 (PMC2212668; doi:10.1186/1471-2105-8-421)
Supplement: Additional file 4 — Mathematical model (SBML and matlab). The zip file contains the mathematical model of the metabolism of CHO cells in three different formats: a matlab script, an SBML model and a metatool file. [file 1471-2105-8-421-S4.zip › Model.doc]

Additional file 4: Mathematical model (SBML and matlab).

The mathematical model is provided in different formats:

- *«Provost04_matlab.m»* Matlab model (preferred).

This matlab script includes the stoichiometric matrixes *S* and *S_ξ_*, which were used to obtain the results described in the article. Therefore, this is the most validated version of the model.

- «*Provost04_level1.xml*» and «*Provost04_level2.xml*» SBML models (level 1 and level 2).

The SBML model has been tested with *JDesigner* and *Yana*.

- «*Provost04_metatool.dat*» *Metatool* format model.

The model in the format used by *Metatool*.

**JDesigner** is a Win32 application which allows one to draw a biochemical network and export the network in the form of SBML. (Webpage: <http://sbw.kgi.edu/software/jdesigner.htm>)

**MATLAB** is a high-level language and interactive environment that enables you to perform computationally intensive tasks faster than with traditional programming languages. (Webpage: <http://www.mathworks.com/products/matlab/>)

**Metatool** is a program for detection of independent pathways from substrates to products and for disassembly of large biochemical networks into smaller subnets. (Webpage: <http://pinguin.biologie.uni-jena.de/bioinformatik/networks/>)

**YANA** is a user friendly software package for the analysis of metabolic networks. It is capable of performing elementary mode analysis on those networks and incorporates new algorithms for mapping elementary mode activities to enzyme activities and vice versa. (Webpage: <http://yana.bioapps.biozentrum.uni-wuerzburg.de/>)

The SBML model represented in Jdesigner.
