# Supplementary material for: A procedure for the estimation over time of metabolic fluxes in scenarios where measurements are uncertain and/or insufficient
Source: BMC Bioinformatics. 2007 Oct 30;8:421. doi: 10.1186/1471-2105-8-421 (PMC2212668; doi:10.1186/1471-2105-8-421)
Supplement: Additional file 5 — Implementation of the Flux Spectrum Approach. The zip file contains a matlab script which implements the method to estimate the non-measured fluxes with the Flux Spectrum Approach and a simple example that illustrates how to use it. [file 1471-2105-8-421-S5.zip › FSAfunction.doc]

Additional file 5: Implementation of the Flux-spectrum approach.

The method to estimate the non-measured fluxes with FSA has been implemented in a *matlab* script. This script and a very simple example are provided in the files «*vspectrumR.m*» and «*example_of_FSA_function.m*».

- FSA method implementation: «*vspectrumR.m*».

function [Vmin,Vmax,Flag] = vspectrumR(N,irrev,vm_ind,Vm_mini,Vm_maxi)

% Estimate the unknown fluxes: Nu*vu=-Nm*vm

%

% N: Stoichiometric matrix (N*v=0)

% irrev: Vector with indexes of the irreversible fluxes

% vm_ind: Vector with indexes of the measured fluxes

% Vm_mini: Vector with minimum values of the measured fluxes

% Vm_mini: Vector with maximum values of the measured fluxes

%

% % Example:

% % Set of measured fluxes.

% ind=[1,3,4,7,8]; % indexes of the measured fluxes

% vm=[0.91;0.88;0.92;0.89;1.85]; % measured values

%

% % Uncertainty around measurements

% vmin=0.95*vm;

% vmax=1.05*vm;

%

% % Estimation of the non-measured fluxes with the FSA

% [vmin2,vmax2,Flag] = vspectrumR(N,irrev,ind,vmin,vmax);

%

% Francisco Llaneras Estrada

% Dept. of Systems Engineering and Control , Technical University of Valencia

% e-mail: frallaes@doctor.upv.es and kikollan@gmail.com, Web Page: http://f.llaneras.googlepages.com

%

% Llaneras F. and Pico J. (2007). Procedure to the estimation of metabolic fluxes

% over time in scenarios where measurements are uncertain and/or insufficient.

% Tittle may change (submitted).

%

% Llaneras F. and Pico J. (2006). An interval approach for dealing with flux

% distributions and elementary modes activity patterns. Journal of

% theoretical biology (in press).

%%%%%%%%%%%%%%%%%%%%%%%%%%%%%%%%%%%%%%%%%%%%%%

% 1: Division between Nu y Nm (Nu*vu=-Nm*vm)

%%%%%%%%%%%%%%%%%%%%%%%%%%%%%%%%%%%%%%%%%%%%%%

Nu=[];Nm=[];Vm_min=[];Vm_max=[];irrev_u=[];j=1;

n=size(N,2); % number of fluxes

% for each flux...

for i=1:1:n

ind=find(vm_ind==i);

if(isempty(ind))	% then it is an unknown flux

Nu=[Nu N(:,i)];

irrev_u=[irrev_u irrev(1,i)];

else	% then it is a measured flux

Nm=[Nm N(:,i)];

Vm_min=[Vm_min;Vm_mini(j)];

Vm_max=[Vm_max;Vm_maxi(j)];

j=j+1;

end

end

%%%%%%%%%%%%%%%%%%%%%%%%%%%%%%%%%%

% 2: Calculate [Nm*Vm]max and [Nm*Vm]min

%%%%%%%%%%%%%%%%%%%%%%%%%%%%%%%%%%

NVmax=[];

NVmin=[];

Nm=-Nm;

for i=1:1:size(Nm,1) %rows

maxs=[];

mins=[];

for j=1:1:size(Nm,2) %columns

if(Nm(i,j)>0)	% if sign of Nm is +

maxs=[maxs Vm_max(j)]; %[Nm*Vm]max=f(Vmax)

mins=[mins Vm_min(j)]; %[Nm*Vm]min=f(Vmin)

else	% if sign of Nm is -

maxs=[maxs Vm_min(j)]; %[Nm*Vm]max=f(Vmin)

mins=[mins Vm_max(j)]; %[Nm*Vm]min=f(Vmax)

end

end

NVmax(i)=Nm(i,:)*maxs';

NVmin(i)=Nm(i,:)*mins';

end

NVmax=NVmax'; NVmin=NVmin';

%%%%%%%%%%%%%%%%%%%%%%%%%%%%%%%%%%%%%%%%%%%%%%%%%

% 3: Estimate the non-measured fluxes with FSA

%%%%%%%%%%%%%%%%%%%%%%%%%%%%%%%%%%%%%%%%%%%%%%%%%

u=size(Nu,2); % Dimensions

Vu_max=[];Vu_min=[];Flag=[];

% for each unknown (non-measured) flux....

for i=1:1:u

ind=zeros(1,u);

ind(i)=1;

% calculate its minimum value with linear programming (minimize v)

[min_i,a,flag_min]=linprog(ind,[-Nu;Nu;-diag(irrev_u)],[-NVmin;NVmax;zeros(u,1)]);

Vu_min=[Vu_min;min_i(i)];

ind=zeros(1,u);

ind(i)=-1;

% calculate its maximum value with linear programming (minimize -v)

[max_i,a,flag_max]=linprog(ind,[-Nu;Nu;-diag(irrev_u)],[-NVmin;NVmax;zeros(u,1)]);

Vu_max=[Vu_max;max_i(i)];

Flag=[Flag;flag_min flag_max]; % Information about convergence

end

%%%%%%%%%%%%%%%%%%%%%%%%%%%%%%%%%%%%%%%%%%%

% 4: Reconstruct vector with all fluxes V

%%%%%%%%%%%%%%%%%%%%%%%%%%%%%%%%%%%%%%%%%%%

Vmin=[];Vmax=[];j=1;k=1;

for i=1:1:n

ind=find(vm_ind==i);

if(isempty(ind))	% if v is not a measured fluxes

Vmin=[Vmin;Vu_min(j)]; % I take its values from the unknowns

Vmax=[Vmax;Vu_max(j)];

j=j+1;

else	% if v is a measured fluxes

Vmin=[Vmin;Vm_min(k)]; % I take its values from the measurements

Vmax=[Vmax;Vm_max(k)];

k=k+1;

end

end

- An example «*example_of_FSA_function.m*».

% A simple example of how to use the FSA method: vspectrumR.m

%

% The used metabolic network is depicted in an additional file

% of the article (Llaneras F. and Pico J., 2007).

%

% Francisco Llaneras Estrada

% Dept. of Systems Engineering and Control (DISA)

% Technical University of Valencia (UPV)

% e-mail: frallaes@doctor.upv.es and kikollan@gmail.com

% Web Page: http://f.llaneras.googlepages.com

%

% References

% Llaneras F. and Pico J. (2007). Procedure to the estimation of metabolic fluxes over time in scenarios where measurements are uncertain and/or insufficient. Tittle may change (submitted).

%

% Llaneras F. and Pico J. (2006). An interval approach for dealing with flux distributions and elementary modes activity patterns. Journal of theoretical biology (in press).

% Stoichiometric Matrix.

N=[1 -1 -1 0 0 0 0 0;

0 0 1 -1 -1 0 0 0;

0 0 0 1 0 -1 1 0;

0 1 0 0 1 1 0 -1];

% Reversibility of fluxes.

irrev=[ones(1,6)]; % all are irreversible

irrev(4)=0; % except flux 4

% Set of measured fluxes.

ind=[1,3,4,7,8]; % index of the measured fluxes

vm=[0.91;0.88;0.92;0.89;1.85]; % measured values

% Uncertainty around measurements

vmin=0.95*vm; %vmin=vm.*[0.95 0.95 0.95 0.95 0.95]';

vmax=1.05*vm; %vmax=vm.*[1.05 1.05 1.05 1.05 1.05]';

% Estimation of the non-measured fluxes with the FSA

[vmin2,vmax2,Flag] = vspectrumR(N,irrev,ind,vmin,vmax);

% vmin2: vector of minimum values for each flux

% vmax2: vector of maximum values for each flux

% Flag: Information about convergence of LP problems (see help linprog)

% Make a figure (not very important code)

figure()

n=size(vmin2,1);

v_med2=0.5*(vmin2+vmax2);

range_down2=v_med2-vmin2;

range_up2=vmax2-v_med2;

h1=errorbar([1:n],v_med2,range_down2,range_up2,'ob','LineWidth',2)

hold on

h2=errorbar([1;3;4;7;8],[v_med2(1);v_med2(3);v_med2(4);v_med2(7:8)],[range_down2(1);range_down2(3);range_down2(4);range_down2(7:8)],[range_up2(1);range_up2(3);range_up2(4);range_up2(7:8)],'ok','LineWidth',2)

set([h1;h2],'Marker','none')

xlabel('Reaction (1-8)');ylabel('Flux [flux units]');

set(gca,'XTick',[1 2 3 4 5 6 7 8]); set(gca,'FontSize',12);

grid
